# Naringenin, a Food-Derived Flavanone, Suppresses ITGA11-Associated Gastric Cancer Progression via the FAK/PI3K/AKT/mTOR Axis

**DOI:** 10.3390/cancers18111712

**Published:** 2026-05-24

**Authors:** Qiang Li, Guiyang Ye, Fangfang Chen, Qiushuang Wang, Junfeng Yan, Yi Wang, Qiang Tong

**Affiliations:** 1 Department of Gastrointestinal Surgery I Section, Renmin Hospital of Wuhan University, Wuhan 430060, China; qiangli@whu.edu.cn (Q.L.); guiyangye@whu.edu.cn (G.Y.); wqs8540@sina.com (Q.W.); junfengyan@whu.edu.cn (J.Y.); 2 Department of Pathology, Renmin Hospital of Wuhan University, Wuhan 430060, China; fangfangchen@whu.edu.cn; 3 Translational Clinical Immunology Key Laboratory, Sichuan Provincial People’s Hospital, Chengdu 610072, China; 4 Department of Dermatology Surgery, Sichuan Provincial People’s Hospital, University of Electronic Science and Technology of China, Chengdu 610072, China

**Keywords:** Naringenin, gastric cancer, ITGA11, FAK/PI3K/AKT/mTOR axis

## Abstract

(1) Background: Gastric cancer (GC) remains a leading cause of cancer-related mortality worldwide. Aberrant remodeling of the extracellular matrix (ECM) is a hallmark of GC progression; however, the mechanisms by which GC cells sense and exploit ECM cues remain unclear. (2) Methods: ITGA11 was identified through integrative bioinformatic analyses. Its expression, clinical significance, and association with ECM-related signatures were evaluated in GC tissues and public datasets. The function of ITGA11 and its role in regulating the FAK/PI3K/AKT/mTOR pathway were investigated using in vitro and in vivo assays, and the inhibitory effect of Naringenin on ITGA11-associated oncogenic activity was assessed. (3) Results: ITGA11 was upregulated in GC tissues and correlated with an ECM-related gene signature, aggressive clinicopathological features and poor patient survival. ITGA11 promoted malignant phenotypes of GC cells in vitro and in vivo. Importantly, molecular docking and target engagement assays suggested a potential interaction between Naringenin and ITGA11. Functional experiments showed that Naringenin attenuated ITGA11-associated oncogenic activity by reducing ITGA11 levels, suppressing pathway activation, and inhibiting malignant phenotypes. (4) Conclusions: Our findings identify ITGA11 as a potential prognostic biomarker and functional driver of GC progression and suggest that Naringenin may represent a promising bioactive compound for modulating the ITGA11/FAK/PI3K/AKT/mTOR axis in GC.

## Simple Summary

Gastric cancer is a common and deadly cancer, and better markers and treatment strategies are urgently needed. The tissue environment surrounding cancer cells can strongly influence how tumors grow and spread, but the molecules that help gastric cancer cells respond to this environment are not fully understood. In this study, we found that integrin alpha 11 is increased in gastric cancer and is associated with more aggressive disease and poorer patient survival. We further showed that integrin alpha 11 promotes gastric cancer cell proliferation, migration, and invasion in vivo and in vitro by activating the FAK/PI3K/AKT/mTOR signaling pathway. Importantly, we found that Naringenin may reduce these harmful effects by lowering integrin alpha 11 activity. These findings suggest that integrin alpha 11 may be a useful biomarker and potential therapeutic target, and that Naringenin may offer a promising strategy for future gastric cancer research.

## 1. Introduction

Gastric cancer (GC) remains a leading cause of cancer-related mortality worldwide, largely owing to metastatic progression and the limited efficacy of current therapies for advanced disease [1,2,3]. Despite advances in targeted therapy and immunotherapy, the prognosis of patients with invasive or metastatic GC remains poor. This situation highlights an urgent need to identify novel molecular drivers and actionable therapeutic targets [4,5].

Tumor progression and metastasis reflect the dynamic interplay between cancer cells and their surrounding stroma [6,7]. In the tumor microenvironment of GC, pathological remodeling of the extracellular matrix (ECM) often manifests as a desmoplastic reaction. This remodeling contributes to tumor aggressiveness by altering matrix composition, organization, and mechanical properties [8,9,10,11,12]. Beyond providing structural support, the remodeled ECM acts as an active signaling platform. It delivers biochemical and biomechanical cues that are primarily sensed through integrin-dependent mechanotransduction. These signals activate pathways that drive cancer cell proliferation, invasion, metastasis, and therapeutic resistance [13,14,15].

Integrins are transmembrane α/β heterodimers that mediate bidirectional communication between cells and the ECM [16,17]. By recognizing specific ECM ligands, including collagen, fibronectin, and laminin, integrins transduce extracellular mechanical and biochemical cues into intracellular signaling pathways that regulate adhesion, migration, proliferation, and survival [18,19]. Dysregulated integrin expression and activation have been widely implicated in tumor growth, invasion, metastasis, and therapy resistance [20,21]. In GC, several integrins, such as α5β1 and β3, have been linked to disease progression through activation of oncogenic signaling pathways, including FAK/SRC and NF-κB [22,23]. Given the collagen-rich desmoplastic microenvironment of GC, collagen-binding integrins are of particular interest. Among these, ITGA11, encoding the α11 integrin subunit, was prioritized for ligand screening because our bioinformatic analyses identified it as associated with GC progression and poor prognosis. Despite preliminary studies on ITGA11 in GC, its precise functional role and therapeutic potential remain largely unexplored [24,25,26,27].

Recent studies have demonstrated that extracts and bioactive compounds derived from dietary and edible sources can exert significant antitumor effects in cancers [28,29]. Naringenin, a major flavanone widely present in citrus fruits and other plants, is one such compound that has attracted considerable attention [30]. Naringenin has been reported to possess antioxidant, anti-inflammatory, and anticancer activities. It can suppress malignant phenotypes by modulating multiple signaling pathways involved in tumor growth and metastasis [31,32]. Nevertheless, the therapeutic relevance of Naringenin in GC remains to be fully elucidated. In particular, its potential effects on ECM-associated oncogenic signaling and the molecular basis for its action in GC remain poorly understood.

However, whether Naringenin interferes with ITGA11-associated oncogenic signaling in GC remains unknown. Therefore, in the present study, we systematically investigated the clinical significance, biological function, and underlying molecular mechanisms of ITGA11 in GC. We show that ITGA11 is upregulated in GC and is associated with aggressive clinicopathological features and poor patient outcomes. Functional analyses further demonstrate that ITGA11 promotes GC progression by activating the FAK/PI3K/AKT/mTOR signaling axis. To identify potential therapeutic compounds interfering with ITGA11-associated signaling, we performed virtual screening using the PubChem database. Naringenin emerged as a suitable candidate, and subsequent molecular docking analysis and target engagement assays further supported its potential binding to ITGA11. Importantly, Naringenin could potentially bind and suppress ITGA11 expression, attenuate ITGA11-associated signaling, and inhibit malignant phenotypes in GC models. Collectively, these findings identify ITGA11 as a mediator of ECM-driven GC progression and highlight its potential as a therapeutic target in GC.

## 2. Materials and Methods

### 2.1. Patient Samples

A retrospective cohort of 60 patients with primary gastric adenocarcinoma who underwent curative-intent resection at Renmin Hospital of Wuhan University between 2020 and 2022 was included in this study. The study protocol was approved by the Ethics Committee of Renmin Hospital of Wuhan University (Approval No. WDRY2022-K227), and written informed consent was obtained from all participants. Clinicopathological data were collected from medical records (Table S1), and overall survival data were obtained through follow-up. Formalin-fixed, paraffin-embedded (FFPE) tumor tissues and matched adjacent non-tumor tissues located at least 5 cm from the tumor margin were used for immunohistochemistry. Ten randomly selected matched FFPE tumor/adjacent tissue pairs were used for immunofluorescence analysis. In addition, five paired fresh tumor and adjacent non-tumor tissue samples were collected immediately after surgery, snap-frozen in liquid nitrogen, and stored at −80 °C for qPCR and Western blotting.

### 2.2. Reagents and Equipment

In this study, Naringenin (cat. no. HY-N0100; MedChemExpress, Shanghai, China) and Defactinib (cat. no. HY-12289; MedChemExpress) were utilized. For in vitro assays, Naringenin was dissolved in dimethyl sulfoxide (DMSO; cat. no. GC20006; Servicebio, Wuhan, China) to create stock solutions, which were subsequently aliquoted and stored at −20 °C. Throughout all experiments, the concentration of DMSO in the culture medium was maintained at or below 0.1% (*v*/*v*), with an equivalent volume of DMSO serving as the vehicle control. Additional reagents and equipment are listed in Supplementary Tables S2 and S3.

### 2.3. Identification of Prognosis-Associated Differentially Expressed Genes

RNA-seq data and associated clinical information for gastric cancer were obtained from The Cancer Genome Atlas (TCGA) database. Differentially expressed genes (DEGs) between tumor and adjacent normal tissues were identified using the “DESeq2” R package (v1.52.0), applying thresholds of |log2 fold change (FC)| > 1 and an adjusted *p*-value < 0.05. A volcano plot was constructed to illustrate the differential expression patterns. Simultaneously, genes significantly correlated with overall survival (OS) were identified through univariate Cox proportional hazards regression analysis (*p* < 0.05). The candidate gene set was defined as the intersection of the survival-associated genes and the DEGs. The candidate gene set underwent additional analysis to elucidate its potential biological functions and enriched pathways. Gene Ontology (GO) and Kyoto Encyclopedia of Genes and Genomes (KEGG) pathway enrichment analyses were conducted utilizing the “clusterProfiler” R package (v4.20.0). Terms and pathways with an adjusted *p*-value of less than 0.05 were considered statistically significant. Additionally, a Protein–Protein Interaction (PPI) network of the candidate genes was constructed using the STRING database (version 11.5) and visualized with Cytoscape software (version 3.9.1) to identify key hub genes.

### 2.4. Evaluation of ECM-Related Gene Signature and Correlation Analysis

Based on the enrichment analysis that identified ECM-related pathways among the candidate genes, we focused on ITGA11 as a key gene to assess ECM signaling activity. ITGA11 was subsequently evaluated using an ECM scoring system. This analysis was conducted utilizing the “GSVA” R package. The ECM score was quantified through two complementary approaches using RNA-seq data from the TCGA-STAD dataset. A manually curated gene set, comprising 18 established ECM-related genes, was employed: COL1A1, COL5A2, COL12A1, COL8A1, BGN, VCAN, FBN3, MATN3, MFAP2, LOX, P4HA3, SPARC, SERPINE1, VTN, ADAM12, ADAMTS2, ADAMTS18, and FGG. Initially, a Z-score-based signature score was calculated by normalizing the expression of each gene across samples and averaging these values per sample. This calculation was performed separately for the entire cohort and the tumor-only subset, identified via TCGA barcode suffixes [33]. Subsequently, a pathway-centric single-sample Gene Set Enrichment Analysis (ssGSEA) score was computed for the tumor subset utilizing the “GSVA” R package (v2.6.2), with the specified gene set and normalization procedures applied. The correlation between ITGA11 expression and the ssGSEA score was evaluated using Spearman’s rank correlation coefficient, while comparisons of scores between ITGA11-high and ITGA11-low groups were conducted using the Wilcoxon rank-sum test. To further elucidate the role of ITGA11 within the ECM network, a correlation analysis was performed between ITGA11 expression and a set of established core ECM genes. Pairwise Pearson correlation coefficients were calculated for all tumor samples, with statistical significance determined via the *t*-test (*p* < 0.05). The results were subsequently visualized as a correlation matrix using the “ggcorrplot” R package (v0.1.4.1).

### 2.5. Differential Expression and Pathway Enrichment Analysis Based on ITGA11 Stratification

To elucidate the biological processes regulated by ITGA11, tumor samples from the TCGA-STAD cohort were categorized into high and low-expression groups based on the median expression level of ITGA11. DEGs between these groups were identified utilizing the “DESeq2” R package, applying a significance threshold of |log2 fold change (FC)| > 1 and an adjusted *p*-value < 0.05. Subsequently, the identified DEGs underwent functional annotation. GO and KEGG pathway enrichment analyses were conducted using the “clusterProfiler” R package. Terms and pathways with an adjusted *p*-value (FDR) < 0.05 were deemed significantly enriched. Subsequently, the prognostic significance of ITGA11 was assessed through Kaplan–Meier survival analysis, employing log-rank tests to evaluate overall survival.

### 2.6. Targeted Analysis of the ITGA11-Associated Signaling Axis

To elucidate the specific role of ITGA11 within the proposed mechanistic pathway, a comprehensive analysis was conducted focusing on the genes involved in the FAK/PI3K/AKT/mTOR axis, including PTK2 (FAK), SRC, PIK3CA, AKT1, RPS6KB1, EIF4EBP1, and MTOR. Initially, the differential expression of these genes was examined between gastric tumor tissues and adjacent normal tissues within the TCGA-STAD cohort. Subsequently, the prognostic significance of each gene was assessed through Kaplan–Meier survival analysis, employing log-rank tests to evaluate overall survival. Patients were stratified into high and low expression groups according to the median expression level of the respective gene. Finally, to investigate potential co-regulatory relationships, pairwise Pearson correlation analyses were performed between the expression levels of ITGA11 and each of the aforementioned pathway genes across all tumor samples. All statistical analyses and visualizations were executed using R software (version 4.1.1).

### 2.7. Virtual Screening and Molecular Docking of Candidate Compounds Targeting ITGA11

Virtual screening and molecular docking were performed to identify potential ITGA11-binding compounds. The full-length amino acid sequence of human ITGA11 was obtained from the UniProtKB database using the ITGA11 gene name. Since no experimentally resolved PDB structure of ITGA11 was available in the RCSB Protein Data Bank, the ITGA11 protein sequence was submitted to the SWISS-MODEL server for homology modeling. The optimal predicted structure was selected according to the model quality assessment provided by SWISS-MODEL and saved in PDB format for subsequent docking analysis. The full-length ITGA11 model was used as the receptor. The receptor structure was prepared by adding hydrogen atoms and assigning charges, followed by conversion into PDBQT format using AutoDock 4.0. The protonation states of the receptor and ligands were assigned at physiological pH 7.4. A curated compound library containing 1200 candidate compounds, including 800 natural products and 400 approved small-molecule drugs, was constructed from the PubChem database. Ligands were prepared by adding hydrogen atoms and charges, defining them as ligands, checking rotatable bonds and torsion angles, and saving the processed structures in PDBQT format for docking. Molecular docking was performed using AutoDock Vina 1.2.6. The docking grid box was centered at x = 15.9963857357, y = 1.80330186713, and z = −9.54085312797, with dimensions of 40.045481683 × 40.045481683 × 40.045481683 Å. AutoDock Vina was run with exhaustiveness = 8, num_modes = 9, energy_range = 3 kcal/mol, and seed = 8. The receptor was treated as rigid, whereas ligand rotatable bonds were kept flexible. Compounds were ranked according to their predicted binding energies. Compounds with predicted binding energies lower than −4.0 kcal/mol were considered to have potential binding affinity and were retained for further analysis. Lower binding energies were interpreted as indicating stronger predicted interactions between the ligand and ITGA11. Naringenin was selected for detailed docking analysis based on its favorable binding energy and potential biological relevance. The optimal docking pose was defined as the conformation with the lowest predicted binding energy. Docking poses and ligand–receptor interactions were visualized and analyzed using PyMOL 3.1 and Discovery Studio 2019.

### 2.8. Cell Lines and Cell Culture

In this study, the human gastric epithelial cell line GES-1, along with a selection of human gastric cancer cell lines (HGC-27, AGS, MGC-803, SGC-7901), were employed. These cell lines were procured from the Cell Bank of the Chinese Academy of Sciences (Shanghai, China). Each cell line was cultured in its respective recommended medium: GES-1 and SGC-7901 were maintained in Roswell Park Memorial Institute 1640 medium (RPMI 1640; cat. no. SH30605.01; HyClone, Logan, UT, USA), AGS cells were cultured in Ham’s F-12K medium (Ham’s F-12K; cat. no. G4560; Servicebio), while HGC-27 and MGC-803 were cultured in Dulbecco’s Modified Eagle Medium (DMEM; cat. no. SH30022.01; HyClone). All media were supplemented with 10% (*v*/*v*) heat-inactivated fetal bovine serum (FBS; cat. no. A5256701; Gibco, Grand Island, NY, USA) and 1% (*v*/*v*) penicillin-streptomycin (cat. no. 15140122; Gibco). The cells were incubated in a humidified environment at 37 °C with a 5% CO_2_ atmosphere and were routinely passaged upon reaching 80–90% confluence. All cell lines were authenticated by short tandem repeat (STR) profiling and tested negative for mycoplasma contamination before use.

### 2.9. Immunohistochemistry Analysis

The expression of the ITGA11 protein was evaluated using immunohistochemistry (IHC) on formalin-fixed, paraffin-embedded (FFPE) tissue sections. After deparaffinization and antigen retrieval with citrate buffer (pH 6.0), the sections were blocked and incubated overnight at 4 °C with anti-ITGA11 primary antibody (dilution 1:200; cat. no. 68350; Cell Signaling Technology, Danvers, MA, USA). Detection was achieved using a horseradish peroxidase (HRP)-conjugated secondary antibody (cat. no. G1213; Servicebio) and diaminobenzidine (DAB; cat. no. G1212; Servicebio) chromogen, followed by counterstaining with hematoxylin (cat. no. G1004; Servicebio). The staining was independently assessed by two pathologists who were blinded to the clinical data. A semi-quantitative score, ranging from 0 to 12, was determined by multiplying the staining intensity (intensity: 0 = negative, 1 = weak, 2 = moderate, 3 = strong) by the percentage of positive tumor cells (proportion: 0 = 0%, 1 = 1–25%, 2 = 26–50%, 3 = 51–75%, 4 = 76–100%). The median score of the cohort was employed to categorize the samples into ITGA11-high and ITGA11-low expression groups.

### 2.10. Immunofluorescence Staining

Immunofluorescence (IF) staining was conducted on 4 μm thick formalin-fixed, paraffin-embedded (FFPE) sections obtained from 10 pairs of matched gastric cancer and adjacent normal tissues to assess the expression and localization of the ITGA11 protein. Briefly, following deparaffinization, rehydration, and antigen retrieval, the sections were blocked with 5% bovine serum albumin (BSA; cat. no. GC305010; Servicebio) and subsequently incubated overnight at 4 °C with a primary antibody targeting ITGA11 (dilution 1:100; cat. no. 68350; Cell Signaling Technology). After washing, the sections were incubated with an Alexa Fluor 488-conjugated secondary antibody (dilution 1:500; cat. no. GB25301; Servicebio) for 1 h at room temperature in the dark. Nuclei were counterstained with 4′,6-diamidino-2-phenylindole (DAPI; cat. no. G1012; Servicebio). Finally, the slides were mounted using an anti-fade mounting medium. Fluorescence images were captured using a confocal laser scanning microscope (Leica TCS SP8 confocal microscope, Wetzlar, Germany). For the IF analysis of HGC-27 cells, the cells were initially seeded onto sterile glass coverslips placed in a 24-well plate, at a density of 1 × 10^4^ cells per well. The cells were then processed in accordance with the experimental protocol. Following a PBS wash, the cells were fixed using 4% paraformaldehyde at room temperature for 15 min. This was followed by a blocking step with 5% bovine serum albumin for 1 h. All subsequent procedures were performed according to the same protocol described above. Fluorescence images were captured using an upright microscope (OLYMPUS BX53, Tokyo, Japan). The relative fluorescence intensity was quantified using ImageJ software (version 1.8.0).

### 2.11. Hematoxylin and Eosin (H&E) Staining

For histological examination, xenograft tumor tissues were subjected to fixation in 4% paraformaldehyde (cat. no. G1101; Servicebio), followed by paraffin embedding and sectioning at a thickness of 4 μm. The sections underwent deparaffinization in xylene (cat. no. 10023428; Sinopharm Group Co., Ltd., Shanghai, China) and rehydration through a graded ethanol series. Subsequent staining with hematoxylin and eosin (H&E; cat. no. G1076; Servicebio) was performed in accordance with established protocols. The stained sections were then dehydrated, mounted, and examined under an upright microscope to evaluate morphological characteristics. For the selection of representative histological fields, all H&E-stained sections were first reviewed by an experienced pathologist who was blinded to the group allocation. Representative fields were selected from viable tumor regions with preserved tissue morphology, avoiding areas with extensive necrosis, hemorrhage, tissue folding, sectioning artifacts, or poorly preserved staining. Necrotic regions were excluded from the histological evaluation. Four tumor samples were analyzed in each group, and five randomly selected non-necrotic fields were captured from each tumor section. Thus, a total of 20 fields per group were evaluated for histological assessment.

### 2.12. Western Blotting

Total protein was extracted from snap-frozen gastric tissues or cultured cells utilizing Radio Immunoprecipitation Assay (RIPA) lysis buffer (cat. no. BL504A; Biosharp, Hefei, China) supplemented with protease and phosphatase inhibitors. The protein concentration was quantified using a BCA protein assay kit (cat. no. BL521A SET; Biosharp). Subsequently, equal amounts of protein (20–40 μg) were resolved by SDS-PAGE on 6–12.5% gels (cat. no. PG220, PG222, PG223; EpiZyme, Shanghai, China) and subsequently transferred onto PVDF membranes (cat. no. IPVH00010; Millipore, Burlington, MA, USA). The membranes were blocked with 5% non-fat milk (cat. no. GC310001; Servicebio) in TBST (cat. no. G2150; Servicebio) for 1 h at room temperature, followed by overnight incubation at 4 °C with primary antibodies. The primary antibodies used in this study included ITGA11 (1:1000; cat. no. 68350; Cell Signaling Technology), p-FAK (1:1000; cat. no. 3283; Cell Signaling Technology), FAK (1:1000; cat. no. 3285; Cell Signaling Technology), p-PI3K (p85 (Tyr458)/p55 (Tyr199), 1:1000; cat. no. 4228; Cell Signaling Technology), PI3K (1:1000; cat. no. Abs147226; Absin, Shanghai, China), p-AKT (Ser473, 1:5000; cat. no. 28731-1-AP; Proteintech, Wuhan, China), AKT (1:2000; cat. no. Ab8805; Abcam, Cambridge, UK), p-mTOR (1:1000; cat. no. 5536; Cell Signaling Technology), mTOR (1:1000; cat. no. 2983; Cell Signaling Technology), Bax (1:1000; cat. no. 2772; Cell Signaling Technology), Bcl-2 (1:1000; cat. no. 3498; Cell Signaling Technology), Vimentin (1:2000; cat. no. ab92547; Abcam), E-cadherin (1:2000; cat. no. ab40772; Abcam), N-Cadherin (1:2000; cat. no. ab76011; Abcam) and β-actin (1:4000; cat. no. GB15003; Servicebio) as a loading control. The membranes were then incubated with appropriate HRP-conjugated secondary antibody (1:10000; GB23303; Servicebio) for 1 h at room temperature. Protein bands were visualized using an enhanced chemiluminescence (ECL; cat. no. BL523B; Biosharp) detection system and imaged with a chemiluminescence imaging system. Densitometric analysis was conducted using ImageJ software (version 1.8.0).

### 2.13. Quantitative PCR

Total RNA was isolated from tissue samples or cultured cells utilizing the TRIzol reagent (cat. no. 15596018CN; Thermo, Waltham, MA, USA), adhering to the protocol provided by the manufacturer. The concentration and purity of the RNA were evaluated using spectrophotometric methods. Complementary DNA (cDNA) was synthesized from 1 µg of total RNA employing the SweScript RT II First Strand cDNA Synthesis Kit (inclusive of gDNA Remover) (cat. no. G3333; Servicebio). Quantitative polymerase chain reaction (qPCR) was conducted using the 2 × Universal Blue SYBR Green qPCR Master Mix (with UDG) (cat. no. G3328, Servicebio) on a QuantStudio system. The thermal cycling conditions were as follows: an initial denaturation step at 95 °C for 30 s, followed by 40 cycles of 95 °C for 5 s and 60 °C for 30 s. Gene expression levels were normalized to β-actin, which served as an internal control. Relative expression levels were determined using the 2^−ΔΔCt^ method. The sequences of the primer pairs employed are provided in Supplementary Table S4.

### 2.14. Overexpression

The complete coding sequences of human ITGA11 and PTK2 (encoding FAK) were inserted into the pcDNA3.1(+) vector (cat. no. V79020; Thermo) to construct the overexpression plasmids pcDNA3.1-ITGA11 (oeITGA11) and pcDNA3.1-FAK (oeFAK), respectively. The empty pcDNA3.1(+) vector was utilized as a negative control. Gastric cancer cells were cultured in suitable plates and allowed to reach 60–70% confluence prior to transfection. Transfections were performed using the respective plasmids and Lipofectamine 3000 reagent (cat. no. TL301-01; Vazyme, Nanjing, China), adhering to the manufacturer’s instructions. Six to eight hours post-transfection, the transfection medium was replaced with fresh complete medium. To assess overexpression efficiency, cells were collected 48–72 h after transfection. The mRNA and protein expression levels of ITGA11 and FAK were evaluated using qPCR and Western blot analysis, as previously described.

### 2.15. Silencing by shRNA or siRNA

To investigate loss-of-function phenotypes, both stable and transient knockdown methodologies were utilized. For stable knockdown, short hairpin RNAs (shRNAs) targeting ITGA11 and PTK2 (FAK) were engineered and inserted into a lentiviral vector (cat. no. 8453; Addgene, Watertown, MA, USA). A non-targeting shRNA (shNC) served as a negative control. Lentiviral particles were generated in HEK-293T cells, and gastric cancer cells were subsequently infected in the presence of polybrene (8 µg/mL; cat. no. sc-134220; Santa Cruz Biotechnology, Dallas, TX, USA). Stable cell populations were selected using puromycin (2 µg/mL; cat. no. sc-108071; Santa Cruz Biotechnology) over a period of 7–10 days. For transient knockdown, a validated small interfering RNA (siRNA) specifically targeting PTK2 (siFAK) and a non-targeting control siRNA (siNC) were introduced into cells via Lipofectamine 3000 reagent, following the manufacturer’s protocol. Knockdown efficiency was assessed at both the mRNA and protein levels using quantitative PCR and Western blot analysis 48–72 h post-transfection or following selection. Detailed sequences of the siRNAs and shRNAs are provided in Table S4.

### 2.16. Cell Counting Kit-8 (CCK-8) Assay

Cell viability was evaluated utilizing the Cell Counting Kit-8 (CCK-8) assay. Cells were plated in 96-well plates at a density of 2 × 10^3^ cells per well and allowed to adhere overnight. Subsequently, treatments were administered in accordance with the predetermined experimental protocol. At 0, 24, 48, and 72 h post-treatment, 10 µL of CCK-8 reagent (cat. no. BS350B; Biosharp) was added to each well, followed by a 2-h incubation at 37 °C. Absorbance was then measured at 450 nm using a microplate reader. All experimental conditions were conducted with a minimum of three replicates.

### 2.17. Wound Healing Assay

The cell migration capacity was assessed via a wound healing assay. In this procedure, cells were cultured in 6-well plates until they reached near confluence. A sterile 200 µL pipette tip was employed to introduce a linear scratch, referred to as a “wound,” across the cell monolayer. Subsequently, the cells were rinsed with PBS to eliminate any dislodged cells, and a serum-free medium was introduced. Images of the wound area were captured at the same designated location immediately post-scratch (0 h) and after a 48-h incubation period using an inverted microscope (OLYMPUS IX71, Tokyo, Japan). The wound area remaining after 48 h was measured and quantified utilizing ImageJ software. Each experiment was conducted with a minimum of three independent replicates.

### 2.18. Transwell Assay

The migratory and invasive capabilities of cells were assessed utilizing Transwell chambers (cat. no. 3464; Corning, Corning, NY, USA) with an 8 μm pore size. For the invasion assay, the upper chamber was pre-coated with a Matrigel matrix (diluted 1:8 in serum-free medium; cat. no. 356234; Solarbio, Beijing, China). Cells were trypsinized, resuspended in serum-free medium, and seeded into the upper chamber at a density of 1 × 10^5^ cells per well. The lower chamber was supplemented with medium containing 10% FBS to serve as a chemoattractant. Following a 48-h incubation period at 37 °C, non-migrated or non-invaded cells on the upper membrane surface were carefully removed using a cotton swab. Cells that had migrated through the membrane were fixed with 4% paraformaldehyde, stained with 0.1% crystal violet (cat. no. BL802A; Biosharp), and visualized using an inverted microscope. The quantification of migrated or invaded cells was performed by counting cells in three randomly selected fields per chamber. The migration assay was conducted using the same methodology, excluding the Matrigel coating. Each experiment was conducted in triplicate and independently replicated at least three times.

### 2.19. Colony Formation

Cells were seeded into 6-well plates at a density of 1000 cells per well in a complete medium and allowed to adhere overnight. Subsequently, the cells were cultured for a period of 14 days, with the medium being replenished every 3 to 4 days. Following the incubation period, the cells underwent washing with phosphate-buffered saline (PBS), fixation with 4% paraformaldehyde for 15 min, and staining with 0.1% crystal violet for 30 min at ambient temperature. After staining, colonies were observed and manually counted under a microscope. A colony was defined as a clustered cell population containing 50 or more cells. The colony formation results were quantified as the absolute number of colonies per well. Each experimental condition was conducted in triplicate and replicated in a minimum of three independent experiments.

### 2.20. Drug Affinity Responsive Target Stability (DARTS) Assay

Protein lysates were aliquoted and incubated with varying concentrations of Naringenin at room temperature for 1 h to allow potential binding interactions. Proteolytic digestion was then initiated by adding Pronase (MCE, HY-114158 A, China) at a 1:3000 enzyme-to-protein ratio, with a final enzyme concentration of 10 mg/mL, and carried out for 10 min. The reaction was terminated to halt further proteolysis. To assess the stabilization of target proteins by Naringenin, samples were subjected to SDS-PAGE on 10% gels, followed by Western blotting with an ITGA11 antibody (cat. no. 68350; Cell Signaling Technology) at a dilution of 1:2000. Band intensities were quantified using ImageJ software, enabling evaluation of Naringenin-induced protection of ITGA11 from proteolytic degradation [34,35].

### 2.21. Cellular Thermal Shift Assay (CETSA)

Total cellular proteins were extracted and divided into aliquots, with one set incubated with Naringenin for 1 h at room temperature. The treated samples were then distributed into separate microcentrifuge tubes and subjected to heat treatment for 3 min at incremental temperatures of 37, 40, 43, 46, 49, 52, 55, 58, 61, and 64 °C. Following thermal denaturation, the samples were centrifuged at 20,000× *g* for 20 min to separate soluble proteins. The supernatants were collected and stored at −80 °C prior to analysis by Western blot, enabling evaluation of Naringenin-induced thermal stabilization of ITGA11 [36].

### 2.22. Animal Model

All animal experiments were conducted in accordance with the guidelines set forth by the Animal Ethics Committee of Renmin Hospital of Wuhan University (Approval No.: 20250402B). Male BALB/c nude mice, aged 4 to 6 weeks, were maintained under specific pathogen-free conditions (n = 4 per cage). To assess the in vivo antitumor effect of Naringenin in ITGA11-overexpressing GC xenografts, AGS cells with overexpression of ITGA11 (oeITGA11) were subcutaneously injected into BALB/c nude mice. Cells in the logarithmic growth phase were collected and resuspended in a 1:1 (*v*/*v*) mixture of PBS and Matrigel at a concentration of 1 × 10^7^ cells/mL. Subsequently, 100 μL of this suspension, containing 1 × 10^6^ cells, was subcutaneously injected into the right flank of each mouse, with four mice allocated per group. When tumor volumes reached approximately 100–150 mm^3^, mice were randomly assigned to two experimental groups using a computer-generated random number sequence, with each mouse receiving a unique number. Animals were then allocated to groups in ascending numerical order (n = 4 per group): (1) oeITGA11 with vehicle (2% DMSO in PBS), and (2) oeITGA11 with Naringenin. Naringenin was dissolved in the 2% DMSO in PBS and administered via intraperitoneal injection at a dosage of 50 mg/kg every day until the study endpoint. To further assess the role of ITGA11 in vivo, AGS cells with ITGA11 knockdown (shITGA11-2) and their corresponding control cells (shNC) were prepared and injected into the mice as previously described. Tumor dimensions, specifically length (L) and width (W), were measured every 3 days, and tumor volume was calculated using the formula (L × W^2^)/2. At the conclusion of the experiment, the mice were euthanized, and the tumors were excised, weighed, and subjected to further analysis. Subcutaneous tumor inoculation and drug administration were performed in a double-blind manner, such that both the personnel performing the procedures and those recording the data were unaware of the group assignments and treatment conditions. Following euthanasia, xenograft tumors were surgically excised under sterile conditions, photographed, and weighed. To enable both molecular and histological analyses, each tumor was divided into fresh and fixed tissue fractions. Fresh tumor tissues were rapidly frozen in liquid nitrogen and stored at −80 °C until protein and RNA extraction for Western blotting and qRT-PCR validation. In parallel, the remaining tumor tissues were fixed in 4% paraformaldehyde, processed for paraffin embedding, and sectioned for hematoxylin and eosin staining and immunohistochemical detection of Ki-67. The procedures for sample collection, tissue processing, histological assessment, and data analysis were performed in a blinded manner with respect to group allocation. Standardized protocols were strictly followed throughout to minimize experimental variability and potential bias. Mice were euthanized at the predefined study endpoint or earlier if humane endpoint criteria were met, including tumor diameter > 1.5 cm, ulceration or necrosis, rapid body weight loss (>15%), impaired mobility, or signs of distress.

### 2.23. Statistical Analysis

Statistical analyses were performed using GraphPad Prism 8.0 and R 4.1.1. Data from at least three independent biological experiments are presented as mean ± s.d. unless otherwise indicated. Normality was assessed before parametric testing. Comparisons between two groups were performed using an unpaired two-tailed Student’s *t*-test for normally distributed data or the Mann–Whitney U test for non-normally distributed data. Paired samples were analyzed using a paired *t*-test or Wilcoxon signed-rank test. Comparisons among multiple groups were performed using one-way ANOVA followed by Tukey’s multiple-comparison test. Effect size analyses were conducted for all applicable statistical tests (Table S5). Correlations were assessed using Pearson or Spearman correlation analysis, as appropriate. Survival curves were generated using the Kaplan–Meier method and compared using the log-rank test. In bioinformatic analyses, FDR-adjusted *p* values < 0.05 were considered statistically significant. For all other analyses, *p* < 0.05 was considered statistically significant. For all other experiments, significance levels were denoted as follows: ns, not significant, * *p* < 0.05; ** *p* < 0.01; *** *p* < 0.001; **** *p* < 0.0001.

## 3. Results

### 3.1. Bioinformatic Analyses Identify ECM Organization as a Prominently Enriched Pathway and Link ITGA11 to ECM-Related Activity in Gastric Cancer

**Figure 1:**
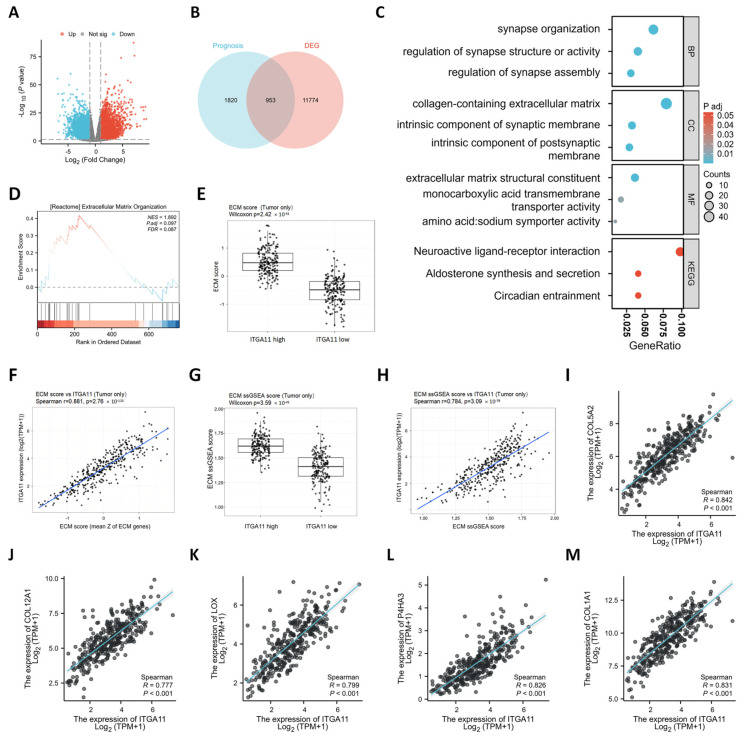
Bioinformatic identification of ITGA11 as a key ECM-related gene in GC. (**A**) Volcano plot of DEGs between tumor and normal tissues in the TCGA-STAD cohort. (**B**) Venn diagram showing the overlap between DEGs and prognosis-related genes. (**C**) GO and KEGG enrichment analyses of overlapping genes. (**D**) Gene Set Enrichment Analysis (GSEA) of overlapping genes. (**E**) ECM score in ITGA11-high versus ITGA11-low tumors. (**F**) Correlation between ITGA11 expression and ECM score. (**G**) ECM ssGSEA score in ITGA11-high versus ITGA11-low tumors. (**H**) Correlation between ITGA11 expression and ECM ssGSEA score. (**I**–**M**) Correlation analyses of ITGA11 with ECM genes. (**I**) COL5A2; (**J**) COL12A1; (**K**) LOX; (**L**) P4HA3; (**M**) COL1A1. A total of 32 normal samples and 375 tumor samples in TCGA-STAD cohort were analyzed.

To elucidate the key molecular features associated with GC progression, we performed integrated bioinformatic analyses of the TCGA-STAD cohort (Figure S1A). Differential expression analysis identified 12,727 significantly DEGs (|log2 fold change (FC)| > 1 and an adjusted *p*-value < 0.05) between tumor and normal tissue (Figure 1A), while univariate Cox regression analysis revealed 2773 genes (*p*-value < 0.05) associated with patient prognosis. The intersection of these two datasets yielded 953 genes that were both differentially expressed and prognostically relevant (Figure 1B). Functional enrichment analysis of these candidate genes revealed strong involvement in ECM-related biology. Representative Gene Ontology terms included the collagen-containing extracellular matrix and extracellular matrix structural constituent (Figure 1C), and Gene Set Enrichment Analysis (GSEA) further demonstrated significant enrichment of the extracellular matrix organization pathway (Figure 1D). Genes within this pathway were subsequently mapped onto a protein–protein interaction (PPI) network, supporting the relevance of aberrant ECM remodeling in GC (Figure S1B). We next focused on ITGA11 as a candidate ECM-associated molecule. To assess its relationship with ECM activity, we applied two complementary quantitative approaches. Within the tumor subset, samples with high ITGA11 expression exhibited significantly higher ECM scores than those with low ITGA11 expression (Figure 1E), and ITGA11 expression was positively correlated with ECM score (Figure 1F). This association was further supported by a pathway-centric single-sample GSEA (ssGSEA)-based ECM enrichment score (Figure 1G,H). Similar trends were observed in the overall TCGA-STAD cohort (Figure S1C–F). Moreover, ITGA11 expression exhibited significant positive correlations with key ECM-related genes, including COL5A2, COL12A1, LOX, P4HA3, and COL1A1 (Figure 1I–M). Together, these findings identify ECM organization as a prominently dysregulated program in GC and link ITGA11 expression to ECM-related activity.

### 3.2. ITGA11 Is Upregulated in Gastric Cancer, Associated with Poor Prognosis, and Linked to the FAK/PI3K/AKT/mTOR Axis

**Figure 2:**
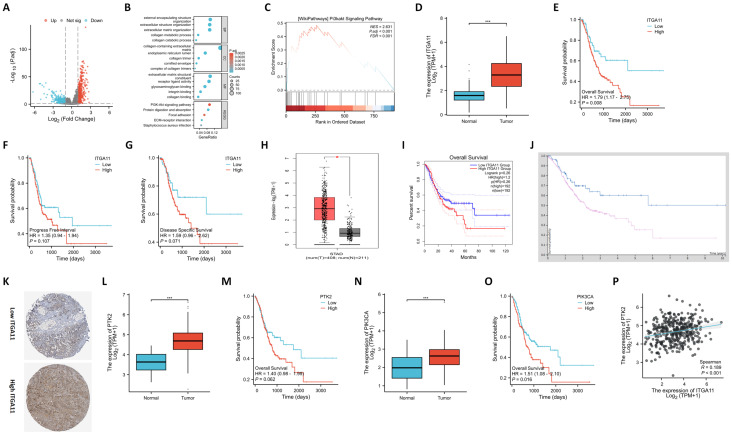
ITGA11 overexpression predicts poor prognosis and correlates with the FAK/PI3K/AKT/mTOR axis in GC. (**A**) Volcano plot of DEGs between ITGA11-high and ITGA11-low tumors. (**B**) GO and KEGG enrichment analyses of DEGs. (**C**) Gene Set Enrichment Analysis (GSEA) of DEGs. (**D**) ITGA11 expression in tumor versus normal tissues from TCGA-STAD cohort. (**E**–**G**) Kaplan–Meier curves for OS (**E**), PFI (**F**), and DSS (**G**) stratified by ITGA11 expression. (**H**) ITGA11 expression in tumor versus normal tissues from the GEPIA database. (**I**) Kaplan–Meier curves for OS stratified by ITGA11 expression from the GEPIA database. (**J**) Kaplan–Meier curves for OS stratified by ITGA11 expression from Human Protein Atlas. (**K**) Representative immunohistochemical images of ITGA11 from the Human Protein Atlas. (**L**) PTK2 expression in tumor versus normal tissues. (**M**) Kaplan–Meier curves for OS stratified by PTK2 expression. (**N**) PIK3CA expression in tumor versus normal tissues. (**O**) Kaplan–Meier curves for OS stratified by PIK3CA expression. (**P**) Correlation analyses between ITGA11 and PTK2 expression. A total of 32 normal samples and 375 tumor samples in TCGA-STAD cohort were analyzed. * *p* < 0.05, *** *p* < 0.001.

To further elucidate the biological relevance of ITGA11, we stratified the TCGA-STAD tumor cohort into ITGA11-high and ITGA11-low groups using the median ITGA11 expression level as the cutoff. Differential expression analysis identified 474 upregulated and 735 downregulated genes (|log2FC| ≥ 1, adjusted *p* < 0.05) (Figure 2A). Functional enrichment analysis of these DEGs revealed strong enrichment of extracellular matrix organization. Representative Gene Ontology categories included external encapsulating structure organization, collagen-containing extracellular matrix and extracellular matrix structural constituent (Figure 2B). In addition, both KEGG pathway analysis and Gene Set Enrichment Analysis (GSEA) indicated significant enrichment of the PI3K-Akt signaling pathway (Figure 2B,C), suggesting a potential association between ITGA11 expression and PI3K-Akt signaling. Further analysis revealed that ITGA11 mRNA expression was markedly increased in gastric tumor tissues compared with adjacent normal tissues (Figure 2D). Clinically, elevated ITGA11 expression was associated with worse survival outcomes, including reduced overall survival (Figure 2E), Progression Free Interval (Figure 2F), and Disease-Specific Survival (Figure 2G). In addition, we performed univariate and multivariate Cox regression analyses incorporating clinical characteristics from the TCGA-STAD cohort and ITGA11 expression levels. These analyses demonstrated that high ITGA11 expression was a potential prognostic risk factor for gastric cancer (Hazard ratio (95% CI): 1.639 (1.152–2.330, *p* = 0.006, Table S6)). To further validate differences in ITGA11 expression and its prognostic significance in gastric cancer, we also analyzed patient cohorts from the GEPIA database (http://gepia2.cancer-pku.cn/) and the Human Protein Atlas (https://www.proteinatlas.org/). The results consistently showed that ITGA11 is upregulated in gastric cancer and associated with poorer prognosis (Figure 2H–K). Given the enrichment of the PI3K-AKT pathway, we further analyzed key components of the FAK/PI3K/AKT/mTOR axis, as FAK is a well-established upstream regulator of PI3K signaling and an important mediator of integrin activity [37]. mRNA expression of core pathway constituents, such as PTK2 (FAK) (Figure 2L), SRC (Figure S2A), PIK3CA (Figure 2N), AKT1 (Figure S2B), RPS6KB1 (Figure S2C), EIF4EBP1 (Figure S2D), and MTOR (Figure S2E), was significantly higher in tumor tissues than in normal tissues. Among these genes, only high PIK3CA expression was significantly associated with poor OS (Figure 2O), whereas the remaining genes did not show significant prognostic associations (Figure 2M and Figure S2F–J). Correlation analysis further showed that ITGA11 expression was positively associated with PTK2, SRC, PIK3CA, AKT1, RPS6KB1, and MTOR, but not with EIF4EBP1 (Figure 2P and Figure S2K–P). In conclusion, these findings are consistent with ITGA11 upregulation in GC and its association with adverse clinical outcomes and further suggest that the FAK/PI3K/AKT/mTOR axis may represent a candidate downstream signaling pathway linked to ITGA11.

### 3.3. ITGA11 Promotes Gastric Cancer Progression In Vitro and Is Associated with Adverse Clinicopathological Features

**Figure 3:**
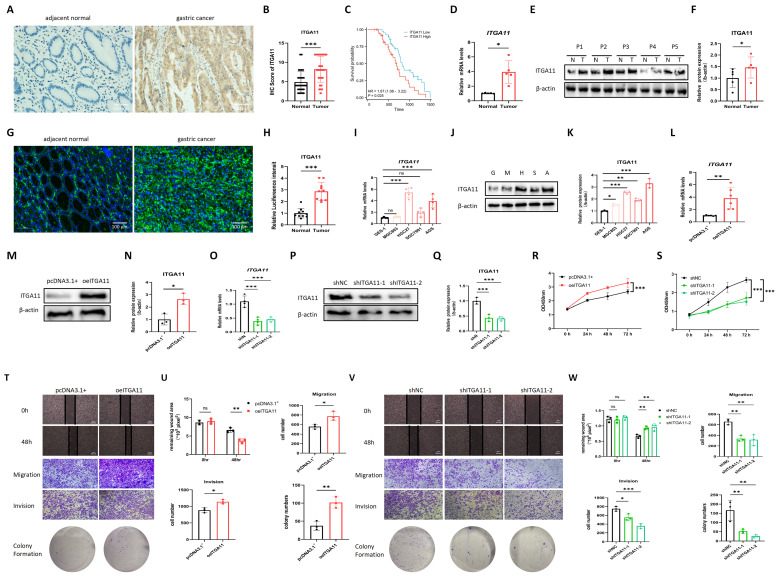
Upregulation of ITGA11 in gastric cancer predicts poor prognosis and promotes malignant progression in AGS cells. (**A**) Representative IHC staining of ITGA11 in paired GC and adjacent normal tissues (Stage II). (**B**) Quantification of IHC staining scores for ITGA11 from 60 paired GC and normal tissues (n = 60). (**C**) Kaplan–Meier survival analysis of overall survival in 60 GC patients stratified by ITGA11 expression level. (**D**–**F**) ITGA11 mRNA (**D**) and protein (**E**,**F**) expression in 5 pairs of GC and adjacent normal tissues (n = 5). (**G**) Representative IF staining of ITGA11 in 10 paired GC and adjacent normal tissues (Stage III). (**H**) Quantification of ITGA11 fluorescence intensity in 10 paired GC and normal tissues (n = 10). (**I**–**K**) ITGA11 mRNA (**I**) and protein (**J**,**K**) expression in a panel of GC cell lines (MGC-803, HGC-27, SGC-7901, AGS) relative to the normal gastric epithelial cell line GES-1 (n = 4 for panel (**I**), and n = 3 for panels (**J**,**K**)). (**L**–**N**) Gene (**L**) and protein (**M**,**N**) expression of ITGA11 following ITGA11 overexpression (oeITGA11) in AGS cells (n = 6 for panel (**L**), and n = 3 for panels (**M**,**N**)). (**O**–**Q**) Gene (**O**) and protein (**P**,**Q**) expression of ITGA11 following ITGA11 knockdown (shITGA11-1/2) in AGS cells (n = 4 for panel (**O**), and n = 3 for panels (**P**,**Q**)). (**R**,**S**) CCK-8 assay following ITGA11 overexpression (**R**) or knockdown (**S**) in AGS cells (n = 3). (**T**,**U**) Wound healing, transwell and colony formation assays following ITGA11 overexpression in AGS cells (n = 3). (**V**,**W**) Wound healing, transwell and colony formation assays following ITGA11 knockdown in AGS cells (n = 3). Scale bars: 100 μm for IHC, IF, transwell images and 500 μm for wound healing images. Data are presented as mean ± s.d. Two-group comparisons were performed using Student’s *t* test, and multiple groups were analyzed by one-way ANOVA. ns, not significant, * *p* < 0.05, ** *p* < 0.01, *** *p* < 0.001. The original Western blot figures can be found in Supplementary File S1.

To validate and extend the bioinformatic findings, we performed a series of clinical and functional experiments. Initially, IHC analysis of 60 paired patient samples showed that ITGA11 protein levels were significantly higher in GC tissues than in adjacent normal tissues (Figure 3A,B). Clinicopathological analysis of this cohort further demonstrated that high ITGA11 expression was associated with higher Ki-67 levels, poorer differentiation, and more advanced N stage (Table S1). Patients with high ITGA11 expression in our institutional cohort also had significantly worse overall survival (Figure 3C). To further evaluate the prognostic value of ITGA11, we performed univariate and multivariate Cox regression analyses using clinicopathological factors and ITGA11 expression levels in our cohort of 60 patients. The univariate Cox analysis suggested that ITGA11 expression was associated with prognosis in gastric cancer. Although ITGA11 did not reach statistical significance in the multivariate analysis, it showed a clear trend toward being an independent prognostic risk factor (hazard ratio [95% CI]: 2.024 [0.992–4.131], *p* = 0.053; Table S7). Together with the findings presented in Table S6, these results suggest that ITGA11 may serve as a candidate prognostic biomarker for gastric cancer. The upregulation of ITGA11 was further confirmed in five pairs of freshly resected samples, in which both qPCR (Figure 3D) and Western blot analyses (Figure 3E,F) showed increased ITGA11 expression in tumor tissues. Additionally, IF staining of an additional 10 paired samples revealed stronger ITGA11 signals in tumor tissues than in adjacent normal tissues (Figure 3G,H). Subsequently, we assessed ITGA11 expression in human GC cell lines, including MGC-803, HGC-27, SGC-7901, and AGS, relative to the immortalized normal gastric epithelial cell line GES-1. Both qPCR (Figure 3I) and Western blot analyses (Figure 3J,K) demonstrated significantly higher ITGA11 expression in all GC cell lines examined. Notably, HGC-27 and AGS cells exhibited the highest ITGA11 expression levels and were therefore selected for subsequent in vitro functional studies. To explore the functional implications of ITGA11, overexpression and knockdown models were established in AGS and HGC-27 cells. The efficiency of ITGA11 overexpression (oeITGA11) and knockdown (shITGA11-1/2) was confirmed at both the mRNA and protein levels in AGS cells (Figure 3L–N and Figure 3O–Q, respectively). CCK-8 assays showed that oeITGA11 significantly enhanced cell proliferation compared with the pcDNA3.1+ control (Figure 3R), whereas ITGA11 knockdown suppressed proliferation (Figure 3S). Moreover, ITGA11 overexpression significantly promoted wound healing, migration, invasion, and colony formation in AGS cells (Figure 3T,U), whereas ITGA11 knockdown produced the opposite effects (Figure 3V,W). Similar results were observed in HGC-27 cells (Figure S3A–L). Together, these findings suggest that ITGA11 is upregulated in GC and promotes malignant phenotypes in GC cells.

### 3.4. FAK Is Activated Downstream of ITGA11 and Promotes Gastric Cancer Progression

**Figure 4:**
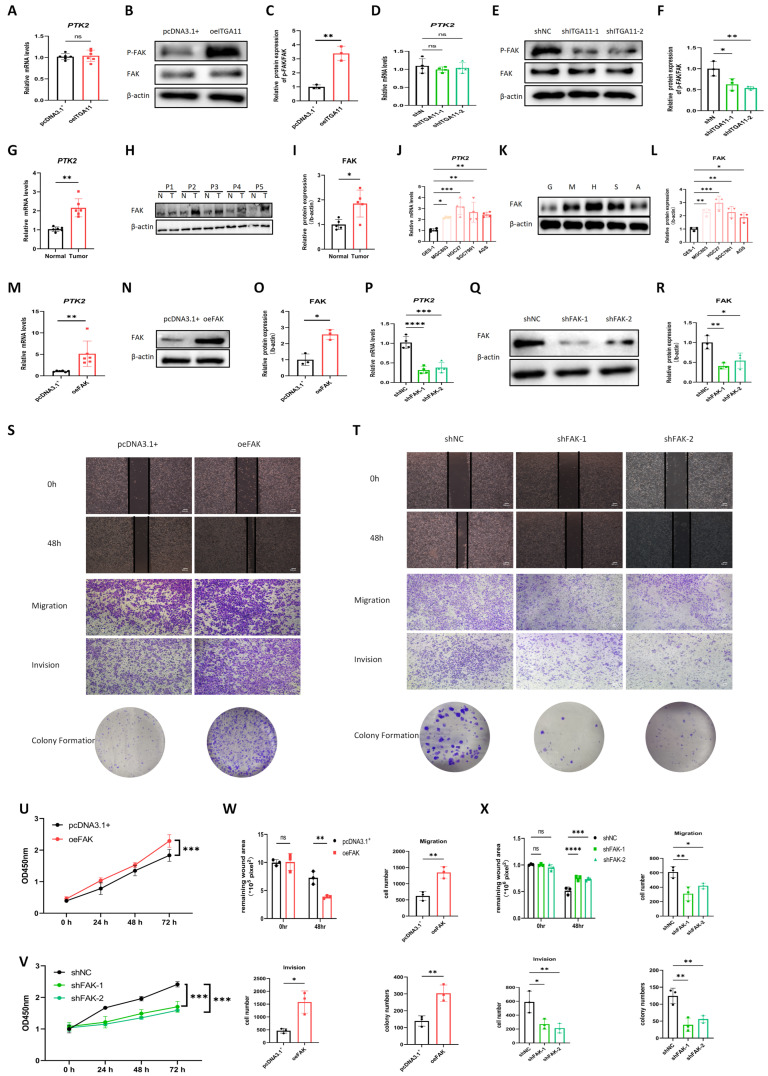
ITGA11-regulated FAK is upregulated in GC and promotes malignant progression in AGS cells. (**A**–**C**) Gene (**A**) and protein (**B**,**C**) expression of FAK(PTK2)/p-FAK following ITGA11 overexpression in AGS cells (n = 6 for panel (**A**), and n = 3 for panels (**B**,**C**)). (**D**–**F**) Gene (**D**) and protein (**E**,**F**) expression of FAK(PTK2)/p-FAK following ITGA11 knockdown in AGS cells (n = 4 for panel (**D**), and n = 3 for panels (**E**,**F**)). (**G**–**I**) FAK(PTK2) mRNA (**G**) and protein (**H**,**I**) expression in 5 pairs of GC and adjacent normal tissues (n = 5). (**J**–**L**) FAK(PTK2) mRNA (**J**) and protein (**K**,**L**) expression in a panel of GC cell lines (MGC-803, HGC-27, SGC-7901, AGS) relative to GES-1 (n = 4 for panel (**J**), and n = 3 for panels (**K**,**L**)). (**M**–**O**) Gene (**M**) and protein (**N**,**O**) expression of FAK(PTK2) with FAK overexpression (oeFAK) in AGS cells (n = 6 for panel (**M**), and n = 3 for panels (**N**,**O**)). (**P**–**R**) Gene (**P**) and protein (**Q**,**R**) expression of FAK(PTK2) with FAK knockdown (shFAK-1/2) in AGS cells (n = 4 for panel (**P**), and n = 3 for panels (**Q**,**R**)). (**S**,**T**) Representative images of wound healing, transwell and colony formation assays with FAK overexpression (**S**) and knockdown (**T**) in AGS cells. (**U**,**V**) CCK-8 assay with FAK overexpression (**U**) and knockdown (**V**) in AGS cells. (**W**,**X**) Quantification of functional assays with FAK overexpression (**W**) and knockdown (**X**) in AGS cells (n = 3). Scale bars: 100 μm for transwell images and 500 μm for wound healing images. Data are presented as mean ± s.d. Two-group comparisons were performed using Student’s *t* test, and multiple groups were analyzed by one-way ANOVA. ns, not significant, * *p* < 0.05, ** *p* < 0.01, *** *p* < 0.001, **** *p* < 0.0001. The original Western blot figures can be found in Supplementary File S1.

After establishing the clinical significance and pro-tumorigenic functions of ITGA11 in GC, we next sought to investigate the underlying mechanisms. Given the potential association between ITGA11 and the PI3K-AKT axis, we hypothesized that ITGA11 promotes tumor progression through FAK, an established upstream regulator of PI3K signaling [38]. To test this hypothesis, we initially examined whether ITGA11 influences FAK expression and activation. qPCR and Western blot analyses demonstrated that ITGA11 overexpression significantly increased FAK phosphorylation without affecting total FAK mRNA or protein levels (Figure 4A–C). In contrast, ITGA11 knockdown reduced phosphorylated FAK levels (Figure 4D–F), suggesting that ITGA11 regulates FAK activation in GC cells. We next investigated FAK expression and function in GC. Consistent with the bioinformatic analysis (Figure 2L), qPCR and Western blot analyses demonstrated a significant upregulation of FAK mRNA and protein levels in five paired GC tissue samples compared to adjacent normal tissues (Figure 4G–I). Elevated FAK expression was also observed in GC cell lines, including MGC-803, HGC-27, SGC-7901, and AGS, relative to GES-1 cells (Figure 4J–L). To further define the functional role of FAK, we established FAK overexpression and knockdown models in AGS cells, and the efficiency of these models was confirmed by qPCR and Western blot analyses (Figure 4M–R). Functional assays revealed that FAK overexpression significantly promoted proliferation, wound healing, migration, invasion, and colony formation (Figure 4S,U,W) in AGS cells, whereas FAK knockdown produced the opposite effects (Figure 4T,V,X). Similar results were observed in HGC-27 cells (Figure S4A–R). Together, these findings suggest that ITGA11 promotes GC progression, at least in part, through FAK activation, which may also facilitate GC progression.

### 3.5. ITGA11 Promotes GC Progression by Activating the FAK/PI3K/AKT/mTOR Axis

**Figure 5:**
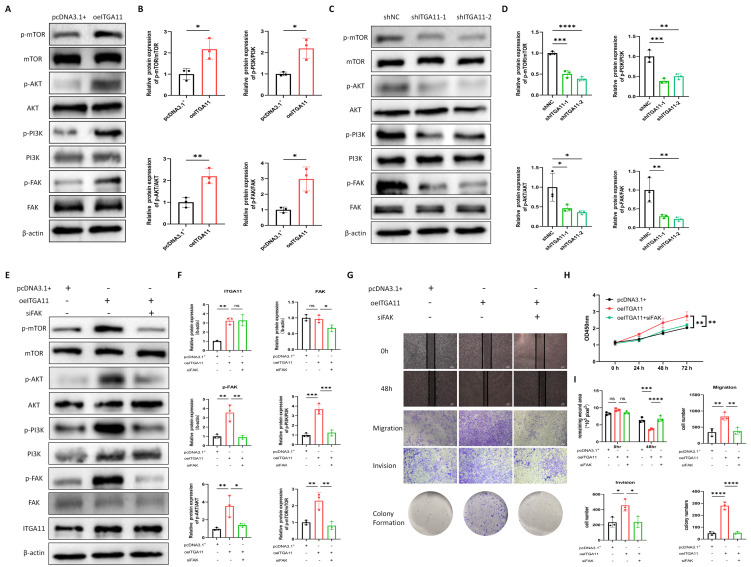
ITGA11 activates the FAK/PI3K/AKT/mTOR axis in AGS cells. (**A**) Representative immunoblot images of the FAK/PI3K/AKT/mTOR axis following ITGA11 overexpression in AGS cells. (**B**) Quantification of the FAK/PI3K/AKT/mTOR axis following ITGA11 overexpression in AGS cells. (**C**) Representative immunoblot images of the FAK/PI3K/AKT/mTOR axis following ITGA11 knockdown in AGS cells. (**D**) Quantification of the FAK/PI3K/AKT/mTOR axis following ITGA11 knockdown in AGS cells. (**E**) Representative immunoblot images of ITGA11 and the FAK/PI3K/AKT/mTOR axis following ITGA11 overexpression and FAK knockdown in AGS cells. (**F**) Quantification of ITGA11 and the FAK/PI3K/AKT/mTOR axis following ITGA11 overexpression and FAK knockdown in AGS cells. (**G**) Representative images of wound healing, transwell and colony formation assays following ITGA11 overexpression and FAK knockdown in AGS cells. (**H**) CCK-8 assay following ITGA11 overexpression and FAK knockdown in AGS cells. (**I**) Quantification of functional assays following ITGA11 overexpression and FAK knockdown in AGS cells. Scale bars: 100 μm for transwell images and 500 μm for wound healing images. Data are presented as mean ± s.d. (n = 3 per group). Two-group comparisons were performed using Student’s *t* test, and multiple groups were analyzed by one-way ANOVA. ns, not significant, * *p* < 0.05, ** *p* < 0.01, *** *p* < 0.001, **** *p* < 0.0001. The original Western blot figures can be found in Supplementary File S1.

**Figure 6:**
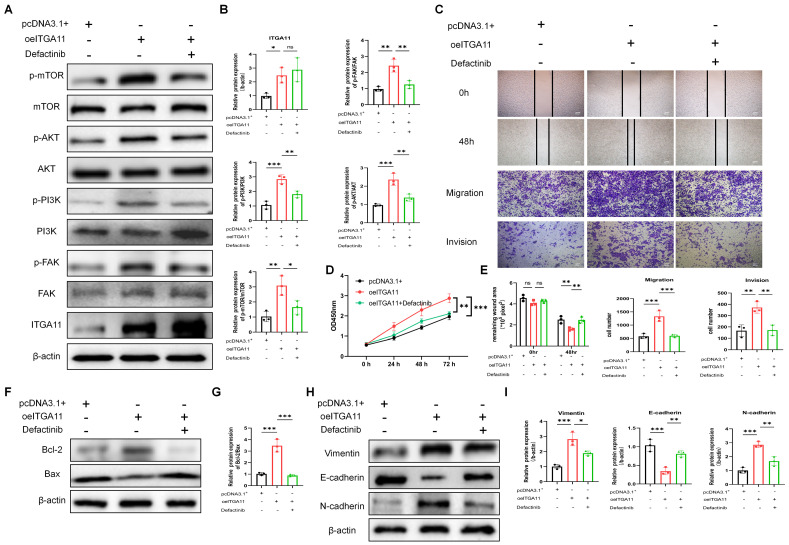
Defactinib attenuates ITGA11-induced malignant phenotypes by inhibiting the FAK/PI3K/AKT/mTOR signaling axis. (**A**) Representative immunoblot images of ITGA11 and the FAK/PI3K/AKT/mTOR axis following ITGA11 overexpression and Defactinib treatment (10 μM). (**B**) Quantification of ITGA11 and the FAK/PI3K/AKT/mTOR axis following ITGA11 overexpression and Defactinib treatment (10 μM). (**C**) Representative images of wound healing and transwell assays following ITGA11 overexpression and Defactinib treatment (10 μM). (**D**) CCK-8 assay following ITGA11 overexpression and Defactinib treatment (10 μM). (**E**) Quantification of functional assays following ITGA11 overexpression and Defactinib treatment (10 μM). (**F**) Representative immunoblot images of Bax and Bcl-2 following ITGA11 overexpression and Defactinib treatment (10 μM). (**G**) Quantification of Bax and Bcl-2 following ITGA11 overexpression and Defactinib treatment (10 μM). (**H**) Representative immunoblot images of Vimentin, E-cadherin, and N-cadherin following ITGA11 overexpression and Defactinib treatment (10 μM). (**I**) Quantification of Vimentin, E-cadherin, and N-cadherin following ITGA11 overexpression and Defactinib treatment (10 μM). Scale bars: 100 μm for transwell images and 500 μm for wound healing images. Data are presented as mean ± s.d. (n = 3 per group). Multiple groups were analyzed by a one-way ANOVA. ns, not significant, * *p* < 0.05, ** *p* < 0.01, *** *p* < 0.001. The original Western blot figures can be found in Supplementary File S1.

Given the established role of this pathway in GC progression, we next examined whether ITGA11 regulates the FAK/PI3K/AKT/mTOR axis in GC cells. In AGS cells, ITGA11 overexpression led to a significant increase in the phosphorylation of FAK, PI3K, AKT, and mTOR, while the total protein levels of these molecules remained unchanged (Figure 5A,B). Conversely, knockdown of ITGA11 in AGS cells resulted in a marked reduction in the phosphorylation of FAK, PI3K, AKT, and mTOR, without altering their total protein levels (Figure 5C,D). These findings support the role of ITGA11 in regulating the FAK/PI3K/AKT/mTOR signaling pathway. To further assess whether this pathway mediates ITGA11-associated malignant phenotypes, we assessed the effects of FAK silencing in ITGA11-overexpressing GC cells. Rescue experiments demonstrated that transfection with FAK-targeting siRNA effectively reduced FAK expression in AGS cells, while ITGA11 levels remained unchanged (Figure 5E,F). Importantly, FAK knockdown significantly attenuated ITGA11-induced phosphorylation of FAK, PI3K, AKT, and mTOR, without affecting total PI3K, AKT, or mTOR protein levels; as expected, total FAK levels were reduced by FAK silencing (Figure 5E,F). These molecular changes were accompanied by a marked reversal of ITGA11-induced malignant phenotypes. Functional assays revealed that FAK silencing significantly reduced proliferation, migration, invasion, and colony formation of ITGA11-overexpressing AGS cells (Figure 5G–I). Similar results were observed in HGC-27 cells (Figure S5A–I), further supporting the involvement of the FAK/PI3K/AKT/mTOR axis in ITGA11-mediated malignancy. To further investigate the regulation of FAK/PI3K/AKT/mTOR signaling by ITGA11, we treated ITGA11-overexpressing HGC-27 cells with the FAK inhibitor Defactinib (10 μM) to assess the pathway’s dependency on FAK activity. Defactinib treatment markedly suppressed ITGA11-induced phosphorylation of FAK, PI3K, AKT, and mTOR, without affecting their total protein levels (Figure 6A,B). Consistently, functional assays demonstrated that Defactinib significantly inhibited ITGA11-mediated proliferation, migration, and invasion of HGC-27 cells (Figure 6C–E). These results further highlight that the biological effects of ITGA11 in gastric cancer cells are largely mediated by FAK activation and its downstream signaling cascade. Considering that FAK/PI3K/AKT/mTOR signaling regulates apoptosis and EMT, we further investigated whether ITGA11 overexpression and Defactinib treatment modulate these biological processes in gastric cancer cells. Our results suggest that ITGA11 overexpression inhibited apoptosis in HGC-27 cells, as shown by an increased Bcl-2/Bax ratio (Figure 6F,G), and promoted EMT, evidenced by decreased E-cadherin and increased N-cadherin and Vimentin expression (Figure 6H–I). Importantly, treatment with Defactinib significantly attenuated these effects, suggesting that ITGA11 regulates apoptosis and EMT at least in part through the FAK/PI3K/AKT/mTOR signaling axis. Together, these findings suggest that ITGA11 promotes GC malignancy by activating the FAK/PI3K/AKT/mTOR signaling axis, with FAK serving as an important mediator of this process.

### 3.6. Naringenin Functionally Inhibits ITGA11-Associated FAK/PI3K/AKT/mTOR Signaling and Suppresses Malignant Phenotypes in GC Cells

**Figure 7:**
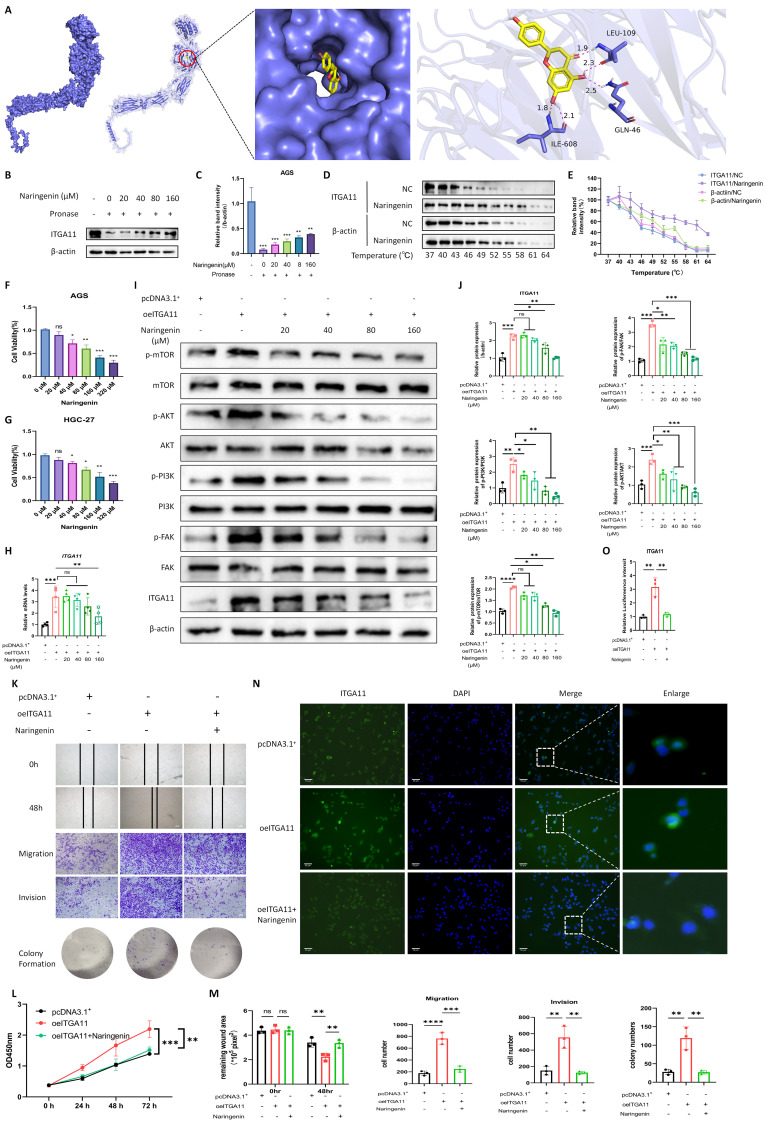
Naringenin suppresses ITGA11-associated FAK/PI3K/AKT/mTOR signaling and malignant phenotypes in GC cells. (**A**) Molecular docking binding mode between Naringenin and ITGA11. (**B**,**C**) DARTS assays and quantitative analysis of Naringenin and ITGA11 (n = 3). (**D**,**E**) CETSAs and quantitative analysis of Naringenin and ITGA11 (n = 3). (**F**,**G**) Cell viability assay of Naringenin in AGS (**F**) and HGC-27 (**G**) cells for 24 h (n = 3). (**H**) Gene expression of ITGA11 following ITGA11 overexpression and Naringenin treatment (20, 40, 80, and 160 µM for 24 h) in AGS cells (n = 4). (**I**) Representative immunoblot images of ITGA11 and the FAK/PI3K/AKT/mTOR axis following ITGA11 overexpression and Naringenin treatment (20, 40, 80, and 160 µM for 24 h) in AGS cells. (**J**) Quantification of ITGA11 and FAK/PI3K/AKT/mTOR axis following ITGA11 overexpression and Naringenin treatment (20, 40, 80, and 160 µM for 24 h) in AGS cells (n = 3). (**K**) Representative images of wound healing, transwell and colony formation assays following ITGA11 overexpression and Naringenin treatment (40 µM; 24 h) in AGS cells. (**L**) CCK-8 assay following ITGA11 overexpression and Naringenin treatment (40 µM; 24 h) in AGS cells. (**M**) Quantification of functional assays following ITGA11 overexpression and Naringenin treatment (40 µM; 24 h) in AGS cells (n = 3). (**N**,**O**) IF staining of ITGA11 following ITGA11 overexpression and Naringenin treatment (40 µM; 24 h) in HGC-27 cells (n = 3). Scale bars: 50 μm for IF images, 100 μm for transwell images and 500 μm for wound healing images. Data are presented as mean ± s.d. Multiple groups were analyzed by one-way ANOVA. ns, not significant, * *p* < 0.05, ** *p* < 0.01, *** *p* < 0.001, **** *p* < 0.0001. The original Western blot figures can be found in Supplementary File S1.

Given the oncogenic role of ITGA11 in GC, we next sought to explore potential therapeutic strategies targeting ITGA11-associated signaling. Virtual screening identified Naringenin as a top-ranked candidate, which was then subjected to detailed molecular docking analysis against ITGA11 (Table S8). Naringenin, a major flavanone derived from citrus fruits, has attracted considerable attention due to its anti-inflammatory, antioxidant, and broad-spectrum antitumor activities. Although increasing evidence has suggested its beneficial effects in multiple cancer types, the role of Naringenin in gastric cancer remains largely unclear, especially regarding whether its antitumor activity is mediated through regulation of ITGA11-associated signaling [30,31,32]. In this study, molecular docking analysis predicted a potential binding mode between Naringenin and ITGA11, supporting its subsequent experimental evaluation (Figure 7A). Subsequently, we employed complementary target identification approaches to investigate the potential interaction between Naringenin and ITGA11, and the functional consequences of this binding. Using DARTS analysis in AGS cell lysates, immunoblotting revealed that ITGA11 protein levels were progressively preserved following protease digestion at increasing concentrations of Naringenin, suggesting that Naringenin could interact with ITGA11 and confers protease resistance (Figure 7B,C). Consistently, CETSA experiments demonstrated that ITGA11 exhibited markedly higher thermal stability in Naringenin-treated AGS cells across a range of temperatures compared with control cells (Figure 7D,E). Together, these results provide strong evidence that Naringenin interacts with ITGA11 and modulates its structural stability. Next, we assessed the effects of Naringenin over a range of concentrations and selected 40 µM as the working concentration for subsequent functional assays in both AGS (Figure 7F) and HGC-27 cells (Figure 7G). We then examined the effects of Naringenin on ITGA11 and downstream signaling in ITGA11-overexpressing AGS cells using a dose-gradient design (20, 40, 80, and 160 µM for 24 h). Naringenin had little effect on ITGA11 mRNA or protein expression at lower concentrations, whereas a reduction in ITGA11 mRNA and protein levels became evident only at higher concentrations (Figure 7H–J). However, Western blot analysis showed that phosphorylation of FAK, PI3K, AKT, and mTOR was progressively suppressed in a dose-dependent manner, while the total protein levels of FAK, PI3K, AKT, and mTOR remained largely unchanged (Figure 7I,J). These findings suggest that Naringenin attenuates ITGA11-associated signaling in a dose-dependent manner. The expression of ITGA11 is significantly inhibited only at higher concentrations, suggesting that Naringenin may exert both functional inhibition and expression suppression when binding to ITGA11. At the selected concentration of 40 µM, Naringenin significantly reduced proliferation, migration, invasion, and colony formation in ITGA11-overexpressing AGS cells (Figure 7K–M). Similar results were observed in HGC-27 cells (Figure S6A–D). In addition, IF analysis in HGC-27 cells showed that treatment with 40 µM Naringenin for 24 h reduced ITGA11 fluorescence intensity (Figure 7N,O). Next, we investigated whether the inhibitory effects of Naringenin are dependent on ITGA11. Both qPCR and Western blot analyses showed that treatment of ITGA11-knockdown HGC-27 cells with Naringenin (40 µM) resulted in a modest, non-significant reduction in ITGA11 expression (Figure S6E,F). Consistently, functional assays demonstrated that the proliferation, migration, and invasion of ITGA11-depleted HGC-27 cells were not further suppressed by Naringenin treatment, indicating that the inhibitory effects of Naringenin are largely ITGA11-dependent (Figure S6G,H). Together, these findings suggest that Naringenin may functionally attenuate ITGA11-associated FAK/PI3K/AKT/mTOR signaling and related malignant phenotypes in GC cells.

### 3.7. Naringenin Suppresses ITGA11-Associated Tumor Growth In Vivo

**Figure 8:**
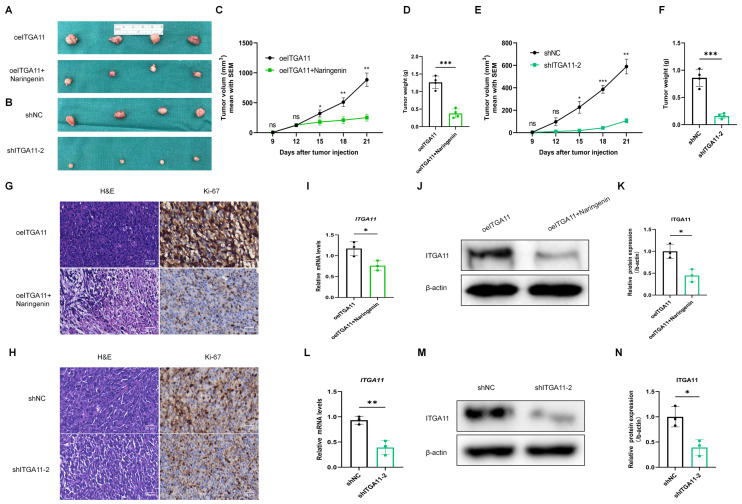
Targeting ITGA11 suppresses gastric cancer progression in vivo. (**A**) Subcutaneous tumors of ITGA11 overexpression and Naringenin treatment (n = 4). (**B**) Subcutaneous tumors of ITGA11 knockdown (n = 4). (**C**,**D**) Tumor volume (**C**) and weight (**D**) of ITGA11 overexpression and Naringenin treatment (n = 4). (**E**,**F**) Tumor volume (**E**) and weight (**F**) of ITGA11 knockdown (n = 4). (**G**,**H**) Representative images of H&E and Ki-67 staining for subcutaneous tumors following ITGA11 overexpression and Naringenin treatment (**G**) and ITGA11 knockdown (**H**). (**I**–**K**) Gene (**I**) and protein (**J**,**K**) expression of ITGA11 following ITGA11 overexpression and Naringenin treatment in subcutaneous tumors. (**L**–**N**) Gene (**L**) and protein (**M**,**N**) expression of ITGA11 following ITGA11 knockdown in subcutaneous tumors (n = 3). Scale bars: 50 μm for H&E and Ki-67 staining. Data are presented as mean ± SEM for tumor volume curves and mean ± s.d. for other analyses. Two-group comparisons were performed using Student’s *t* test, and panels (**C**,**E**) were performed using repeated-measures ANOVA. ns, not significant, * *p* < 0.05, ** *p* < 0.01, *** *p* < 0.001 The original Western blot figures can be found in Supplementary File S1.

To further elucidate the oncogenic role of ITGA11 and evaluate the therapeutic effect of Naringenin in vivo, we established subcutaneous xenograft tumor models using AGS cells with ITGA11 overexpression or knockdown. ITGA11 overexpression significantly promoted tumor growth, as evidenced by increased tumor volume and weight (Figure 8A,C,D). Intraperitoneal administration of Naringenin markedly attenuated the tumor-promoting effect of ITGA11 overexpression, resulting in significantly reduced tumor growth compared with the untreated ITGA11-overexpressing group (Figure 8A,C,D). Consistently, tumors derived from ITGA11-silenced cells exhibited significantly lower tumor volumes and final tumor weights than those in the shNC group (Figure 8B,E,F). These findings support a potential tumor-promoting role of ITGA11 in GC in vivo and suggest that Naringenin effectively attenuates ITGA11-associated tumor growth. More detailed animal experiment data are exhibited in Table S9. Throughout the whole in vivo experiment, we did not observe any additional drug toxicity or intolerance at the administered dose of Naringenin. We subsequently performed histological examination and Ki-67 staining of the xenograft tumor tissues. These analyses were conducted by experienced pathologists who were blinded to the group allocation. Our results further confirmed that tumors in the ITGA11-overexpression group exhibited accelerated growth and a higher Ki-67 index. In contrast, Naringenin treatment or ITGA11 knockdown reduced the Ki-67 index and proliferative activity of the tumors. Representative H&E and Ki-67 staining images randomly selected from each group are shown in Figure 8G,H. qPCR and Western blot analyses of xenograft tissues further confirmed altered ITGA11 expression in vivo (Figure 8I–N). In conclusion, these in vivo results support the role of ITGA11 in promoting GC tumor growth and suggest that Naringenin could suppress ITGA11-associated tumor progression in vivo.

## 4. Discussion

Gastric cancer remains a major clinical challenge, with metastatic dissemination accounting for most disease-related deaths [1]. Although the tumor microenvironment, particularly the remodeled extracellular matrix (ECM), plays a critical role in cancer progression, the molecular basis by which gastric cancer cells perceive and transduce ECM-derived cues remains incompletely defined [39]. In this study, we identify ITGA11 as a clinically relevant mediator of ECM-associated gastric cancer progression and provide evidence that its tumor-promoting effects are associated with activation of the FAK/PI3K/AKT/mTOR signaling cascade. Notably, bioactive compounds derived from edible plants have attracted increasing attention in medical research, particularly as potential anticancer agents [40]. In this context, our findings further suggest that ITGA11-associated signaling may represent a targetable pathway in GC, as suggested by the inhibitory effects of Naringenin in our experimental models.

Our study demonstrates that ITGA11 is consistently upregulated in GC tissues and that this upregulation is associated with unfavorable clinicopathological characteristics and reduced patient survival. These observations are consistent with and extend previous reports implicating ITGA11 in the progression of other solid tumors, such as breast, lung, and pancreatic cancers [27,41,42,43,44]. Nonetheless, our study was limited to 60 pathological specimens and 5 paired fresh gastric cancer tissues for validating ITGA11 expression. To mitigate potential sampling bias and enhance the evidential strength of our conclusions, future studies incorporating larger patient cohorts, particularly with more paired fresh tissues, will be essential. The positive correlation between ITGA11 and ECM-related signatures, as well as with collagen-associated genes such as COL1A1, COL5A2, and LOX, is particularly relevant in GC, where stromal stiffening and collagen accumulation are prominent features of the tumor microenvironment [45]. In this setting, ITGA11 is likely to function not merely as a marker of matrix remodeling, but as a collagen-responsive signaling node that enables tumor cells to convert ECM-derived mechanical and biochemical cues into intracellular pro-tumorigenic signaling. This interpretation is further supported by our gain- and loss-of-function experiments, which showed that ITGA11 enhances proliferation, migration, invasion, and clonogenic growth in GC cells.

Mechanistically, our data support a model in which ITGA11 promotes GC progression, at least in part by activating FAK and its downstream PI3K/AKT/mTOR signaling axis. FAK is a key downstream effector in integrin-mediated signaling and is well known to couple cell-ECM adhesion to survival, motility, and growth-related pathways [46,47,48]. In our study, ITGA11 altered the phosphorylation state of FAK without changing total FAK abundance, indicating that ITGA11 primarily regulates this pathway at the level of kinase activation rather than protein expression. The rescue experiments further support this conclusion, as FAK silencing or pharmacological inhibition markedly attenuated ITGA11-induced pathway activation and malignant phenotypes [49]. The subsequent activation of the PI3K/AKT/mTOR axis, a well-established pathway that regulates cell proliferation, survival, and metabolism, provides a mechanistic link between ECM sensing and fundamental oncogenic processes [50,51,52]. This is consistent with a previous study showing that ITGA11 influenced gastric cancer progression by regulating the PI3K/AKT pathway. However, our study further elucidates FAK’s functional role in mediating the ITGA11 and PI3K/AKT/mTOR signaling axis. More importantly, we explored a potential candidate compound targeting ITGA11, Naringenin, which may facilitate the clinical translation of ITGA11-based interventions [53]. These findings are consistent with previous studies showing that integrin engagement induces FAK autophosphorylation, thereby creating docking sites for Src family kinases and adaptor proteins and propagating signals to PI3K and its downstream effectors [49,54,55]. Our study further demonstrated that ITGA11 suppresses apoptosis and promotes EMT in gastric cancer cells via activation of the FAK/PI3K/AKT/mTOR signaling pathway, and these effects can be effectively abrogated by the FAK inhibitor Defactinib. These results suggest that the biological functions of ITGA11 in gastric cancer cells are at least partially dependent on the FAK/PI3K/AKT/mTOR axis. At the same time, integrin signaling in cancer is intrinsically complex and can also engage additional pathways, including MAPK, YAP/TAZ, and TGF-β signaling programs, depending on receptor context, ligand availability, and matrix mechanics [56,57,58]. This suggests that future studies should investigate additional mechanisms by which ITGA11 regulates these phenotypes to identify novel therapeutic targets. Thus, the FAK/PI3K/AKT/mTOR axis appears to be a major mechanism underlying ITGA11-associated malignancy in our GC models, although it is unlikely to be the only one.

The translational significance of this study is underscored by the identification of Naringenin as a potential suppressor of ITGA11-associated signaling. Naringenin, a food-derived flavanone predominantly present in citrus fruits, adds to the accumulating evidence that plant-derived bioactive compounds may exert therapeutic effects against gastric cancer. This study further highlights the potential of food-derived compounds as a source of candidate anticancer agents. However, Naringenin is not an ITGA11-specific inhibitor, but rather a naturally occurring compound with pleiotropic anti-inflammatory, antioxidant, and antitumor activities, indicating that its molecular targets are unlikely to be restricted to ITGA11 alone [31,32,59]. While DARTS and CETSAs provide strong evidence that Naringenin interacts with ITGA11, these methods are indirect and do not quantify binding affinity. Therefore, future studies using SPR or ITC are warranted to precisely determine the binding kinetics and thermodynamics of this interaction, thereby further strengthening the mechanistic understanding of Naringenin’s effect on ITGA11 [60]. Nevertheless, our findings identify ITGA11 as a key potential target through which Naringenin exerts its effects in gastric cancer, as Naringenin effectively attenuates ITGA11-associated FAK/PI3K/AKT/mTOR signaling and reverses the malignant phenotypes enhanced by ITGA11 overexpression. Furthermore, in gastric cancer cells with ITGA11 knockdown, the inhibitory effects of Naringenin were markedly attenuated, supporting an ITGA11-dependent mechanism of action. Nonetheless, it should be noted that Naringenin may also exert effects through additional targets. Therefore, these findings only support the interpretation that Naringenin exerts antitumor effects, at least in part, by suppressing ITGA11-associated oncogenic signaling in GC. The anticancer effects of Naringenin have been demonstrated in various tumor models [31,32,33,61,62]. A completed phase I clinical study evaluating a citrus-derived Naringenin extract reported safety and pharmacokinetic assessment in humans, supporting its tolerability, although antitumor therapeutic efficacy was not evaluated in that setting [63]. In addition, recent evidence has shown that combining Naringenin with other anticancer agents, including paclitaxel or doxorubicin, can enhance its antitumor activity [64,65]. These findings highlight the complexity of tumor treatment and underscore the need for further research and clinical translation, particularly considering the limited efficacy of current targeted therapies for advanced gastric cancer (GC). Modulation of ITGA11 with Naringenin, in combination with other targeted drugs, may represent a potential approach for future development. Our study demonstrates that Naringenin suppresses ITGA11-associated signaling, thereby attenuating the FAK/PI3K/AKT/mTOR signaling pathway and reversing the malignant phenotype enhanced by ITGA11.

Our data reveal an additional mechanistic feature: Naringenin exerts both functional inhibition and expression suppression on ITGA11. At lower concentrations, Naringenin produced little change in ITGA11 mRNA expression and total protein abundance, yet it already suppressed activation of the FAK/PI3K/AKT/mTOR pathway. This suggests that early pathway inhibition may primarily result from Naringenin’s functional suppression of ITGA11 as an integrin receptor. Our rescue experiments further demonstrate that Naringenin’s effect on ITGA11 is ITGA11-dependent. At higher concentrations, however, Naringenin also reduced ITGA11 mRNA and protein levels, raising the possibility that its activity extends beyond receptor antagonism and may also affect ITGA11 expression itself. One plausible explanation is that disruption of integrin signaling feeds back into transcriptional and post-transcriptional programs that sustain ITGA11 expression. In this regard, previous studies have shown that ITGA11 can be regulated by TGF-β-dependent transcriptional mechanisms involving Smad proteins and Sp1 [66,67,68]. Previous studies have also shown that Naringenin can modulate TGF-β/Smad signaling pathways [69]. Therefore, it is plausible that higher concentrations of Naringenin may reduce ITGA11 expression by interfering with integrin-dependent signaling loops that converge on TGF-β/Smad or related mechanoresponsive transcriptional programs. Currently, however, this remains a hypothesis in GC, and the precise molecular mechanisms by which Naringenin influences ITGA11 expression require direct experimental investigation.

Several limitations and future research directions of this study should be acknowledged. Although our in vivo data support that Naringenin can inhibit the tumor-promoting effects of ITGA11, the limited number of mice, the single-dosing regimen, and the use of male mice in our study constrain the translational potential of Naringenin. Future studies should employ larger cohorts to evaluate multiple dosages and administration routes and investigate the bioavailability, toxicity, and tolerability of Naringenin in vivo. In addition, the use of orthotopic tumor models and patient-derived xenografts will be important for providing more clinically relevant insights and better informing their potential therapeutic applications. Second, while our docking, CETSA, and DARTS assays reveal an interaction between Naringenin and ITGA11, we did not directly verify physical binding between them. Therefore, additional target-engagement studies, including surface plasmon resonance (SPR) and isothermal titration calorimetry (ITC), will be necessary to confirm this interaction. Third, the mechanism by which high-dose Naringenin downregulates ITGA11 expression remains undefined and warrants focused mechanistic study. Finally, given the molecular heterogeneity of gastric cancer, future studies with larger, multicenter cohorts will be essential to further investigate ITGA11 expression patterns, prognostic significance, and potential differences across GC subtypes. Such studies will also clarify whether ITGA11-directed strategies are particularly relevant in ECM-rich or desmoplastic tumors [3].

## 5. Conclusions

In conclusion, our study identifies ITGA11 as a clinically relevant mediator of ECM-associated gastric cancer progression and supports the FAK/PI3K/AKT/mTOR pathway as a major downstream signaling axis. We further show that Naringenin, a food-derived flavanone, may suppress ITGA11-associated signaling and malignant phenotypes in GC models. These findings improve our understanding of extracellular matrix-associated tumor progression and support further investigation of food- and plant-derived bioactive compounds, particularly Naringenin, as potential candidates for modulating ITGA11-associated signaling in GC.

## Data Availability

The original contributions presented in the study are included in the article; the RNA-seq data used in this study were obtained from the TCGA-STAD cohort. Processed data, analysis scripts, and other supporting data can be obtained from the corresponding authors.

## Supplementary Materials

The following supporting information can be downloaded at: https://www.mdpi.com/article/10.3390/cancers18111712/s1, File S1: Original Images for Blots; Figure S1: Schematic workflow of the bioinformatic analysis and complementary ITGA11-related bioinformatic analyses. (A) Schematic workflow of the bioinformatic analysis. (B) PPI network of key genes in the ECM organization pathway. (C) ECM score in ITGA11-high versus ITGA11-low (All cohort). (D) Correlation between ITGA11 expression and ECM score (All cohort). (E) ECM ssGSEA score in ITGA11-high versus ITGA11-low (All cohort). (F) Correlation between ITGA11 expression and ECM ssGSEA score (All cohort). Data are presented as mean ± s.d. A total of 32 normal samples and 375 tumor samples in the TCGA-STAD cohort were analyzed; Figure S2: Bioinformatic analysis of FAK/PI3K/AKT/mTOR axis. (A–E) Gene expression of SRC (A), AKT1 (B), RPS6KB1 (C), EIF4EBP1 (D), MTOR (E) in tumor versus normal tissues. (F–J) Kaplan-Meier curves for OS stratified by gene expression of SRC (F), AKT1 (G), RPS6KB1 (H), EIF4EBP1 (I), MTOR (J). (K–P) Correlation analyses between ITGA11 and gene expression of SRC (K), PIK3CA (L), AKT1 (M), RPS6KB1 (N), EIF4EBP1 (O), MTOR (P). Data are presented as mean ± s.d. A total of 32 normal samples and 375 tumor samples in TCGA-STAD cohort were analyzed. ** *p* < 0.01, *** *p* < 0.001; Figure S3: ITGA11 promotes proliferation, migration, invasion, and colony formation in HGC-27 cells. (A–C) Gene (A) and protein (B,C) expression of ITGA11 following ITGA11 overexpression (oeITGA11) in HGC-27 cells (n = 6 for panel (A), and n = 3 for panels (B,C)). (D) CCK-8 assay following ITGA11 overexpression in HGC-27 cells (n = 3). (E–G) Gene (E) and protein (F,G) expression of ITGA11 following ITGA11 knockdown (shITGA11-1/2) in HGC-27 cells (n = 4 for panel (E), and n = 3 for panels (F,G)). (H) CCK-8 assay following ITGA11 knockdown in HGC-27 cells (n = 3). (I,J) Representative images of wound healing, transwell and colony formation assays following ITGA11 overexpression (I) and knockdown (J) in HGC-27 cells. (K,L) Quantification of functional assays following ITGA11 overexpression (K) and knockdown (L) in HGC-27 cells (n = 3). Scale bars: 100 μm for transwell images and 500 μm for wound healing images. Data are presented as mean ± s.d. Two-group comparisons were performed using Student’s *t* test, and multiple groups were analyzed by one-way ANOVA. ns, not significant, * *p* < 0.05, ** *p* < 0.01, *** *p* < 0.001, **** *p* < 0.0001; Figure S4: ITGA11-regulated FAK promotes malignant progression in HGC-27 cells. (A–C) Gene (A) and protein (B,C) expression of FAK(PTK2)/p-FAK following ITGA11 overexpression in HGC-27 cells (n = 6 for panel (A), and n = 3 for panels (B,C)). (D–F) Gene (D) and protein (E,F) expression of FAK(PTK2)/p-FAK following ITGA11 knockdown in HGC-27 cells (n = 4 for panel (D), and n = 3 for panels (E,F)). (G–I) Gene (G) and protein (H,I) expression of FAK(PTK2) with FAK overexpression (oeFAK) in HGC-27 cells (n = 6 for panel (G), and n = 3 for panels (H,I)). (J–L) Gene (J) and protein (K,L) expression of FAK(PTK2) with FAK knockdown (shFAK-1/2) in HGC-27 cells (n = 4 for panel (J), and n = 3 for panels (K,L)). (M,N) Representative images of wound healing, transwell and colony formation assays with FAK overexpression (M) and knockdown (N) in HGC-27 cells. (O,P) CCK-8 assay with FAK overexpression (O) and knockdown (P) in HGC-27 cells (n = 3). (Q,R) Quantification of functional assays with FAK overexpression (Q) and knockdown (R) in HGC-27 cells (n = 3). Scale bars: 100 μm for transwell images and 500 μm for wound healing images. Data are presented as mean ± s.d. Two-group comparisons were performed using Student’s *t* test, and multiple groups were analyzed by one-way ANOVA. ns, not significant, * *p* < 0.05, ** *p* < 0.01, *** *p* < 0.001; Figure S5: ITGA11 activates the FAK/PI3K/AKT/mTOR axis in HGC-27 cells. (A) Representative immunoblot images of FAK/PI3K/AKT/mTOR axis following ITGA11 overexpression in HGC-27 cells. (B) Quantification of FAK/PI3K/AKT/mTOR axis following ITGA11 overexpression in HGC-27 cells. (C) Representative immunoblot images of FAK/PI3K/AKT/mTOR axis following ITGA11 knockdown in HGC-27 cells. (D) Quantification of FAK/PI3K/AKT/mTOR axis following ITGA11 knockdown in HGC-27 cells. (E) Representative immunoblot images of ITGA11 and FAK/PI3K/AKT/mTOR axis following ITGA11 overexpression and FAK knockdown in HGC-27 cells. (F) Quantification of ITGA11 and FAK/PI3K/AKT/mTOR axis following ITGA11 overexpression and FAK knockdown in HGC-27 cells. (G) Representative images of wound healing, transwell and colony formation assays following ITGA11 overexpression and FAK knockdown in HGC-27 cells. (H) CCK-8 assay following ITGA11 overexpression and FAK knockdown in HGC-27 cells. (I) Quantification of functional assays following ITGA11 overexpression and FAK knockdown in HGC-27 cells. Scale bars: 100 μm for transwell images and 500 μm for wound healing images. Data are presented as mean ± s.d. (n = 3 per group). Two-group comparisons were performed using Student’s *t* test, and multiple groups were analyzed by one-way ANOVA. ns, not significant, * *p* < 0.05, ** *p* < 0.01, *** *p* < 0.001, **** *p* < 0.0001; Figure S6: Naringenin suppresses ITGA11-associated FAK/PI3K/AKT/mTOR signaling and malignant phenotypes in HGC-27 cells. (A) Representative immunoblot images of ITGA11 and FAK/PI3K/AKT/mTOR axis following ITGA11 overexpression and Naringenin treatment (20, 40, 80, and 160 µM for 24 h) in HGC-27 cells. (B) Quantification of gene (n = 4) and protein (n = 3) expression of ITGA11 and FAK/PI3K/AKT/mTOR axis following ITGA11 overexpression and Naringenin treatment (20, 40, 80, and 160 µM for 24 h) in HGC-27 cells. (C) Representative images of wound healing, transwell and colony formation assays following ITGA11 overexpression and Naringenin treatment (40 µM; 24 h) in HGC-27 cells. (D) Quantification of functional assays following ITGA11 overexpression and Naringenin treatment (40 µM; 24 h) in HGC-27 cells (n = 3). (E,F) Gene (E) and protein (F) expression of ITGA11 following ITGA11 knockdown and Naringenin treatment (40 μM; 24 h) in HGC-27 cells (n = 4 for panel (E), and n = 3 for panel (F)). (G) Representative images of wound healing and transwell assays following ITGA11 knockdown and Naringenin treatment (40 µM; 24 h) in HGC-27 cells. (H) Quantification of functional assays following ITGA11 knockdown and Naringenin treatment (40 µM; 24 h) in HGC-27 cells (n = 3). Scale bars: 100 μm for transwell images and 500 μm for wound healing images. Data are presented as mean ± s.d. Multiple groups were analyzed by one-way ANOVA. ns, not significant, * *p* < 0.05, ** *p* < 0.01, *** *p* < 0.001; Table S1: Baseline characteristics of the patient cohort (N = 60); Table S2: Main reagents in study; Table S3: Main instruments in study; Table S4: Primer, shRNA, and siRNA sequences used in the study; Table S5: Effect sizes for all *p*-values; Table S6: Univariate and multivariate Cox regression analyses in the TCGA-STAD cohort (N = 370); Table S7: Univariate and multivariate Cox regression analyses in our institutional cohort (N = 60); Table S8: Top fifteen candidate compounds with the lowest predicted binding energy for ITGA11; Table S9: (A) Body weight (g) of mice in Group oe-ITGA11 and oeITGA11 + Naringenin; (B) Body weight (g) of mice in Group shNC and shITGA11-2; (C) Tumor volume (mm^3^) of mice in Group oe-ITGA11 and oeITGA11 + Naringenin; (D) Tumor volume (mm^3^) of mice in Group shNC and shITGA11-2.

## Abbreviations

The following abbreviations are used in this manuscript:

GC	Gastric cancer
ECM	Extracellular matrix
ITGA11	Integrin alpha 11
TME	Tumor microenvironment
TCGA	The Cancer Genome Atlas
STAD	Stomach Adenocarcinoma cohort from TCGA
DEGs	Differentially expressed genes
PPI	Protein–protein interaction
GO	Gene Ontology
KEGG	Kyoto Encyclopedia of Genes and Genomes
GSEA	Gene Set Enrichment Analysis
DARTS	Drug Affinity Responsive Target Stability
CETSA	Cellular thermal shift assay
