# Supplementary material for: Naringenin, a Food-Derived Flavanone, Suppresses ITGA11-Associated Gastric Cancer Progression via the FAK/PI3K/AKT/mTOR Axis
Source: Cancers (Basel). 2026 May 24;18(11):1712. doi: 10.3390/cancers18111712 (PMC13255981; doi:10.3390/cancers18111712)
Supplement: Supplementary file 1 [file cancers-18-01712-s001.zip › File S1. Original Images for Blots.pdf]

Supplementary raw data of WB

(1) Whole uncropped membrane images (Supplementary data added during rivision):
Figure 5A, 5C, 5E, 6A, 6F, 6H, 7B, 7D, 7I, S5A, S5C, S5E, S6A, S6F

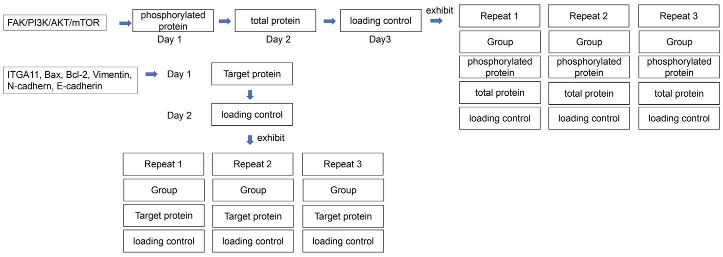

(2) Uncropped membrane images (not whole membrane, supplementary data at the initial submission):
Figure 3E, 3J, 3M, 3P, 4H, 4K, 4N, 4Q, 8J, 8M, S3B, S3F, Figure S4H, K

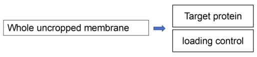

(3) Uncropped membrane images using parallel gels (not whole membrane, supplementary data at the initial submission):
Figure 4B, 4E, S4B, S4E

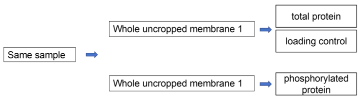

Figure 3E

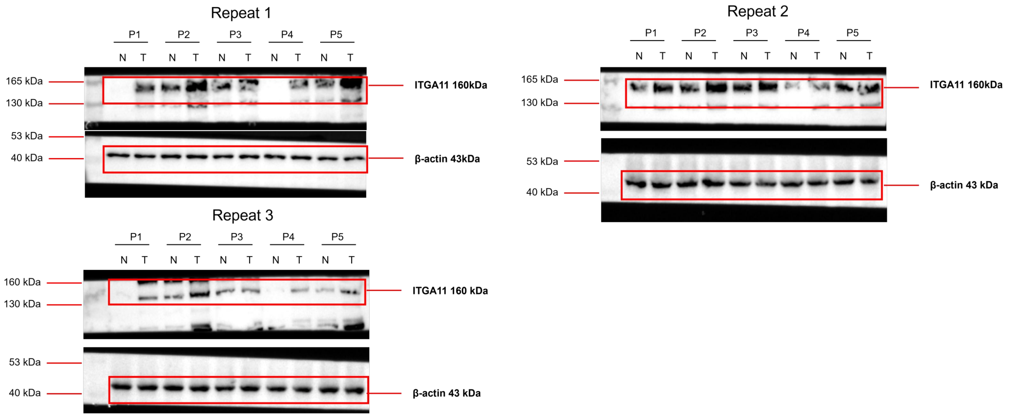

Figure 3J

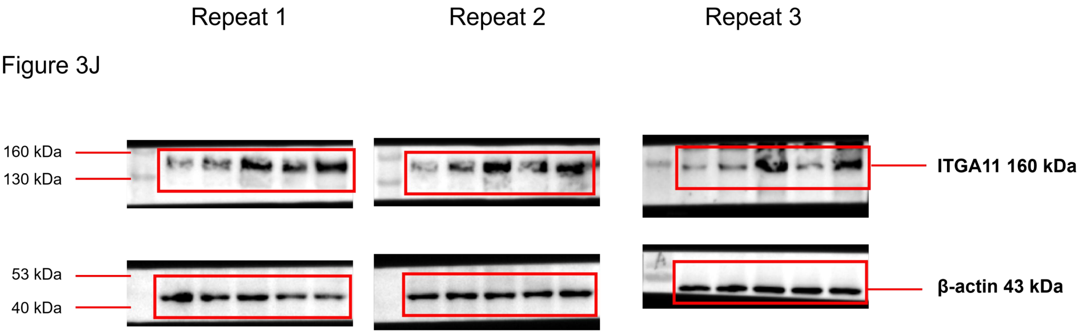

Figure 3M

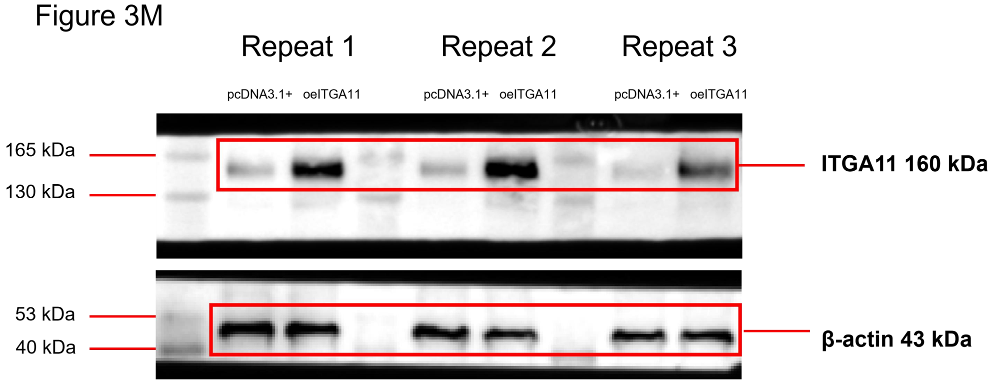

Figure 3P

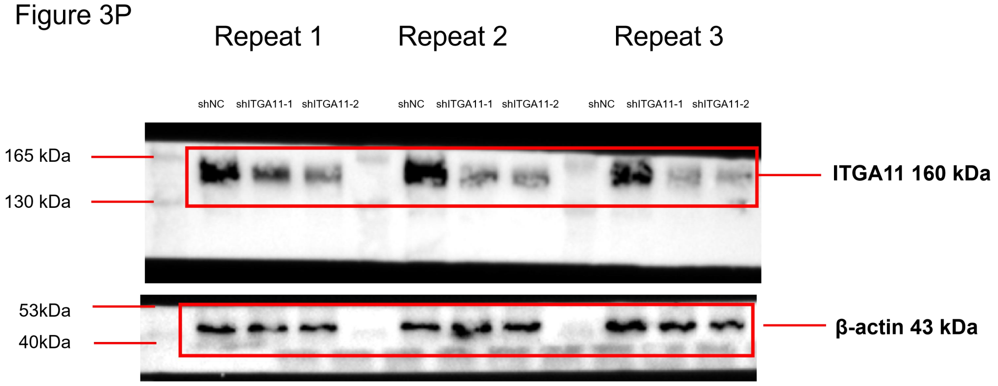

Figure S3B

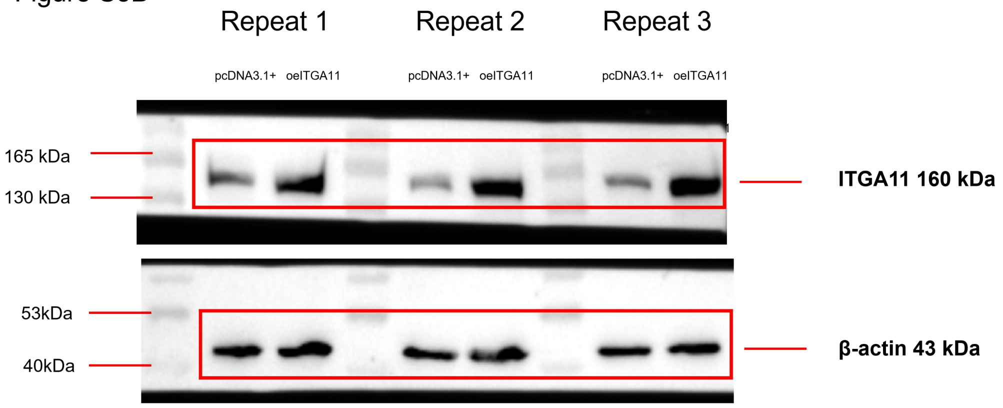

Figure S3F

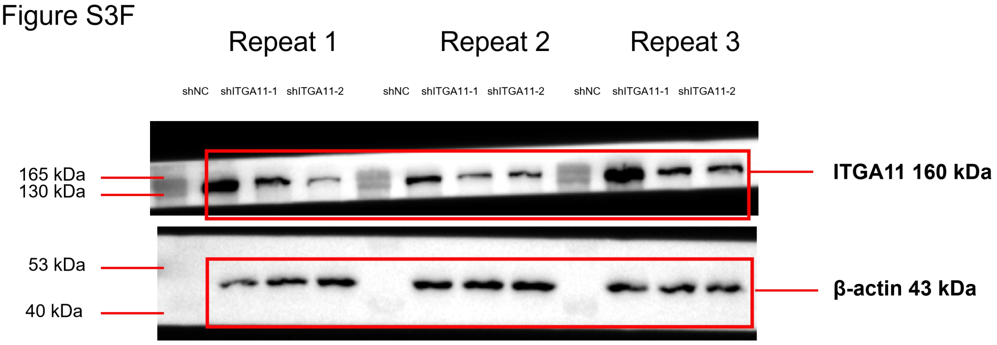

Figure 4B

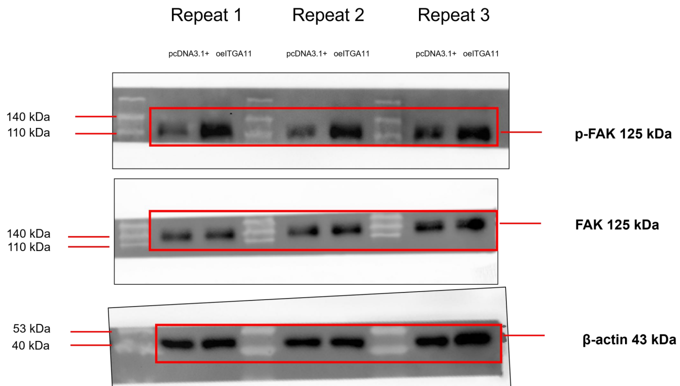

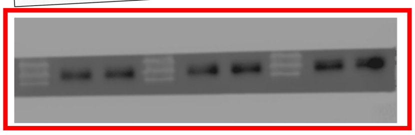

Note: The Bio-Rad membrane imaging system produced images with a white background and relatively dark bands. Therefore, we enhanced the contrast of the strip image only to clearly show that the bands had not been cropped. No contrast adjustment was performed on the original Western blot (WB) image used as raw data.

Figure 4E

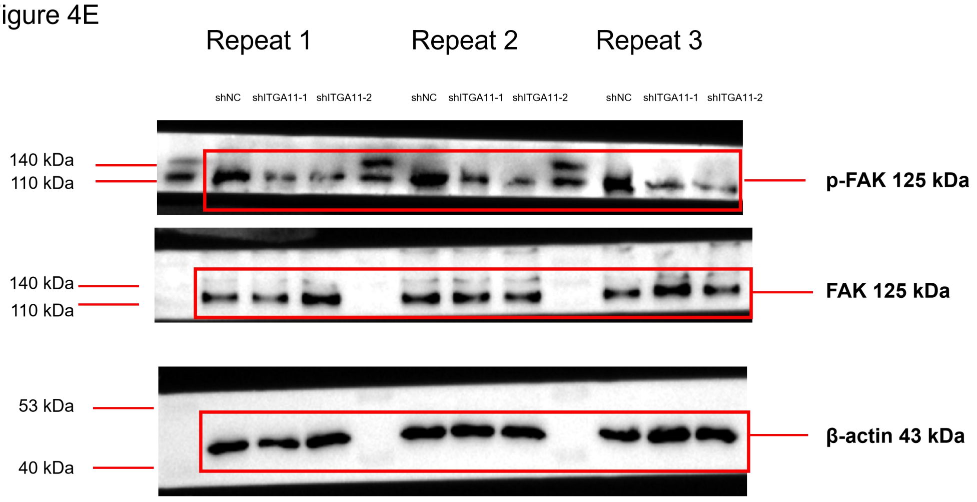

Figure 4H

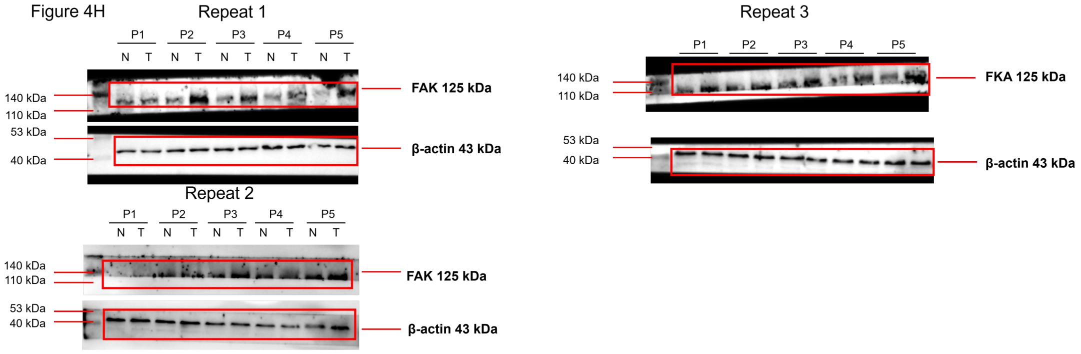

Figure 4K

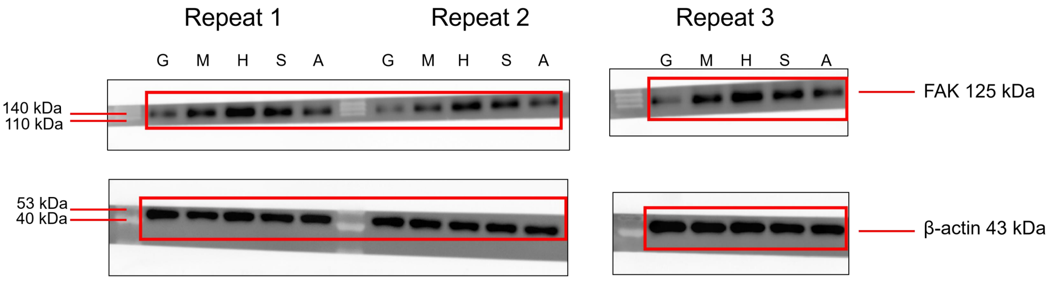

Figure 4N

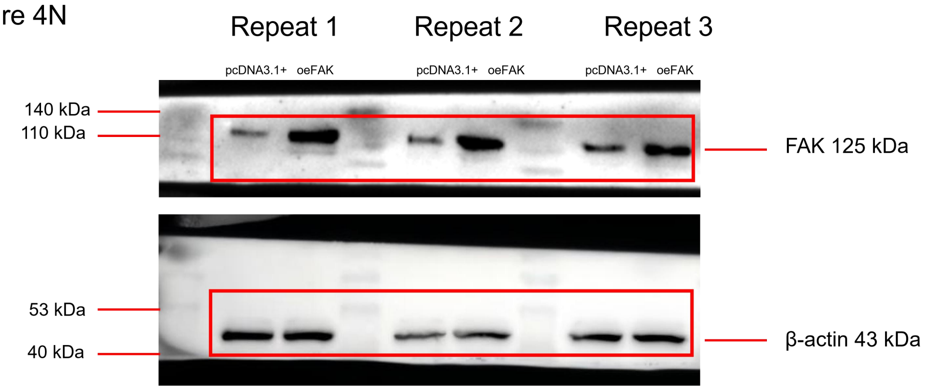

Figure 4Q

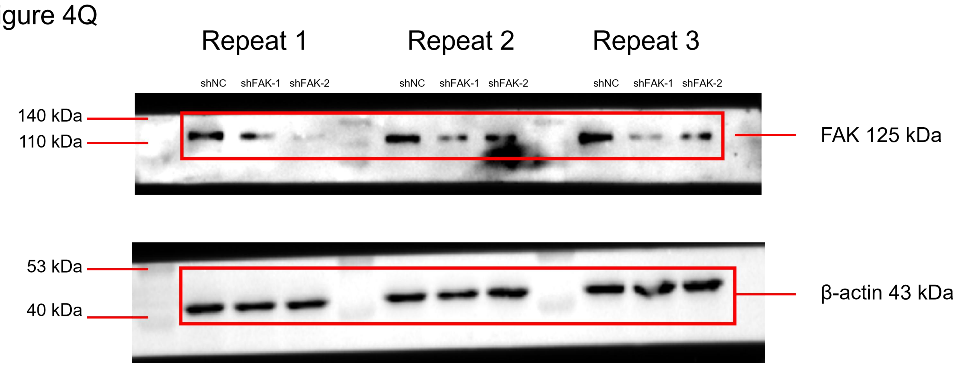

Figure S4B

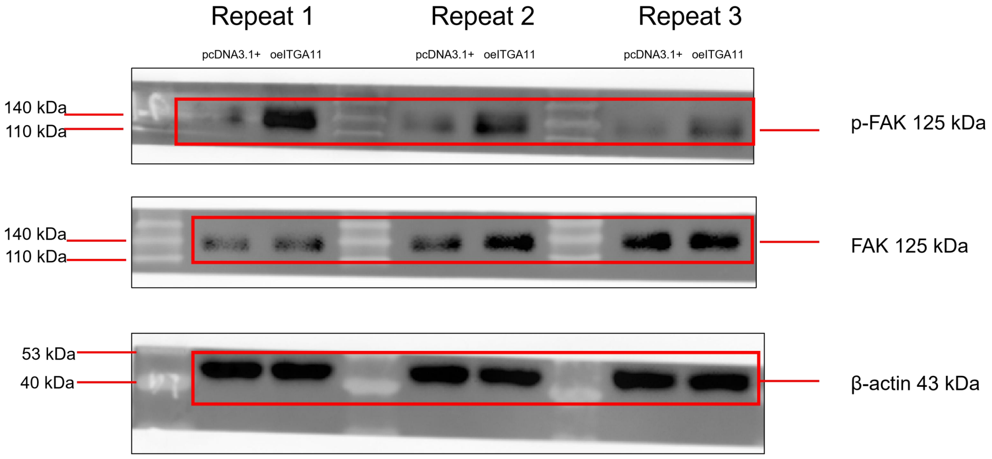

Figure S4E

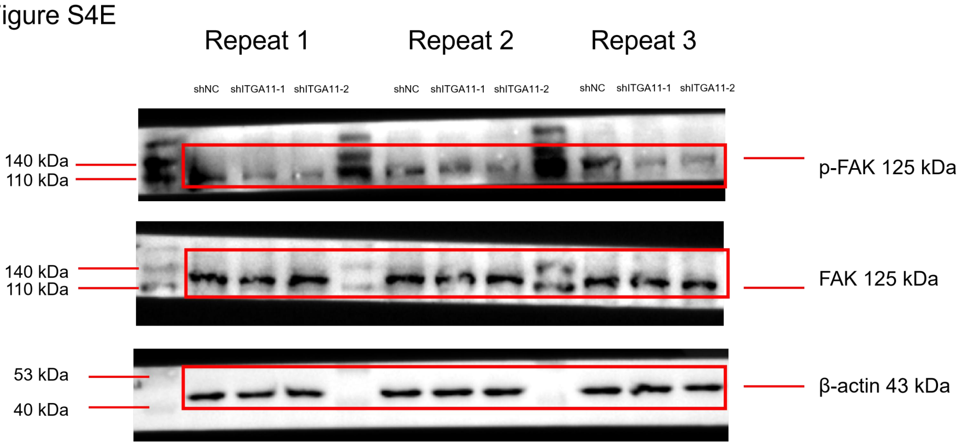

Figure S4H

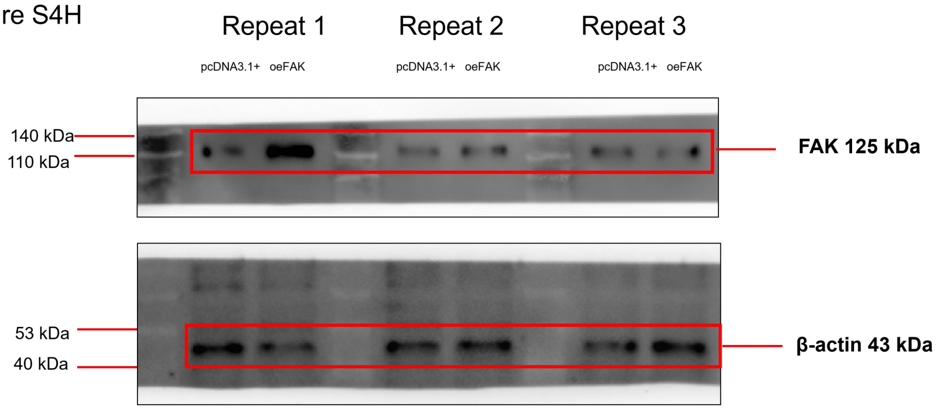

Figure S4K

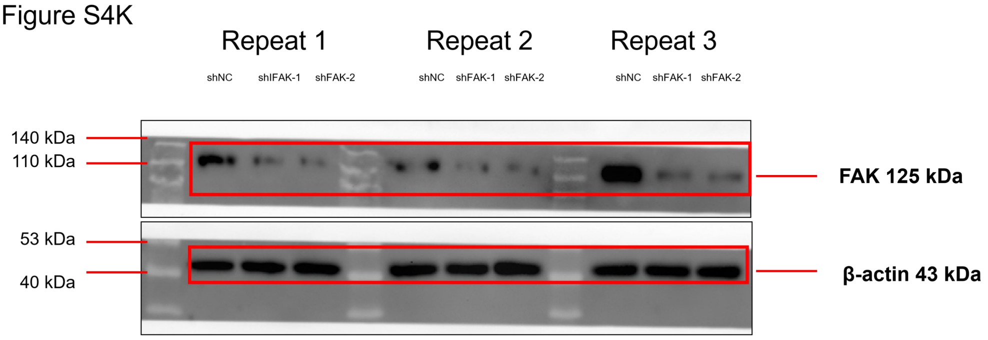

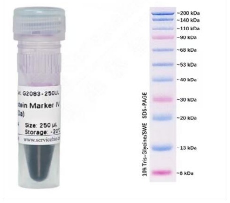

Maker for ITGA11, FAK, PI3K, AKT, BAX, Bcl-2, E-cadherin, N-cadherin, Vimentin, and β-actin

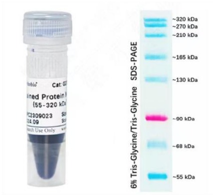

maker for mTOR

Figure 5A

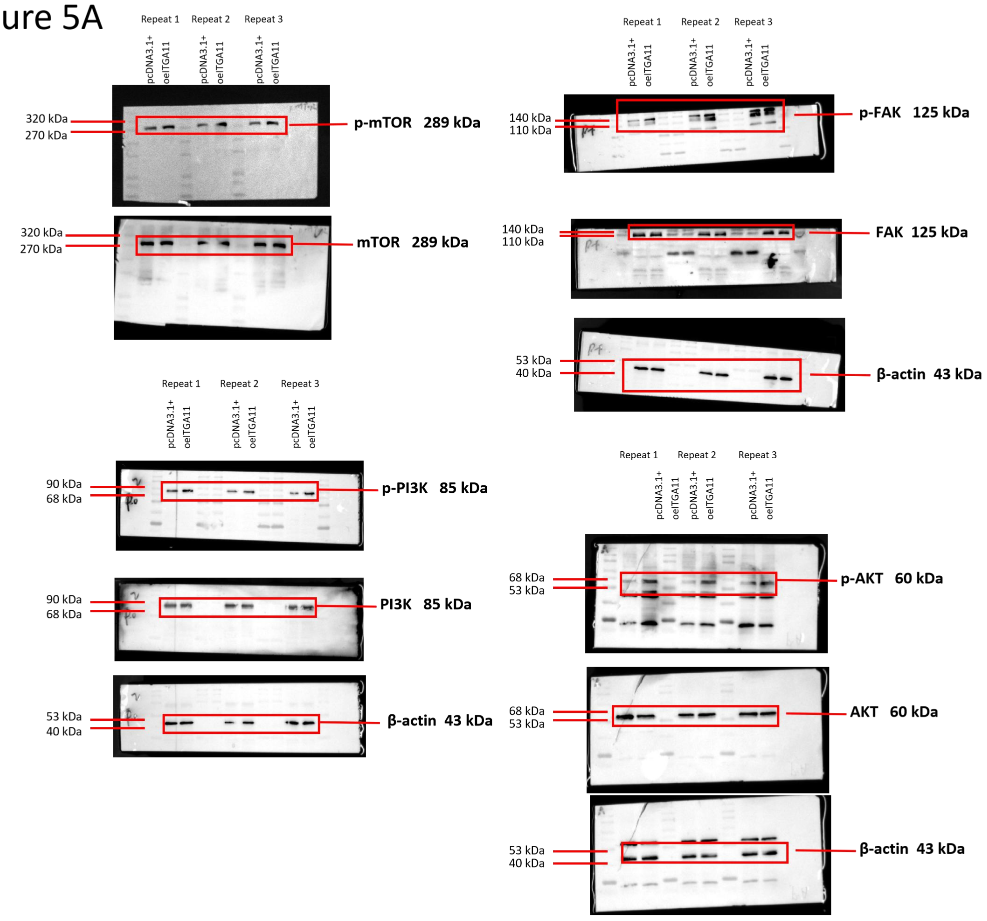

Figure 5C

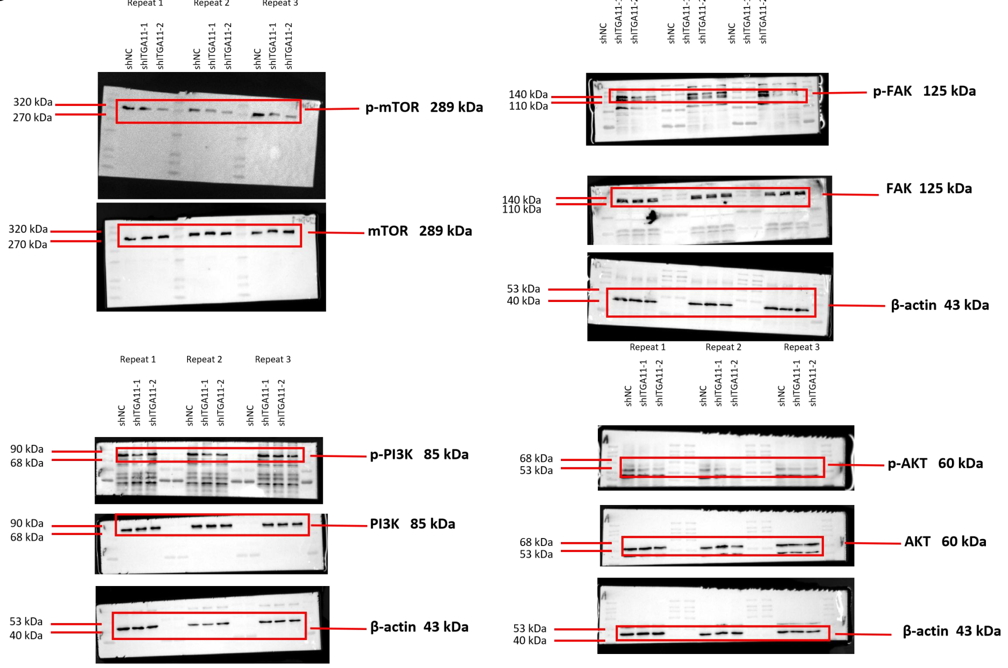

Figure 5E

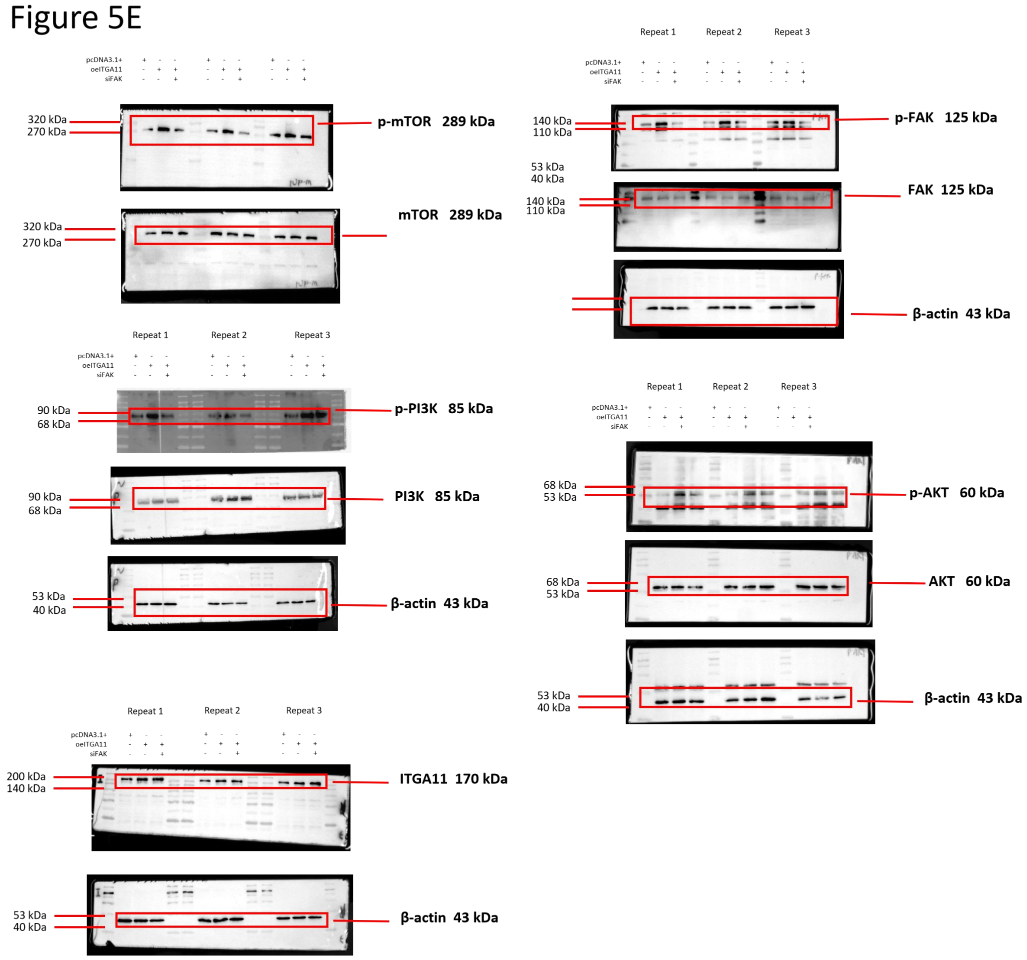

Figure 6A

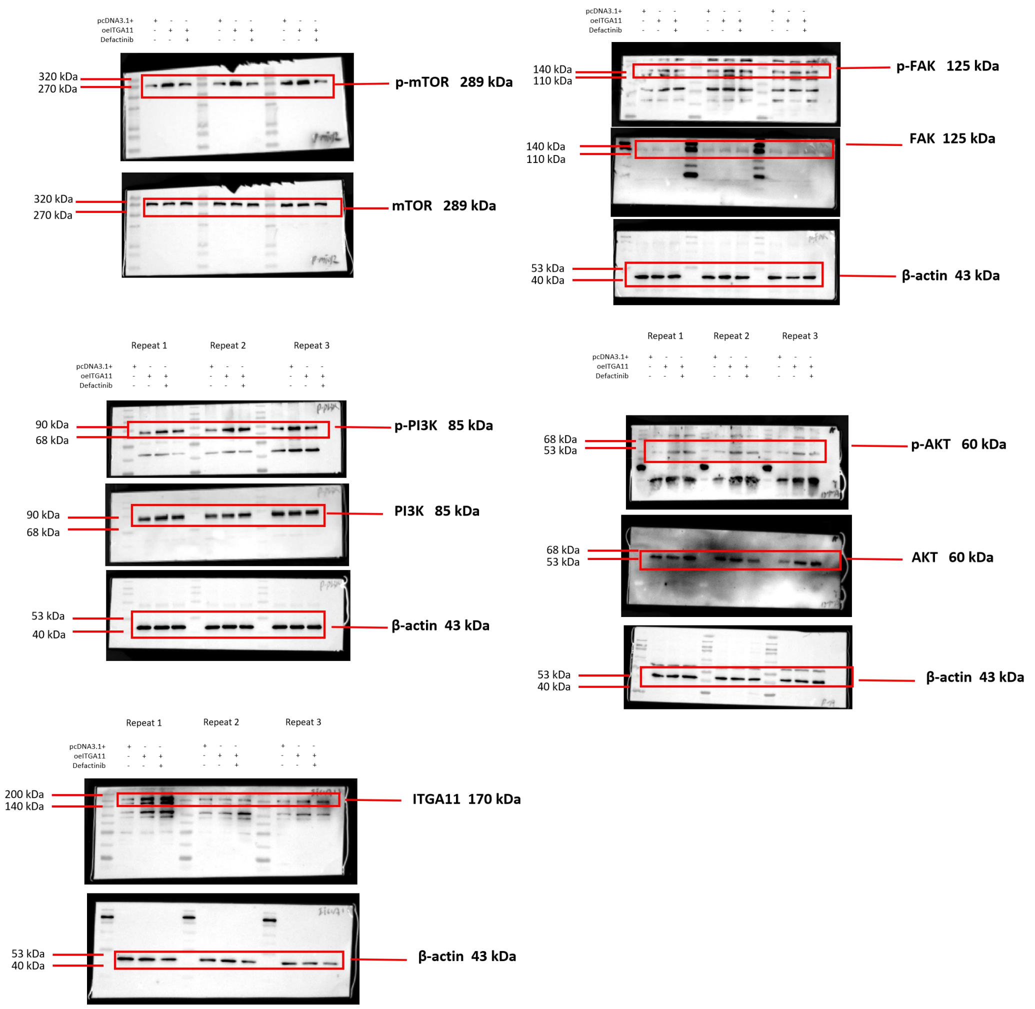

Figure 6F

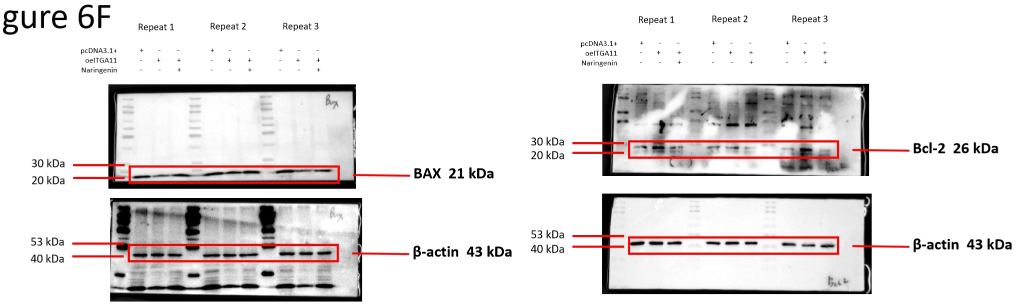

Figure 6H

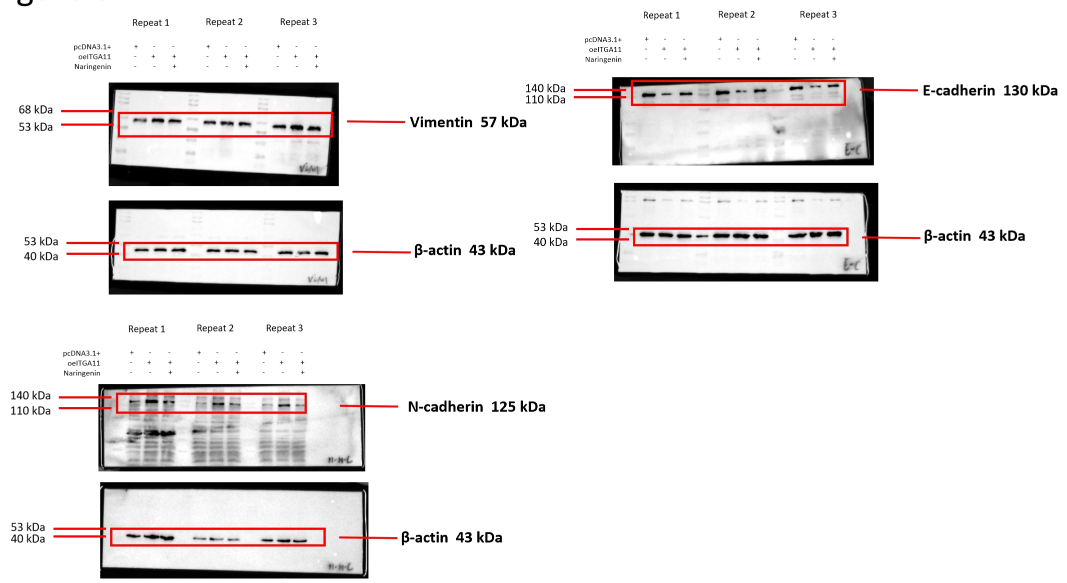

Figure 7 B

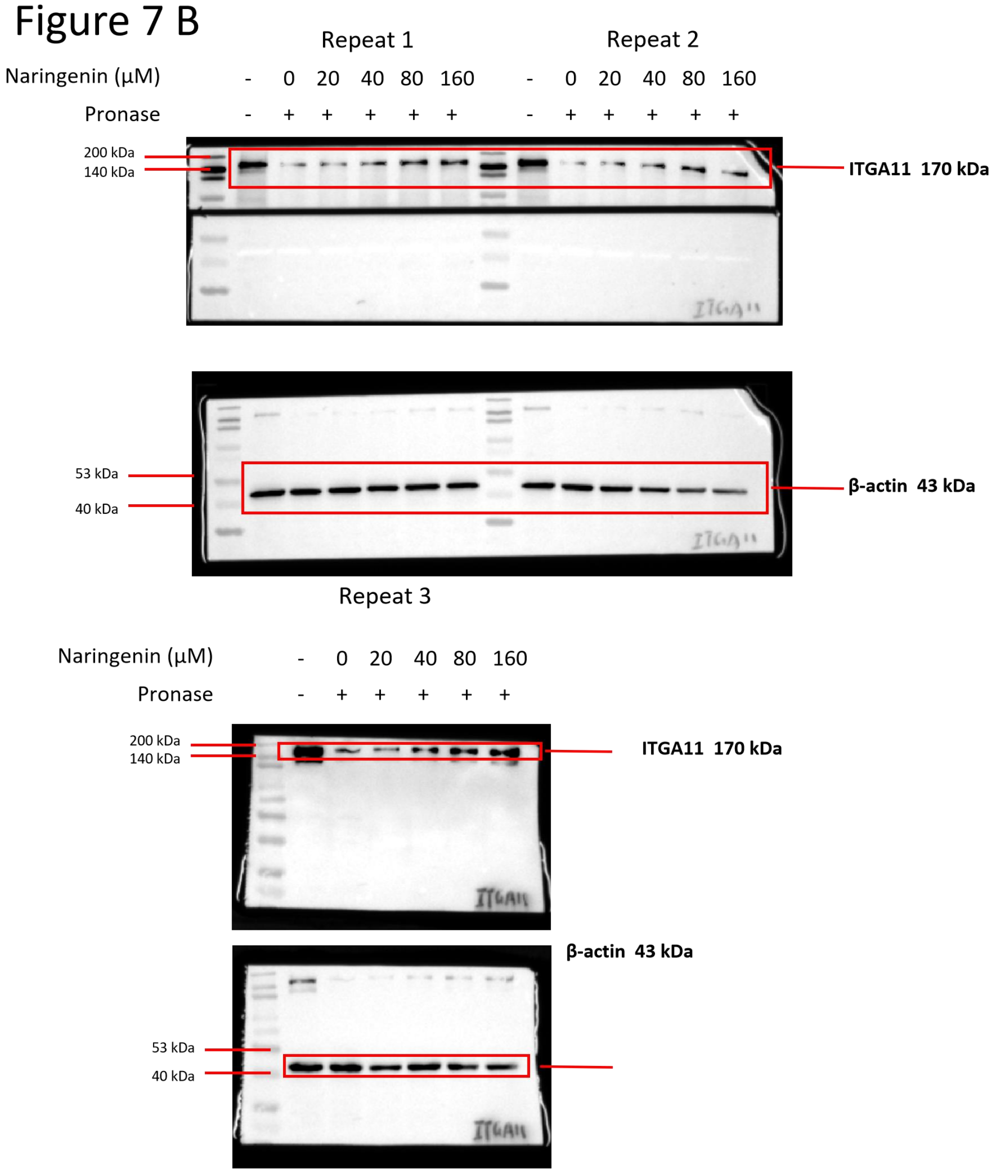

Figure 7 D

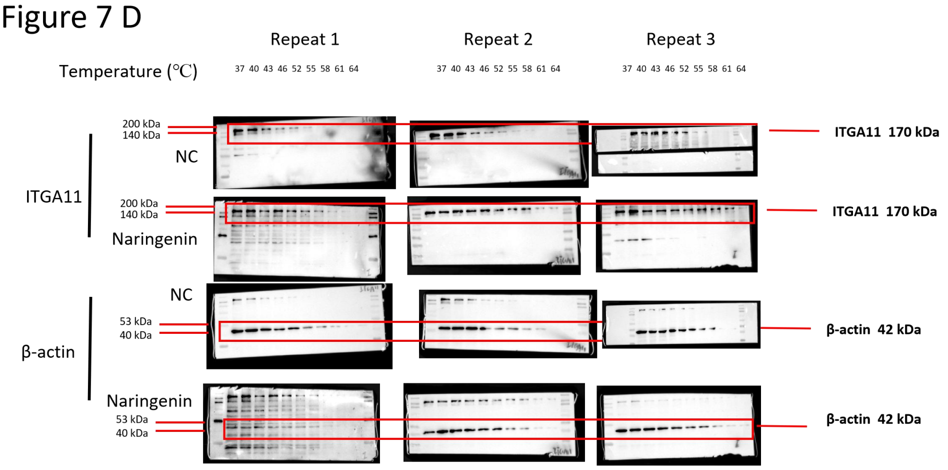

Repeat 1
Repeat 2
Repeat 3
Temperature (℃)
37 40 43 46 52 55 58 61 64
37 40 43 46 52 55 58 61 64
37 40 43 46 52 55 58 61 64
ITGA11
200 kDa
140 kDa
NC
ITGA11 170 kDa
200 kDa
140 kDa
Naringenin
ITGA11 170 kDa
β-actin
NC
53 kDa
40 kDa
β-actin 42 kDa
Naringenin
53 kDa
40 kDa
β-actin 42 kDa

Figure 7I

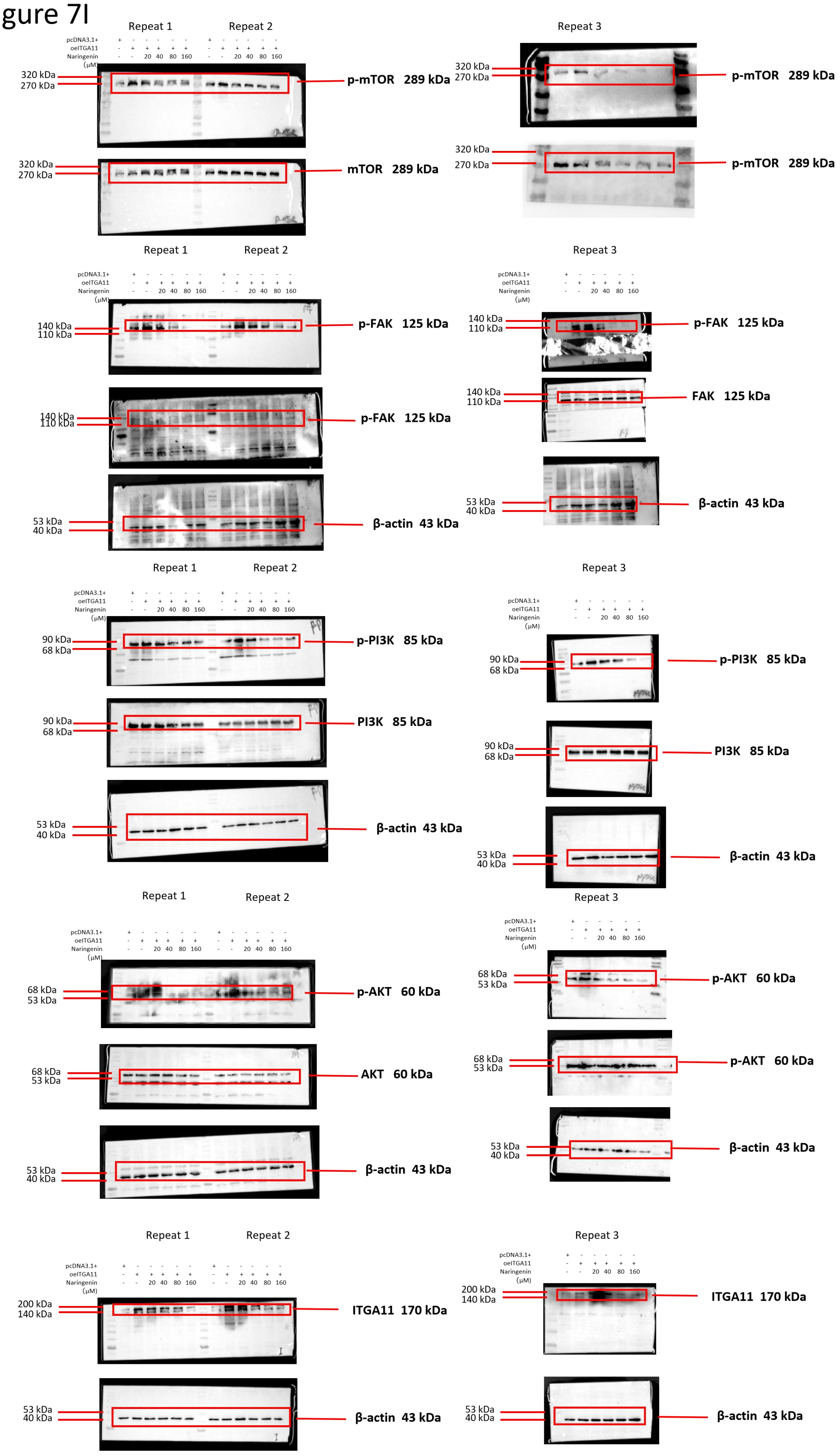

Repeat 1
Repeat 2
Repeat 3
pcDNA3.1+
oeITGA11
Naringenin (μM)
20 40 80 160
320 kDa
270 kDa
p-mTOR 289 kDa
mTOR 289 kDa
140 kDa
110 kDa
p-FAK 125 kDa
FAK 125 kDa
53 kDa
40 kDa
β-actin 43 kDa
90 kDa
68 kDa
p-PI3K 85 kDa
PI3K 85 kDa
68 kDa
53 kDa
p-AKT 60 kDa
AKT 60 kDa
200 kDa
140 kDa
ITGA11 170 kDa

Figure S5A

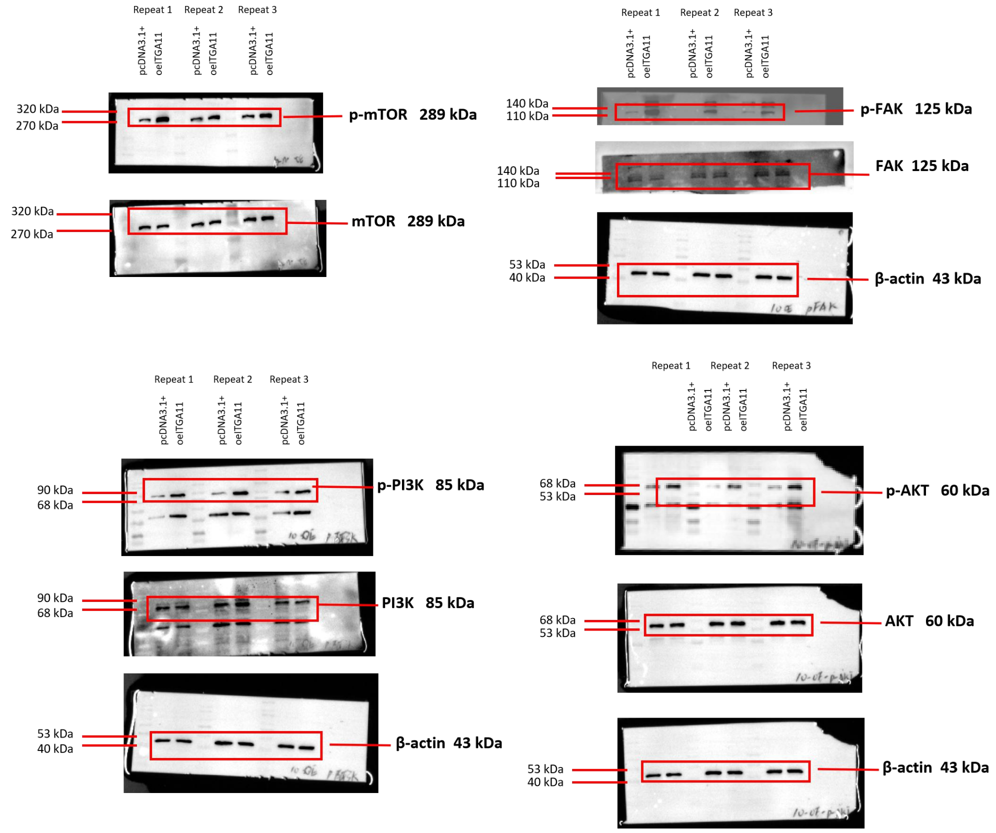

Figure S5C

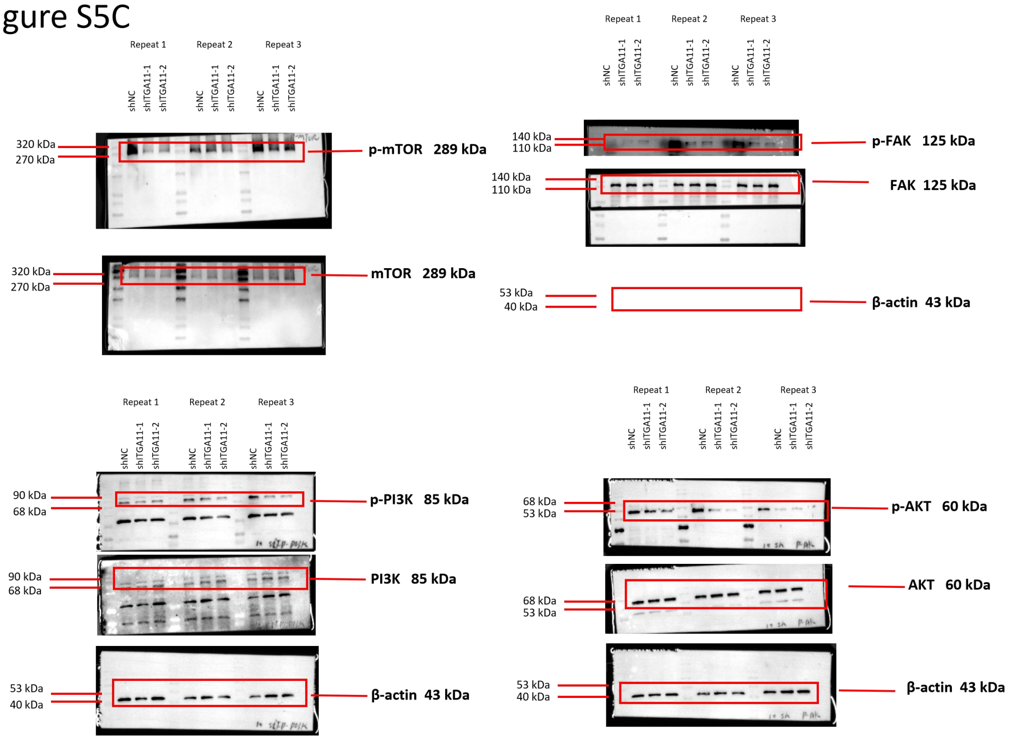

Figure S5E

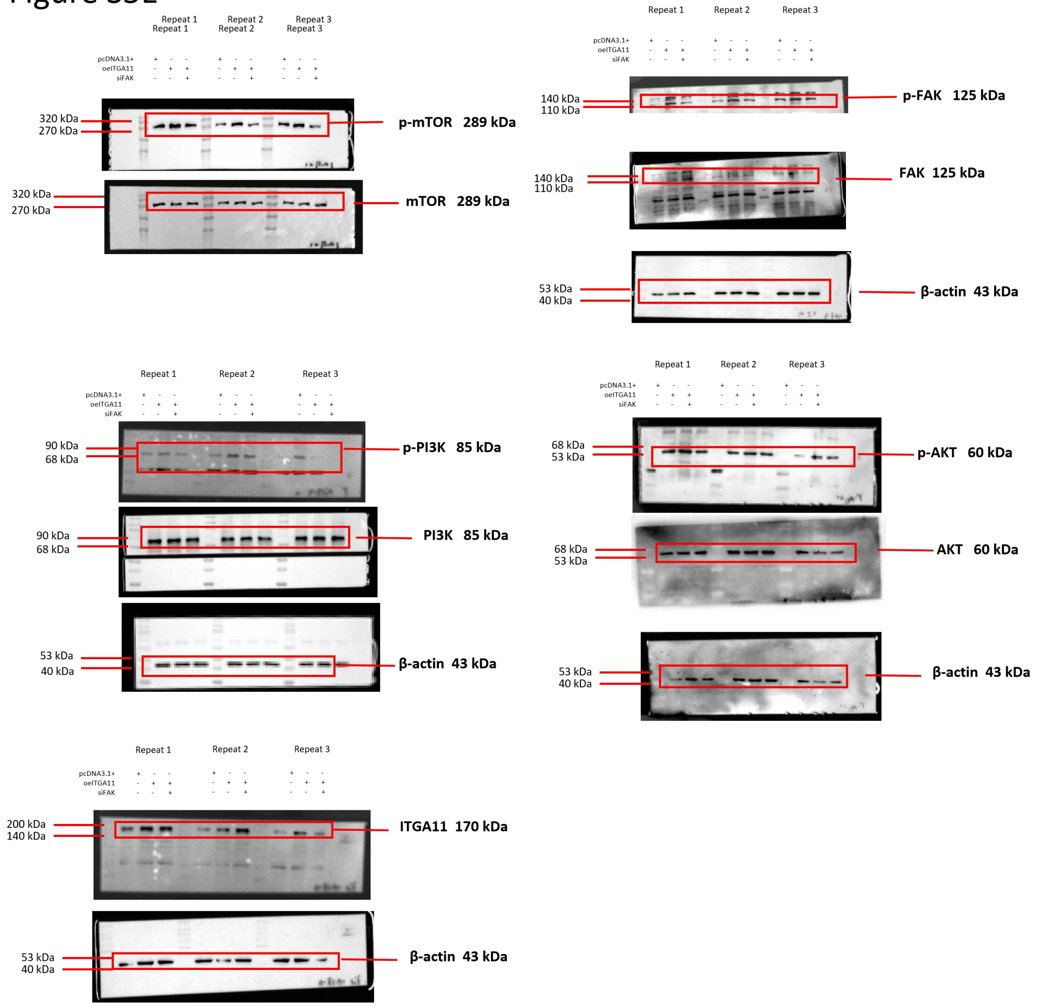

Repeat 1
Repeat 2
Repeat 3
pcDNA3.1+
oeITGA11
siFAK
320 kDa
270 kDa
p-mTOR 289 kDa
mTOR 289 kDa
140 kDa
110 kDa
p-FAK 125 kDa
FAK 125 kDa
53 kDa
40 kDa
β-actin 43 kDa
90 kDa
68 kDa
p-PI3K 85 kDa
PI3K 85 kDa
p-AKT 60 kDa
AKT 60 kDa
200 kDa
140 kDa
ITGA11 170 kDa

Figure S6A

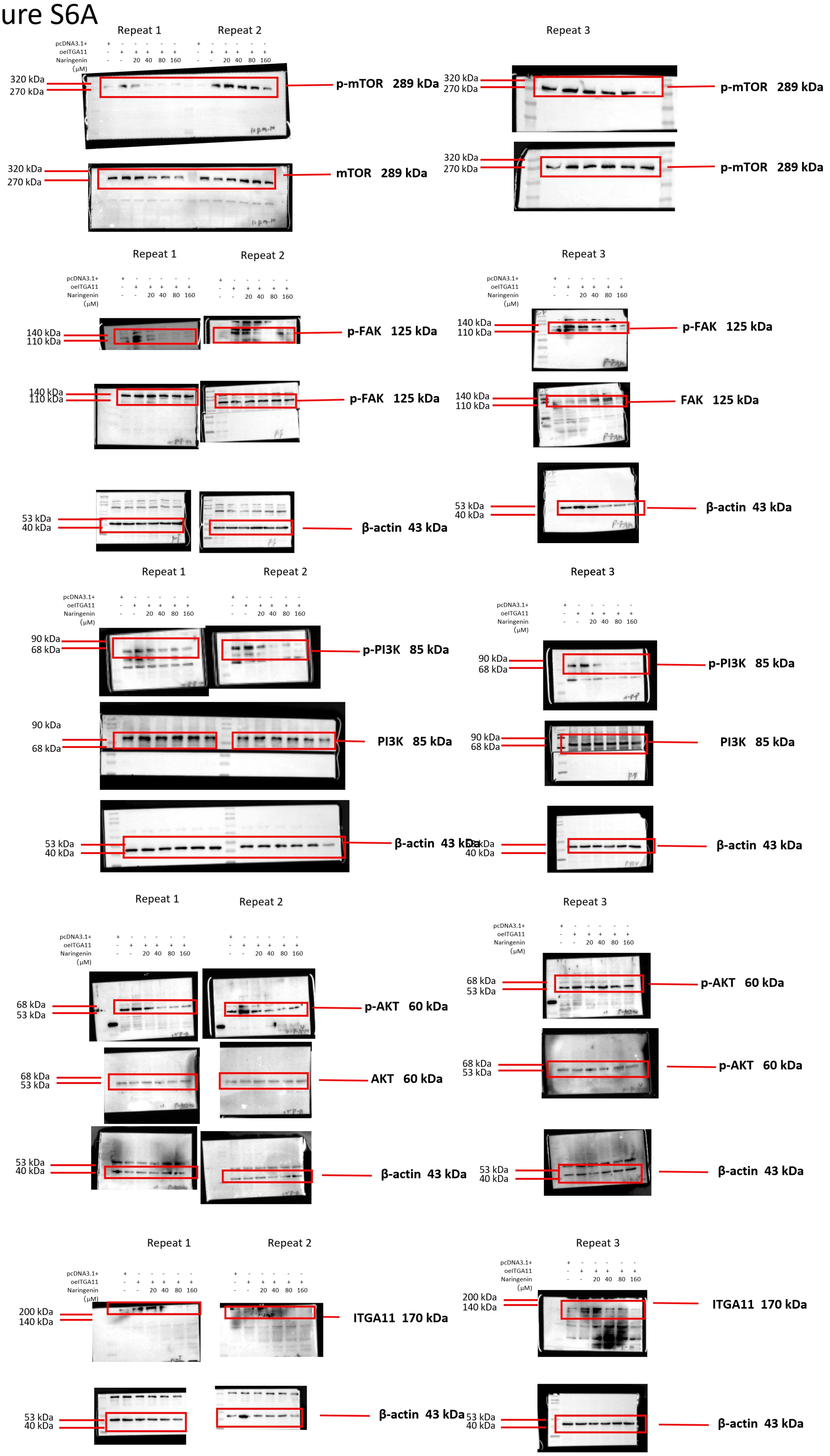
Repeat 1
Repeat 2
Repeat 3
pcDNA3.1+
oeITGA11
Naringenin (μM)
20 40 80 160
320 kDa
270 kDa
p-mTOR 289 kDa
mTOR 289 kDa
140 kDa
110 kDa
p-FAK 125 kDa
FAK 125 kDa
53 kDa
40 kDa
β-actin 43 kDa
90 kDa
68 kDa
p-PI3K 85 kDa
PI3K 85 kDa
68 kDa
53 kDa
p-AKT 60 kDa
AKT 60 kDa
200 kDa
140 kDa
ITGA11 170 kDa

Figure S6F

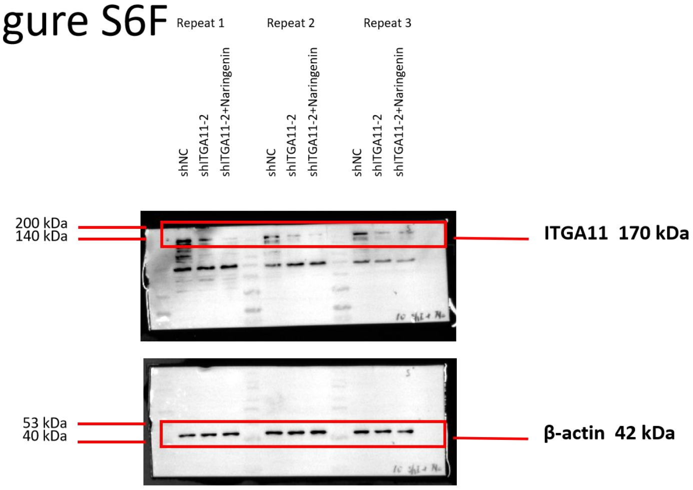
Repeat 1
Repeat 2
Repeat 3
shNC
shITGA11-2
shITGA11-2+Naringenin
shNC
shITGA11-2
shITGA11-2+Naringenin
shNC
shITGA11-2
shITGA11-2+Naringenin
200 kDa
140 kDa
ITGA11 170 kDa
53 kDa
40 kDa
β-actin 42 kDa
