# Supplementary material for: Naringenin, a Food-Derived Flavanone, Suppresses ITGA11-Associated Gastric Cancer Progression via the FAK/PI3K/AKT/mTOR Axis
Source: Cancers (Basel). 2026 May 24;18(11):1712. doi: 10.3390/cancers18111712 (PMC13255981; doi:10.3390/cancers18111712)
Supplement: Supplementary file 1 [file cancers-18-01712-s001.zip › Supplementary Figures S1-S6.pdf]

Supplementary Figures for

Naringenin, a Food-Derived Flavanone, Suppresses ITGA11-Associated Gastric Cancer Progression via the FAK/PI3K/AKT/mTOR Axis

Qiang Li, Guiyang Ye, Fangfang Chen, Qiushuang Wang, Junfeng Yan, Yi Wang * and Qiang Tong *

* Correspondence: w_yi2022@163.com (Y.W.); qiangtong@whu.edu.cn (Q.T.)

The file includes:

Figure S1; Figure S2; Figure S3; Figure S4; Figure S5; Figure S6

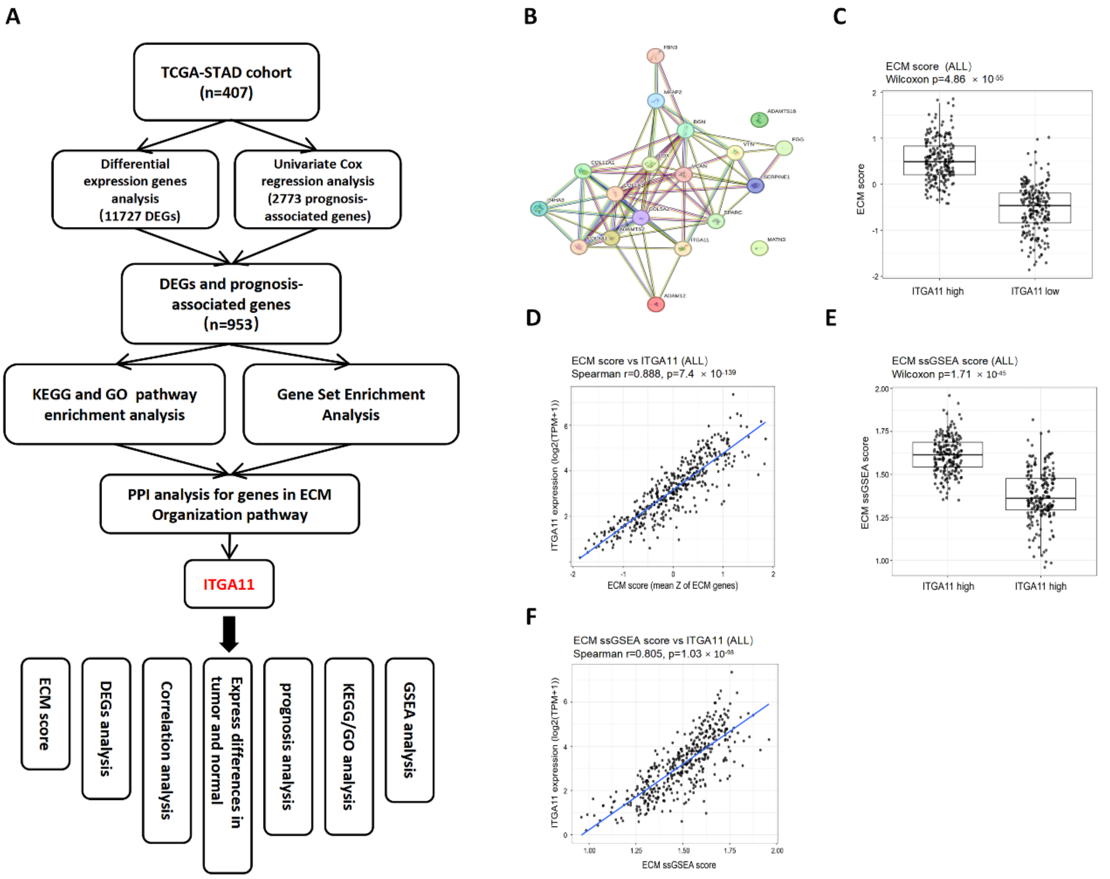

Figure S1. Schematic workflow of the bioinformatic analysis and complementary ITGA11-related bioinformatic analyses. (A) Schematic workflow of the bioinformatic analysis. (B) PPI network of key genes in the ECM organization pathway. (C) ECM score in ITGA11-high versus ITGA11-low (All cohort). (D) Correlation between ITGA11 expression and ECM score (All cohort). (E) ECM ssGSEA score in ITGA11-high versus ITGA11-low (All cohort). (F) Correlation between ITGA11 expression and ECM ssGSEA score (All cohort). Data are presented as mean ± s.d. A total of 32 normal samples and 375 tumor samples in the TCGA-STAD cohort were analyzed.

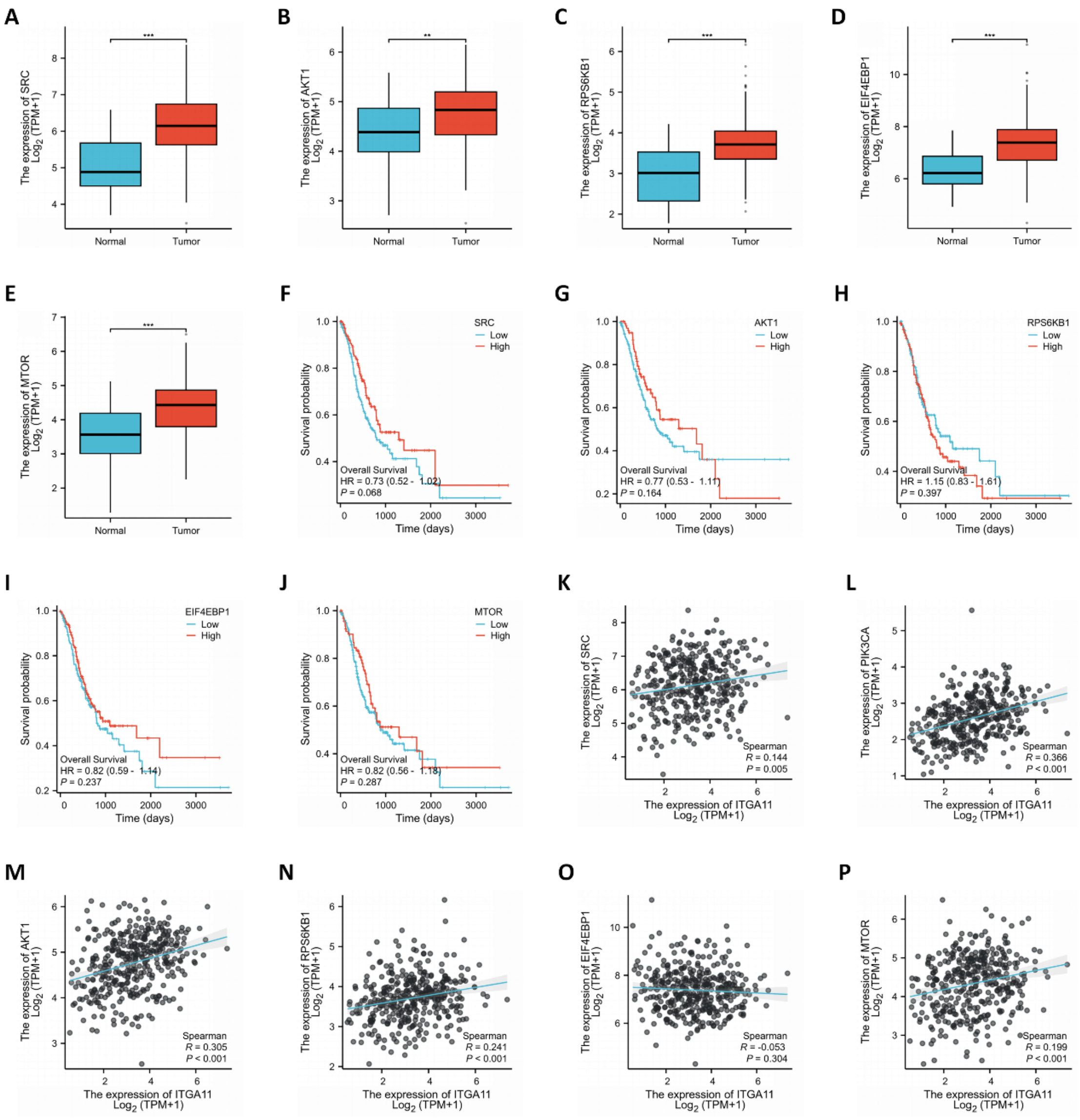

Figure S2. Bioinformatic analysis of FAK/PI3K/AKT/mTOR axis. (A–E) Gene expression of SRC (A), AKT1 (B), RPS6KB1 (C), EIF4EBP1 (D), MTOR (E) in tumor versus normal tissues. (F–J) Kaplan-Meier curves for OS stratified by gene expression of SRC (F), AKT1 (G), RPS6KB1 (H), EIF4EBP1 (I), MTOR (J). (K–P) Correlation analyses between ITGA11 and gene expression of SRC (K), PIK3CA (L), AKT1 (M), RPS6KB1 (N), EIF4EBP1 (O), MTOR (P). Data are presented as mean ± s.d. A total of 32 normal samples and 375 tumor samples in TCGA-STAD cohort were analyzed. ** $p < 0.01$, *** $p < 0.001$.

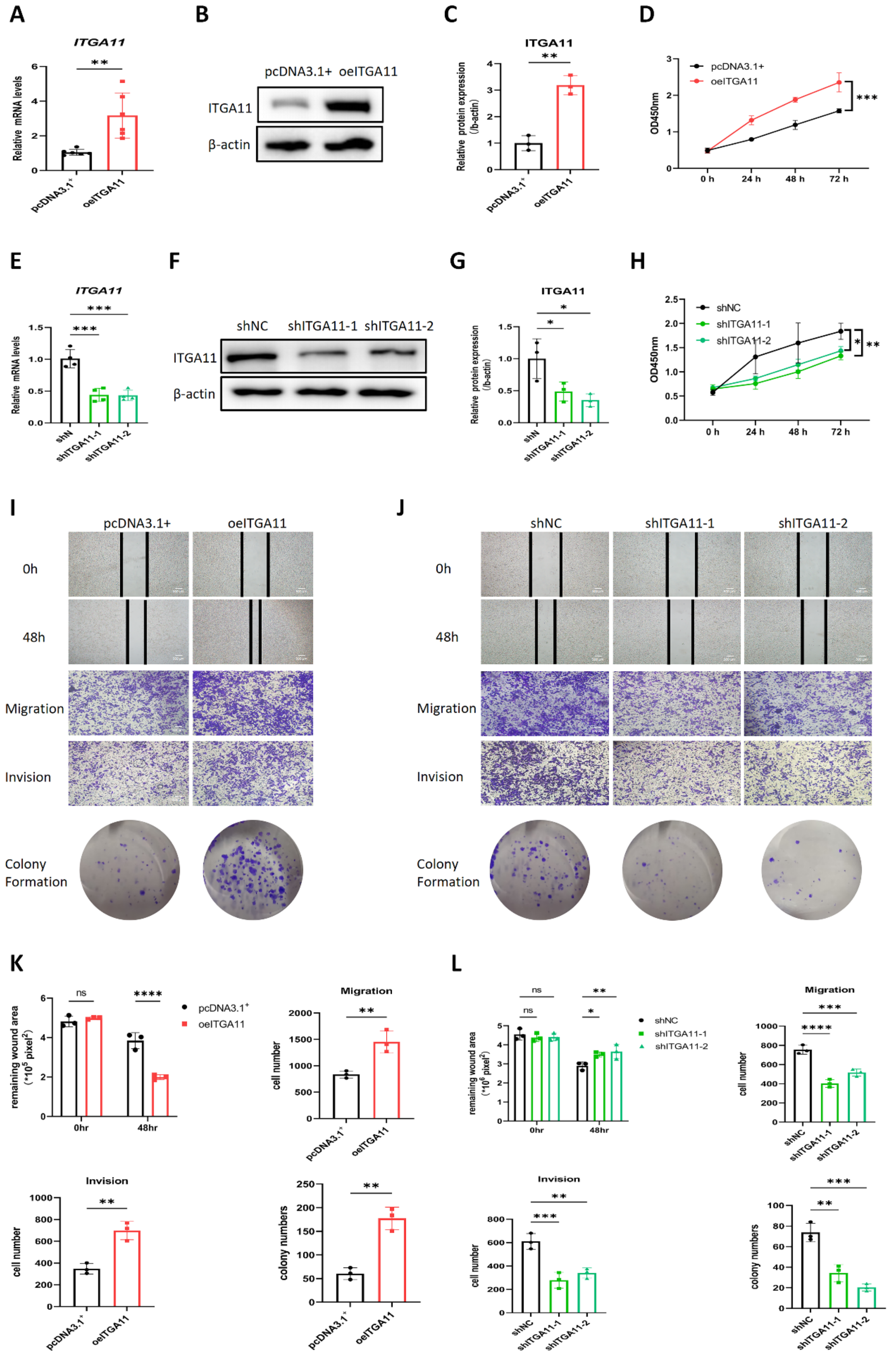

Figure S3. ITGA11 promotes proliferation, migration, invasion, and colony formation in HGC-27 cells. (A–C) Gene (A) and protein (B,C) expression of ITGA11 following ITGA11

overexpression (oeITGA11) in HGC-27 cells (n = 6 for panel (A), and n = 3 for panels (B,C)). (D) CCK-8 assay following ITGA11 overexpression in HGC-27 cells (n = 3). (E-G) Gene (E) and protein (F-G) expression of ITGA11 following ITGA11 knockdown (shITGA11-1/2) in HGC-27 cells (n = 4 for panel (E), and n = 3 for panels (F,G)). (H) CCK-8 assay following ITGA11 knockdown in HGC-27 cells (n = 3). (I,J) Representative images of wound healing, transwell and colony formation assays following ITGA11 overexpression (I) and knockdown (J) in HGC-27 cells. (K,L) Quantification of functional assays following ITGA11 overexpression (K) and knockdown (L) in HGC-27 cells (n = 3). Scale bars: 100 μm for transwell images and 500 μm for wound healing images. Data are presented as mean ± s.d. Two-group comparisons were performed using Student's *t* test, and multiple groups were analyzed by one-way ANOVA. ns, not significant, * $p < 0.05$, ** $p < 0.01$, *** $p < 0.001$, **** $p < 0.0001$.

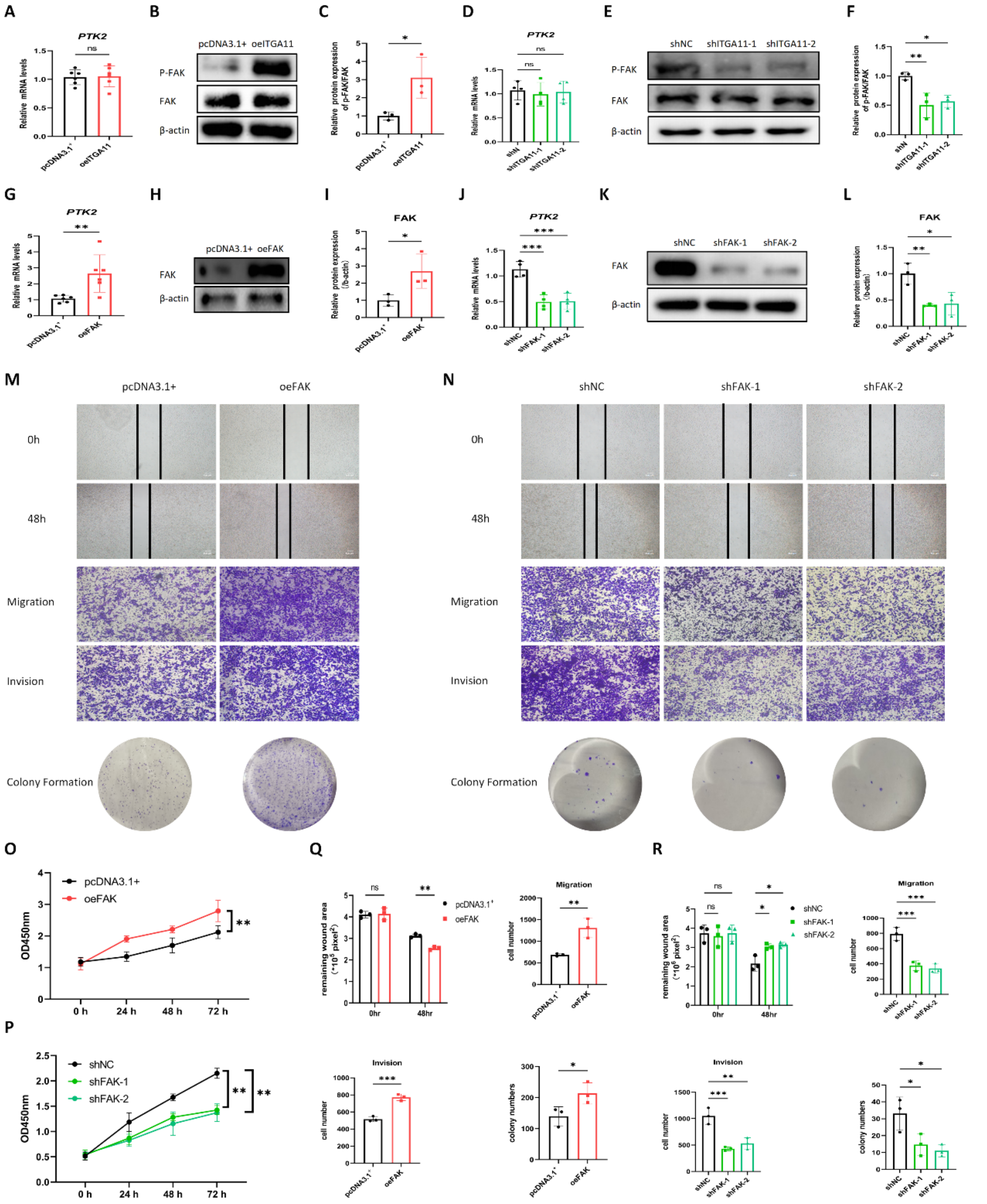

Figure S4. ITGA11-regulated FAK promotes malignant progression in HGC-27 cells. (A-C) Gene (A) and protein (B,C) expression of FAK(PTK2)/p-FAK following ITGA11 overexpression in HGC-27 cells (n = 6 for panel (A), and n = 3 for panels (B,C)). (D–F) Gene (D) and protein (E,F) expression of FAK(PTK2)/p-FAK following ITGA11 knockdown in HGC-27 cells (n = 4 for panel (D), and n = 3 for panels (E,F)). (G–I) Gene (G) and protein (H,I) expression of FAK(PTK2) with FAK overexpression (oeFAK) in HGC-27 cells (n = 6 for panel (G), and n = 3 for panels (H,I)). (J–L) Gene (J) and protein (K,L) expression of FAK(PTK2) with FAK knockdown (shFAK-1/2) in HGC-27 cells (n = 4 for panel (J), and n = 3 for panels (K,L)).

(M,N) Representative images of wound healing, transwell and colony formation assays with FAK overexpression (M) and knockdown (N) in HGC-27 cells. (O-P) CCK-8 assay with FAK overexpression (O) and knockdown (P) in HGC-27 cells (n = 3). (Q,R) Quantification of functional assays with FAK overexpression (Q) and knockdown (R) in HGC-27 cells (n = 3). Scale bars: 100 μm for transwell images and 500 μm for wound healing images. Data are presented as mean ± s.d. Two-group comparisons were performed using Student's *t* test, and multiple groups were analyzed by one-way ANOVA. ns, not significant, * $p < 0.05$, ** $p < 0.01$, *** $p < 0.001$.

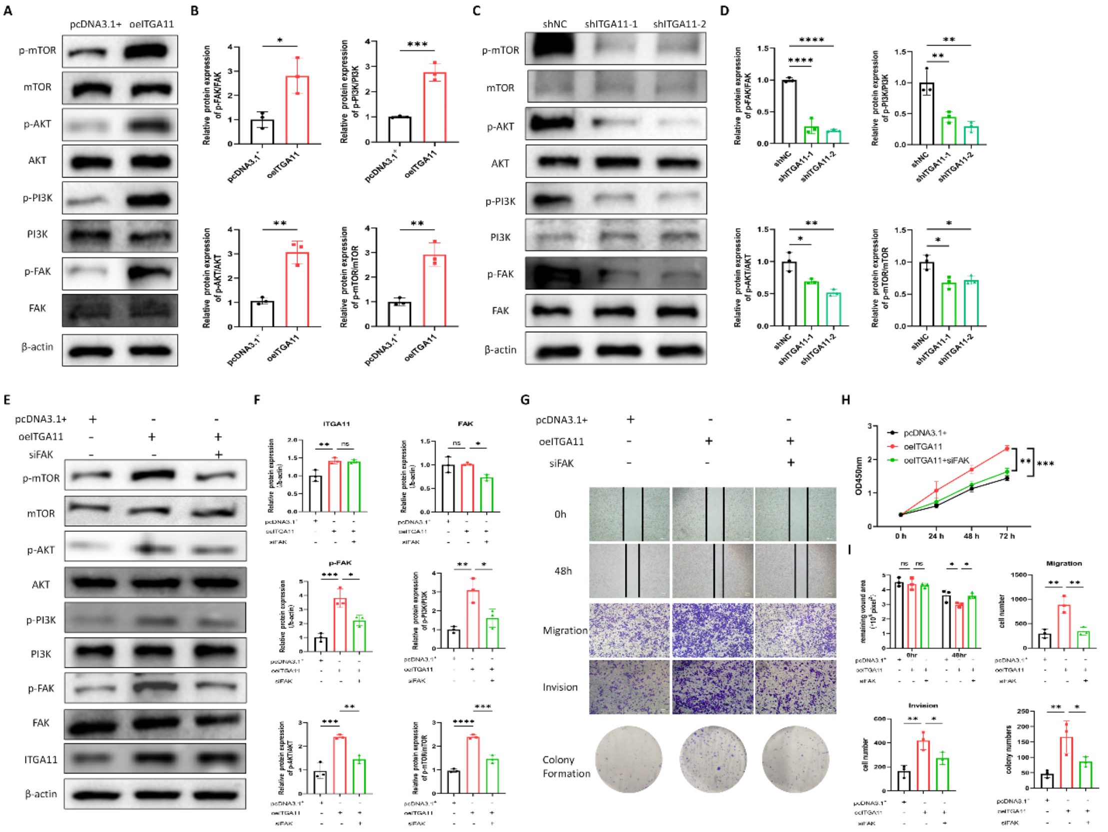

Figure S5. ITGA11 activates the FAK/PI3K/AKT/mTOR axis in HGC-27 cells. (A) Representative immunoblot images of FAK/PI3K/AKT/mTOR axis following ITGA11 overexpression in HGC-27 cells. (B) Quantification of FAK/PI3K/AKT/mTOR axis following ITGA11 overexpression in HGC-27 cells. (C) Representative immunoblot images of FAK/PI3K/AKT/mTOR axis following ITGA11 knockdown in HGC-27 cells. (D) Quantification of FAK/PI3K/AKT/mTOR axis following ITGA11 knockdown in HGC-27 cells. (E) Representative immunoblot images of ITGA11 and FAK/PI3K/AKT/mTOR axis following ITGA11 overexpression and FAK knockdown in HGC-27 cells. (F) Quantification of ITGA11

and FAK/PI3K/AKT/mTOR axis following ITGA11 overexpression and FAK knockdown in HGC-27 cells. (G) Representative images of wound healing, transwell and colony formation assays following ITGA11 overexpression and FAK knockdown in HGC-27 cells. (H) CCK-8 assay following ITGA11 overexpression and FAK knockdown in HGC-27 cells. (I) Quantification of functional assays following ITGA11 overexpression and FAK knockdown in HGC-27 cells. Scale bars: 100 μm for transwell images and 500 μm for wound healing images. Data are presented as mean ± s.d. (n = 3 per group). Two-group comparisons were performed using Student's *t* test, and multiple groups were analyzed by one-way ANOVA. ns, not significant, * $p < 0.05$, ** $p < 0.01$, *** $p < 0.001$, **** $p < 0.0001$.

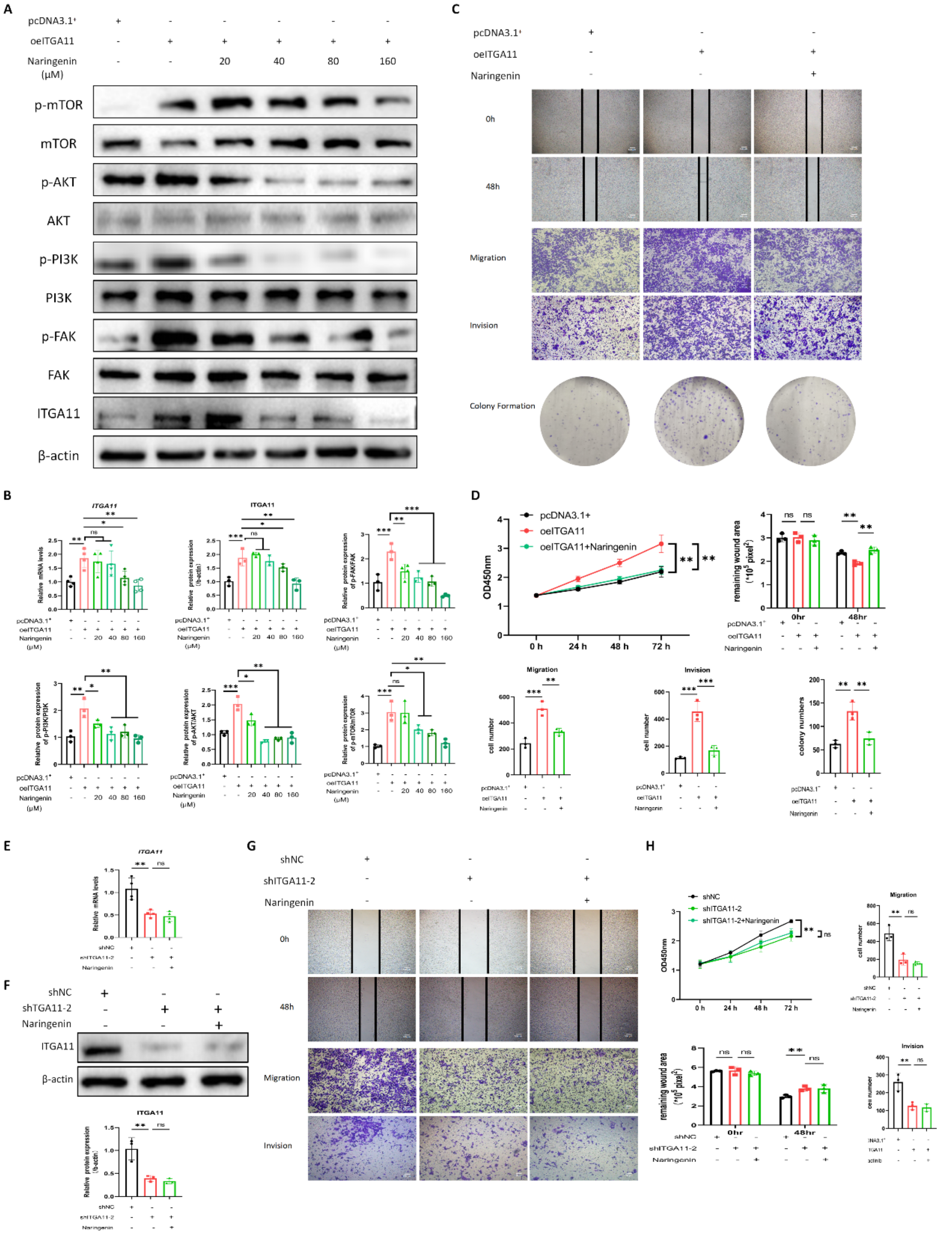

Figure S6. Naringenin suppresses ITGA11-associated FAK/PI3K/AKT/mTOR signaling and malignant phenotypes in HGC-27 cells. (A) Representative immunoblot images of ITGA11 and FAK/PI3K/AKT/mTOR axis following ITGA11 overexpression and Naringenin treatment (20, 40, 80, and 160 μM for 24 h) in HGC-27 cells. (B) Quantification of gene (n = 4) and protein (n = 3) expression of ITGA11 and FAK/PI3K/AKT/mTOR axis following ITGA11 overexpression and Naringenin treatment (20, 40, 80, and 160 μM for 24 h) in HGC-27 cells.

(C) Representative images of wound healing, transwell and colony formation assays following ITGA11 overexpression and Naringenin treatment (40 μM; 24 h) in HGC-27 cells. (D) Quantification of functional assays following ITGA11 overexpression and Naringenin treatment (40 μM; 24 h) in HGC-27 cells (n = 3). (E,F) Gene (E) and protein (F) expression of ITGA11 following ITGA11 knockdown and Naringenin treatment (40 μM; 24 h) in HGC-27 cells (n = 4 for panel (E), and n = 3 for panel (F)). (G) Representative images of wound healing and transwell assays following ITGA11 knockdown and Naringenin treatment (40 μM; 24 h) in HGC-27 cells. (H) Quantification of functional assays following ITGA11 knockdown and Naringenin treatment (40 μM; 24 h) in HGC-27 cells (n = 3). Scale bars: 100 μm for transwell images and 500 μm for wound healing images. Data are presented as mean ± s.d. Multiple groups were analyzed by one-way ANOVA. ns, not significant, * $p < 0.05$, ** $p < 0.01$, *** $p < 0.001$.
