# Supplementary material for: Naringenin, a Food-Derived Flavanone, Suppresses ITGA11-Associated Gastric Cancer Progression via the FAK/PI3K/AKT/mTOR Axis
Source: Cancers (Basel). 2026 May 24;18(11):1712. doi: 10.3390/cancers18111712 (PMC13255981; doi:10.3390/cancers18111712)
Supplement: Supplementary file 1 [file cancers-18-01712-s001.zip › Table S1.pdf]

Table S1. Baseline characteristics of the patient cohort (N = 60).

Characteristics	ITGA11 with low expression N=30	ITGA11 with high expression N=30	P value
**Sex**			0.426
Male	17 (56.7%)	20 (66.7%)	
Female	13 (43.3%)	10 (33.3%)	
**Age (years)**	57.4 ± 9.6	57.6 ± 9.9	0.947
**Maximum diameter of tumor (mm)**	23.8 ± 9.51	29.7 ± 7.4	0.009
**Endovascular invasion**			0.793
No	12 (40.0%)	13 (43.3%)	
Yes	18 (60.0%)	17 (56.7%)	
**Perineural invasion**			0.438
No	13 (43.3%)	16 (53.3%)	
Yes	17 (56.7%)	14 (46.7%)	
**Ki-67 level (%)**	41.7 ± 17.8	53.8 ± 17.0	**0.009***
**Lauren classification**			1.000
Diffuse	14 (46.7%)	14 (46.7%)	
Intestinal	16 (53.3%)	16 (53.3%)	
**Histological differentiation**			**0.004***
Low	14 (46.7%)	14 (46.7%)	
Medium	6 (20.0%)	15 (50.0%)	
High	10 (33.3%)	1 (3.3%)	

Characteristics	ITGA11 with low expression N=30	ITGA11 with high expression N=30	P value
**T stage**			0.120
1	4 (13.3%)	3 (10.0%)	
2	14 (46.7%)	6 (20.0%)	
3	5 (16.7%)	8 (26.7%)	
4	7 (23.3%)	13 (43.3%)	
**N stage**			**0.024***
0	13 (43.3%)	5 (16.7%)	
1	10 (33.3%)	8 (26.7%)	
2	5 (16.7%)	7 (23.3%)	
3	2 (6.7%)	10 (33.3%)	

Values are presented as mean ± s.d. or n (%). Percentages are calculated within each group. P values were calculated using Student's t-test or χ^2 test as appropriate.
