# Supplementary material for: Naringenin, a Food-Derived Flavanone, Suppresses ITGA11-Associated Gastric Cancer Progression via the FAK/PI3K/AKT/mTOR Axis
Source: Cancers (Basel). 2026 May 24;18(11):1712. doi: 10.3390/cancers18111712 (PMC13255981; doi:10.3390/cancers18111712)
Supplement: Supplementary file 1 [file cancers-18-01712-s001.zip › Table S2.pdf]

Table S2. Main reagents in study.

Name	Catalog number	Manufacturer	Country
Fetal bovine serum	A5256701	Gibco	USA
Roswell Park Memorial Institute 1640 (RPMI 1640) medium	SH30605.01	HyClone	USA
Dulbecco's Modified Eagle Medium (DMEM medium)	SH30022.01	HyClone	USA
Phosphate Buffered Saline (PBS)	G4202	Servicebio	China
Polybrene	sc-134220	Santa Cruz Biotechnology	USA
Puromycin	sc-108071	Santa Cruz Biotechnology	USA
Trypsin	G4005	Servicebio	China
Ultrapure water (DEPC)	G3004	Servicebio	China
Penicillin-streptomycin	15140122	Gibco	USA
Dimethyl sulfoxide (DMSO)	GC20006	Servicebio	China
RNAiso Plus (Trizol)	15596018CN	Thermo	USA
2×Universal Blue SYBR Green qPCR Master Mix (with UDG)	G3328	Servicebio	China
SweScript RT II First Strand cDNA Synthesis Kit (inclusive of gDNA Remover)	G3333	Servicebio	China
0.1% crystal violet	BL802A	Biosharp	China

4% polyformaldehyde	G1101	Servicebio	China
Xylene	10023428	Sinopharm Group Co., Ltd.	China
Transwell chambers	3464	Corning	USA
BD Matrigel	356234	Solarbio	China
Hematoxylin and eosin	G1076	Servicebio	China
Cell Counting Kit-8	BS350B	Biosharp	China
Radio Immunoprecipitation Assay (RIPA) lysis buffer	BL504A	Biosharp	China
SDS-PAGE gel preparation kit	PG220, PG222, PG223	EpiZyme	China
BCA protein quantitative detection kit	BL521A SET	Biosharp	China
Horseradish peroxidase (HRP)-conjugated secondary antibody	G1213	Servicebio	China
Polyvinylidene Fluoride(PVDF)membranes	IPVH00010	Millipore	USA
Non-fat milk	GC310001	Servicebio	China
Alexa Fluor 488-conjugated secondary antibody	GB25301	Servicebio	China
4',6-diamidino-2-phenylindole	G1012	Servicebio	China
Tris-Buffered Saline with Tween 20 (TBST)	G2150	Servicebio	China

Diaminobenzidine	G1212	Servicebio	China
Hematoxylin	G1004	Servicebio	China
Bovine serum albumin	GC305010	Servicebio	China
ITGA11 antibody	68350	CST	USA
p-FAK antibody	3283	CST	USA
FAK antibody	3285	CST	USA
p-PI3K (p85 (Tyr458)/p55 (Tyr199)) antibody	4228	CST	USA
PI3K antibody	Abs147226	Absin	China
p-AKT Ser473 antibody	28731-1-AP	Proteintech	China
AKT antibody	Ab8805	Abcam	USA
p-mTOR antibody	5536	CST	USA
mTOR antibody	2983	CST	USA
Bax antibody	2772	CST	USA
Bcl-2 antibody	3498	CST	USA
Vimentin antibody	ab92547	Abcam	USA
E-cadherin antibody	ab40772	Abcam	USA
N-cadherin antibody	ab76011	Abcam	USA
Ki-67 antibody	27309-1-AP	Proteintech	China
β-actin antibody	GB15003	Servicebio	China
HRP-conjugated secondary antibody	GB23303	Servicebio	China

Enhanced chemiluminescence (ECL) kit	BL523B	Biosharp	China
pLKO.1 plasmid	8453	Addgene	USA
pcDNA3.1+ plasmid	V79020	Thermo	USA
Lipofectamine 3000 reagent	TL301-01	Vazyme	China
Restriction endonucleases	ER0271	Thermo	USA
Cell genome DNA extraction kit	G3633	Servicebio	China
Mini Plasmid Kit	DP118	TIANGEN	Germany
Naringenin	HY-N0100	MedChemExpress	China
Defactinib	HY-12289	MedChemExpress	China
Pronase	HY-114158 A	MedChemExpress	China
