# Supplementary material for: Naringenin, a Food-Derived Flavanone, Suppresses ITGA11-Associated Gastric Cancer Progression via the FAK/PI3K/AKT/mTOR Axis
Source: Cancers (Basel). 2026 May 24;18(11):1712. doi: 10.3390/cancers18111712 (PMC13255981; doi:10.3390/cancers18111712)
Supplement: Supplementary file 1 [file cancers-18-01712-s001.zip › Table S3.pdf]

Table S3. Main instruments in study.

Name	Manufacturer and country	Type
Carbon dioxide constant temperature incubator	Singapore, ESCO	—
Bechtop	China, Haier	HCB-1300V
Electric thermostatic water tank	China, Jinghong	DK-500
Ice machine	Japan, SANYO	SIM-F140AY65
Pure water machine	Germany, Millipore	Milli-Q
Chemical balance	Germany, Sartorius	SQP
Inverted microscope	Japan, OLYMPUS	IX73
Upright microscope	Japan, OLYMPUS	BX53
Low temperature high speed centrifuge	USA, Thermo	Primo & Primo R
Microplate reader	USA, Perkin Elmer	EnSight
Nano Drop 2 Spectrophotometer	USA, Thermo	ND-ONE-W
Vertical electrophoresis apparatus	China, Servicebio	BV-2
Transfer electrophoresis apparatus	China, Servicebio	BT-2
Chemiluminescence analyzer	USA, Bio-Rad	ChemiDoc XRS+
PCR amplification apparatus	USA, Bio-Rad	T100
Real-time fluorescence quantitative PCR instrument	USA, Bio-Rad	CFX96
Chemiluminescence detector	USA, Promega	GLOMAX

DNA AGAR gel electrophoresis tank	China, Beyotime	EEP105
Confocal microscope	Germany, Leica	TCS SP8
