# Supplementary material for: Naringenin, a Food-Derived Flavanone, Suppresses ITGA11-Associated Gastric Cancer Progression via the FAK/PI3K/AKT/mTOR Axis
Source: Cancers (Basel). 2026 May 24;18(11):1712. doi: 10.3390/cancers18111712 (PMC13255981; doi:10.3390/cancers18111712)
Supplement: Supplementary file 1 [file cancers-18-01712-s001.zip › Table S4.pdf]

Table S4. Primer, shRNA, and siRNA sequences used in the study.

Gene	Sequence (5′-3′)
ITGA11 FP	CCTGCAGTTTGCCAGCTTG
ITGA11 RP	CTCCTCGTTCACACACTCAATG
PTK2(FAK) FP	GGACATTGCTGCTCGGAATG
PTK2(FAK) RP	CAAATCTCCAAATTGGCCTCC
β-actin FP	GGTTTTTCCTGTGGAGGAAA
β-actin RP	GGCATGAACCGTTCCCAGAT
ITGA11 CDS FP-1	CCCAAGCTTATGGACCTGCCCAGGGGC
ITGA11 CDS RP-1	CCGCTCGAGTCACTCCAGCACTTTGGGGGT
PTK2(FAK) CDS FP-1	CCCAAGCTTATGGCAGCTGCTTACCTTGAC
PTK2(FAK) CDS RP-1	CCGCTCGAGTCAGTGTGGTCTCGTCTGCCC
shITGA11 FP-1	CCGGGCACGACATCAGTGGCAATAACTCGAGTTATTGCCACTGATGTCGTGCTTTTTG
shITGA11 RP-1	AATTCAAAAAGCACGACATCAGTGGCAATAACTCGAGTTATTGCCACTGATGTCGTGC
shITGA11 FP-2	CCGGGCTCTTACTTTGGGAGTGAAACTCGAGTTTCACTCCCAAAGTAAGAGCTTTTTG
shITGA11 RP-2	AATTCAAAAAGCTCTTACTTTGGGAGTGAAACTCGAGTTTCACTCCCAAAGTAAGAGC
shPTK2 (FAK) FP-1	CCGGCCGATTGGAAACCAACATATACTCGAGTATATGTTGGTTTCCAATCGGTTTTTG
shPTK2 (FAK) RP-1	AATTCAAAAACCGATTGGAAACCAACATATACTCGAGTATATGTTGGTTTCCAATCGG
shPTK2 (FAK) FP-2	CCGGGATGTTGGTTTAAAGCGATTTCTCGAGAAATCGCTTTAAACCAACATCTTTTTG
shPTK2 (FAK) RP-2	AATTCAAAAAGATGTTGGTTTAAAGCGATTTCTCGAGAAATCGCTTTAAACCAACATC
siPTK2 (FAK) FP-1	GUAUUGGACCUGCGAGGGA
siPTK2 (FAK)RP-1	UCCCUCGCAGGUCCAAUAC

Restriction endonuclease and vectors

Knock-down vector: pLKO.1 plasmid;

Restriction endonuclease of pLKO.1 plasmid: EcoR I and Age I;
