# Supplementary material for: Naringenin, a Food-Derived Flavanone, Suppresses ITGA11-Associated Gastric Cancer Progression via the FAK/PI3K/AKT/mTOR Axis
Source: Cancers (Basel). 2026 May 24;18(11):1712. doi: 10.3390/cancers18111712 (PMC13255981; doi:10.3390/cancers18111712)
Supplement: Supplementary file 1 [file cancers-18-01712-s001.zip › Table S5.pdf]

Table S5. Effect sizes for all *p*-values.

Figure	P-values	Statistical methods	$\lvert$ Effect sizes $\rvert$
1F	2.76e-123	R	0.881
1H	3.09E-79	R	0.784
1I	< 0.001	R	0.842
1J	< 0.001	R	0.777
1K	< 0.001	R	0.7990
1L	< 0.001	R	0.826
1M	< 0.001	R	0.831
S1D	7.4e-139	R	0.888
S1F	1.03e-93	R	0.805
2E	0.008	HR	1.790
2F	0.107	HR	1.350
2G	0.071	HR	1.590
2I	0.26	HR	1.200
2M	0.062	HR	1.400
2O	0.016	HR	1.510
2P	< 0.001	R	0.189
S2F	0.068	HR	0.730
S2G	0.164	HR	0.770
S2H	0.397	HR	1.150
S2I	0.237	HR	0.820
S2J	0.287	HR	0.820
S2K	0.005	R	0.144
S2L	< 0.001	R	0.366
S2M	< 0.001	R	0.305
S2N	< 0.001	R	0.241
S2O	0.304	R	-0.053
S2P	< 0.001	R	0.199
3B	< 0.001	Cohen's d	1.06
3C	0.025	HR	1.87
3D	< 0.05	Cohen's d	2.67
3F	< 0.05	Cohen's d	1.01
3H	< 0.001	Cohen's d	3.05
3I	-	η^2	0.884
3K	-	η^2	0.946
3L	< 0.01	Cohen's d	2.28
3N	< 0.05	Cohen's d	3.70
3O	-	η^2	0.886
3Q	-	η^2	0.914
3R	< 0.001	Cohen's d	2.37
3S	-	η^2	0.899
3U-1	< 0.01	Cohen's d	5.03
3U-2	< 0.05	Cohen's d	2.90

3U-3	< 0.05	Cohen's d	3.74
3U-4	< 0.01	Cohen's d	4.58
3W-1	-	η^2	0.819
3W-2	-	η^2	0.864
3W-3	-	η^2	0.908
3W-4	-	η^2	0.840
S3A	< 0.01	Cohen's d	2.29
S3C	< 0.01	Cohen's d	6.78
S3D	< 0.01	Cohen's d	4.10
S3E	-	η^2	0.883
S3G	-	η^2	0.730
S3H	-	η^2	0.837
S3K-1	< 0.0001	Cohen's d	6.15
S3K-2	< 0.01	Cohen's d	3.93
S3K-3	< 0.01	Cohen's d	5.09
S3K-4	< 0.01	Cohen's d	2.84
S3L-1	-	η^2	0.702
S3L-2	-	η^2	0.949
S3L-3	-	η^2	0.895
S3L-4	-	η^2	0.933
4A	ns	Cohen's d	0.21
4C	< 0.01	Cohen's d	6.37
4D	-	η^2	0.094
4F	-	η^2	0.779
4G	< 0.01	Cohen's d	3.25
4I	< 0.05	Cohen's d	2.06
4J	-	η^2	0.697
4L	-	η^2	0.825
4M	< 0.01	Cohen's d	1.93
4O	< 0.05	Cohen's d	4.70
4P	-	η^2	0.889
4R	-	η^2	0.803
4U	< 0.001	Cohen's d	2.36
4V	-	η^2	0.943
4W-1	< 0.01	Cohen's d	4.13
4W-2	< 0.01	Cohen's d	4.57
4W-3	< 0.05	Cohen's d	3.58
4W-4	< 0.01	Cohen's d	4.02
4X-1	-	η^2	0.872
4X-2	-	η^2	0.803
4X-3	-	η^2	0.782
4X-4	-	η^2	0.854
S4A	ns	Cohen's d	0.11
S4C	< 0.05	Cohen's d	2.59

S4D	-	η^2	0.027
S4F	-	η^2	0.787
S4G	< 0.01	Cohen's d	1.86
S4I	< 0.05	Cohen's d	2.29
S4J	-	η^2	0.839
S4L	-	η^2	0.795
S4O	< 0.01	Cohen's d	2.42
S4P	-	η^2	0.912
S4Q-1	< 0.01	Cohen's d	7.97
S4Q-2	< 0.01	Cohen's d	3.88
S4Q-3	< 0.001	Cohen's d	7.50
S4Q-4	< 0.05	Cohen's d	2.27
S4R-1	-	η^2	0.825
S4R-2	-	η^2	0.926
S4R-3	-	η^2	0.900
S4R-4	-	η^2	0.732
5B-1	< 0.001	Cohen's d	7.61
5B-2	< 0.01	Cohen's d	6.28
5B-3	< 0.05	Cohen's d	2.85
5B-4	< 0.01	Cohen's d	4.07
5D-1	-	η^2	0.847
5D-2	-	η^2	0.891
5D-3	-	η^2	0.748
5D-4	-	η^2	0.949
5F-1	-	η^2	0.841
5F-2	-	η^2	0.893
5F-3	-	η^2	0.953
5F-4	-	η^2	0.916
5F-5	-	η^2	0.780
5F-6	-	η^2	0.624
5H	-	η^2	0.817
5I-1	-	η^2	0.839
5I-2	-	η^2	0.914
5I-3	-	η^2	0.884
5I-4	-	η^2	0.793
S5B-1	< 0.05	Cohen's d	3.254
S5B-2	< 0.05	Cohen's d	2.142
S5B-3	< 0.05	Cohen's d	1.829
S5B-4	< 0.01	Cohen's d	3.624
S5D-1	-	η^2	0.954
S5D-2	-	η^2	0.731
S5D-3	-	η^2	0.804
S5D-4	-	η^2	0.788
S5F-1	-	η^2	0.843

S5F-2	-	η^2	0.907
S5F-3	-	η^2	0.876
S5F-4	-	η^2	0.925
5F-5	-	η^2	0.955
S5F-6	-	η^2	0.919
S5H	-	η^2	0.869
S5I-1	-	η^2	0.815
S5I-2	-	η^2	0.891
S5I-3	-	η^2	0.913
S5I-4	-	η^2	0.962
6B-1	-	η^2	0.794
6B-2	-	η^2	0.924
6B-3	-	η^2	0.826
6B-4	-	η^2	0.887
6B-5	-	η^2	0.827
6D	-	η^2	0.906
6E-1	-	η^2	0.791
6E-2	-	η^2	0.715
6E-3	-	η^2	0.861
6F	-	η^2	0.964
6I-1	-	η^2	0.884
6I-2	-	η^2	0.838
6I-3	-	η^2	0.827
7C	-	η^2	0.941
7E	-	η^2	0.927
7F	-	η^2	0.754
7G	-	η^2	0.692
7H	-	η^2	0.621
7J-1	-	η^2	0.728
7J-2	-	η^2	0.691
7J-3	-	η^2	0.664
7J-4	-	η^2	0.824
7J-5	-	η^2	0.916
7L	-	η^2	0.879
7M-1	-	η^2	0.825
7M-2	-	η^2	0.857
7M-3	-	η^2	0.904
7M-4	-	η^2	0.828
7O	-	η^2	0.937
S6B-1	-	η^2	0.684
S6B-2	-	η^2	0.701
S6B-3	-	η^2	0.638
S6B-4	-	η^2	0.725
S6B-5	-	η^2	0.618

S6B-6	-	η^2	0.753
S6D-1	-	η^2	0.891
S6D-2	-	η^2	0.859
S6D-3	-	η^2	0.908
S6D-4	-	η^2	0.811
S6D-5	-	η^2	0.839
S6E	-	η^2	0.937
S6F	-	η^2	0.874
S6H-1	-	η^2	0.793
S6H-2	-	η^2	0.726
S6H-3	-	η^2	0.984
S6H-4	-	η^2	0.863
8C	< 0.01	Cohen's d	5.64
8D	< 0.001	Cohen's d	6.10
8E	< 0.01	Cohen's d	7.07
8F	< 0.001	Cohen's d	10.0
8I	< 0.05	Cohen's d	2.78
8K	< 0.05	Cohen's d	3.56
8L	< 0.01	Cohen's d	4.67
8N	< 0.05	Cohen's d	3.40

HR: Hazard Ratio; R: Correlation Coefficient; η^2: Eta-squared
