# Supplementary material for: Naringenin, a Food-Derived Flavanone, Suppresses ITGA11-Associated Gastric Cancer Progression via the FAK/PI3K/AKT/mTOR Axis
Source: Cancers (Basel). 2026 May 24;18(11):1712. doi: 10.3390/cancers18111712 (PMC13255981; doi:10.3390/cancers18111712)
Supplement: Supplementary file 1 [file cancers-18-01712-s001.zip › Table S6.pdf]

Table S6. Univariate and multivariate Cox regression analyses in the TCGA-STAD cohort (N = 370).

Characteristics	Total(N)	Univariate analysis		Multivariate analysis	
		Hazard ratio (95% CI)	P value	Hazard ratio (95% CI)	P value
**Age**	367				
≤65	163	Reference		Reference	
> 65	204	1.620 (1.154 - 2.276)	**0.005**	1.910 (1.325 - 2.754)	**< 0.001**
**Gender**	370				
Male	237	Reference			
Female	133	0.789 (0.554 - 1.123)	0.188		
**Histological type**	369				
Not Otherwise Specified	202	Reference			
Diffuse Type&Mucinous Type&Papillary Type&Signet Ring Type&Tubular Type	167	0.815 (0.586 - 1.132)	0.222		
**Pathologic T stage**	362				
T1-T2	96	Reference		Reference	
T3-T4	266	1.719 (1.131 - 2.612)	**0.011**	1.344 (0.851 - 2.121)	0.205
**Pathologic N stage**	352				
N0	107	Reference		Reference	
N1-N3	245	1.925 (1.264 - 2.931)	**0.002**	1.787 (1.137 - 2.807)	**0.012**
**Pathologic M stage**	352				
M0	327	Reference		Reference	
M1	25	2.254 (1.295 - 3.924)	**0.004**	2.445 (1.358 - 4.402)	**0.003**
**ITGA11**	370				

Characteristics	Total(N)	Univariate analysis		Multivariate analysis	
		Hazard ratio (95% CI)	P value	Hazard ratio (95% CI)	P value
Low	185	Reference		Reference	
High	185	1.450 (1.040 - 2.022)	**0.028**	1.639 (1.152 - 2.330)	**0.006**
