# Supplementary material for: Naringenin, a Food-Derived Flavanone, Suppresses ITGA11-Associated Gastric Cancer Progression via the FAK/PI3K/AKT/mTOR Axis
Source: Cancers (Basel). 2026 May 24;18(11):1712. doi: 10.3390/cancers18111712 (PMC13255981; doi:10.3390/cancers18111712)
Supplement: Supplementary file 1 [file cancers-18-01712-s001.zip › Table S7.pdf]

Table S7. Univariate and multivariate Cox regression analyses in our institutional cohort (N = 60).

Characteristics	Total(N)	Univariate analysis		Multivariate analysis	
		Hazard ratio (95% CI)	P value	Hazard ratio (95% CI)	P value
**Sex**	60				
Male	37	Reference			
Female	23	1.713 (0.866 - 3.387)	0.122		
**Endovascular invasion**	60				
No	25	Reference			
Yes	35	1.777 (0.868 - 3.637)	0.116		
**Nerve invasion**	60				
No	29	Reference		Reference	
Yes	31	2.549 (1.226 - 5.299)	**0.012**	1.797 (0.823 - 3.921)	0.141
**Lauren's classification**	60				
Intestinal	32	Reference		Reference	
Diffuse	28	1.897 (0.938 - 3.838)	0.075	1.777 (0.846 - 3.730)	0.129
**T stage**	60				
1-2	27	Reference		Reference	
3-4	33	2.576 (1.183 - 5.612)	**0.017**	2.218 (1.572 - 3.131)	**0.023**
**N stage**	60				
0	18	Reference		Reference	
1-3	42	4.051 (2.593 - 6.329)	**< 0.001**	2.024 (1.292 - 4.131)	**< 0.001**
**ITGA11**	60				
Low	30	Reference		Reference	
High	30	2.205 (1.121 - 4.336)	**0.022**	2.024 (0.992 - 4.131)	0.053
