# Supplementary material for: Naringenin, a Food-Derived Flavanone, Suppresses ITGA11-Associated Gastric Cancer Progression via the FAK/PI3K/AKT/mTOR Axis
Source: Cancers (Basel). 2026 May 24;18(11):1712. doi: 10.3390/cancers18111712 (PMC13255981; doi:10.3390/cancers18111712)
Supplement: Supplementary file 1 [file cancers-18-01712-s001.zip › Table S8.pdf]

Table S8. Top fifteen candidate compounds with the lowest predicted binding energy for ITGA11.

Compound name	Predicted binding energy
Naringenin	-9.4
Ibuprofen	-9.3
Cilengitide	-9.0
Genistein	-8.9
Epigallocatechin	-8.9
Resveratrol	-8.3
Curcumin	-8.1
Valsartan	-7.9
Dexamethasone	-7.9
Metformin	-7.7
Rosuvastatin	-7.6
Atorvastatin	-7.5
Quercetin	-7.2
Aspirin	-6.4
Losartan	-6.3
