# Supplementary material for: Naringenin, a Food-Derived Flavanone, Suppresses ITGA11-Associated Gastric Cancer Progression via the FAK/PI3K/AKT/mTOR Axis
Source: Cancers (Basel). 2026 May 24;18(11):1712. doi: 10.3390/cancers18111712 (PMC13255981; doi:10.3390/cancers18111712)
Supplement: Supplementary file 1 [file cancers-18-01712-s001.zip › Table S9.pdf]

Table S9A. Body weight (g) of mice in Group oe-ITGA11 and oeITGA11 + Naringenin.

Group	Mouse ID	Day 0	Day 3	Day 6	Day 9	Day 12	Day 15	Day 18	Day 21	Day 21 (without tumor)
oeITGA11	OE-1	16.831	16.957	17.143	17.483	17.858	18.352	18.947	19.645	18.191
	OE-2	17.123	17.304	17.642	17.917	18.302	18.743	19.335	20.210	19.187
	OE-3	16.635	16.754	16.995	17.252	17.654	18.108	18.741	19.437	18.112
	OE-4	17.303	17.481	17.724	18.033	18.456	18.965	19.545	20.254	19.001
oeITGA11 +Naringenin	Drug-1	16.845	17.052	17.348	17.781	18.204	18.751	19.421	20.045	19.500
	Drug-2	17.318	17.483	17.823	18.248	18.729	19.228	19.837	20.475	20.221
	Drug-3	16.698	16.888	17.221	17.635	18.121	18.635	19.308	20.019	19.716
	Drug-4	17.426	17.682	18.063	18.496	18.953	19.504	20.146	20.728	20.318

Table S9B. Body weight (g) of mice in Group shNC and shITGA11-2.

Group	Mouse ID	Day 0	Day 3	Day 6	Day 9	Day 12	Day 15	Day 18	Day 21	Day 21 (without tumor)
shNC	NC-1	16.812	17.036	17.421	17.819	18.286	18.893	19.621	20.712	19.684
	NC-2	17.063	17.318	17.702	18.146	18.603	19.182	19.804	20.944	20.097
	NC-3	16.596	16.884	17.223	17.691	18.137	18.714	19.305	20.389	19.475
	NC-4	17.241	17.486	17.864	18.295	18.738	19.275	19.802	20.691	20.036
shITGA11-2	SH-1	16.734	17.041	17.469	17.938	18.492	19.113	19.829	20.762	20.608
	SH-2	17.128	17.463	17.882	18.351	18.923	19.546	20.181	21.084	20.982
	SH-3	16.489	16.822	17.214	17.706	18.248	18.861	19.525	20.493	20.284
	SH-4	17.306	17.612	18.037	18.486	19.063	19.684	20.233	21.016	20.843

Table S9C. Tumor volume (mm^3) of mice in Group oe-ITGA11 and oeITGA11 + Naringenin.

Group	Mouse ID	Day 9	Day 12	Day 15	Day 18	Day 21
oeITGA11	OE-1	4.603	127.483	395.224	530.462	1014.126
		4.459	138.774	393.904	517.351	1013.813
		4.237	115.751	372.811	526.278	974.143
	OE-2	2.353	128.921	352.798	573.636	951.383
		2.628	138.224	350.372	555.253	970.213
		2.762	139.649	342.896	567.150	943.203
	OE-3	10.403	147.311	280.822	546.416	744.981
		10.733	137.052	268.386	554.217	764.656
		10.450	155.466	279.765	548.294	755.042
	OE-4	5.894	80.824	267.362	401.657	824.143
		5.411	89.143	270.547	411.443	834.592
		5.738	87.406	284.144	408.086	846.956
oeITGA11+Naringenin	Drug-1	2.166	100.688	119.516	150.753	201.741
		2.202	108.658	124.811	142.288	215.622
		2.118	94.442	128.274	148.693	199.024
	Drug-2	10.256	138.169	232.754	272.004	321.576
		10.373	133.456	248.933	278.451	305.011
		10.252	139.498	236.256	283.249	321.052
	Drug-3	5.907	149.471	200.144	239.958	285.584
		5.738	138.792	197.114	240.311	276.067
		6.079	150.386	208.256	230.668	291.557
	Drug-4	6.032	124.551	156.886	183.989	217.750
		6.356	129.638	164.065	181.742	207.092
		6.029	127.834	151.460	172.127	222.583

Table S9D. Tumor volume (mm^3) of mice in Group shNC and shITGA11-2.

Group	Mouse ID	Day 9	Day 12	Day 15	Day 18	Day 21
shNC	NC-1	5.207	80.529	202.986	422.677	665.006
		5.556	81.421	210.999	411.793	659.675
		5.458	72.646	200.923	417.944	645.545
	NC-2	2.657	159.025	291.938	400.996	510.146
		2.806	152.251	282.934	388.216	491.582
		2.646	149.172	287.229	404.019	501.166
	NC-3	6.639	77.398	255.274	393.105	610.627
		6.271	70.163	273.667	383.059	602.249
		6.384	73.673	242.351	408.473	619.610
	NC-4	2.935	81.438	171.253	339.922	570.660
		2.829	87.091	168.597	343.557	575.447
		3.086	83.992	178.919	338.529	597.041
shITGA11-2	SH-1	2.269	9.902	10.044	50.383	105.454
		2.143	10.522	10.080	48.247	110.734
		2.131	10.432	9.2524	32.410	114.422
	SH-2	6.507	11.813	15.132	46.324	104.147
		6.221	12.697	14.032	48.553	114.372
		6.644	12.585	15.975	44.134	120.387
	SH-3	2.398	9.768	29.054	37.432	121.386
		2.504	10.359	28.749	40.679	131.646
		2.397	11.464	30.119	36.828	120.500
	SH-4	3.263	13.793	23.721	34.545	79.149
		3.401	12.722	23.024	35.708	78.795
		2.987	13.504	22.463	35.851	80.636
